# Composition-based machine learning for predicting and designing Mn^4+^-doped phosphors

**DOI:** 10.1039/d6ra00029k

**Published:** 2026-02-27

**Authors:** Ngo T. Que, Vu D. Huan, Le T. Duy, Vu N. Bao, Vu L. Minh, Mai X. Trang, Anh D. Phan, Pham T. Huy

**Affiliations:** a Phenikaa Institute for Advanced Study, Phenikaa University Hanoi 12116 Vietnam anh.phanduc@phenikaa-uni.edu.vn; b Faculty of Materials Science and Engineering, Phenikaa School of Engineering, Phenikaa University Hanoi 12116 Vietnam; c Faculty of Science, Engineering and Built Environment, School of Information Technology, Deakin University Australia; d Phenikaa School of Computing, Phenikaa University Hanoi 12116 Vietnam

## Abstract

We present a data-driven approach to predict the excitation wavelength, emission wavelength, and crystal field energy levels (^4^T_1_, ^4^T_2_) in Mn^4+^-doped phosphors based solely on elemental composition. For the first time, we construct the largest and most compherensive experimental dataset of Mn^4+^-activated phosphors to train and accurately predict the properties without relying on complex structural descriptors. Among several evaluated models, the K-Nearest Neighbors and Extra Trees Regressors achieved the highest accuracy for predicting excitation and emission wavelengths, respectively. Importantly, to evaluate generalization, we test these models on Eu^3+^-doped systems and achieve high predictive accuracy. An inverse design model is further developed to suggest candidate phosphor compositions for target optical outputs. By avoiding complex descriptors while preserving accuracy and interpretability, this work provides a foundation for theory-informed discovery of luminescent materials.

## Abstract 2

We collect the largest experimental dataset and apply machine learning to study composition–property relations in Mn^4+^-doped phosphors. The resulting models outperform prior studies and show good agreement with experimental data.

## Graphical abstract

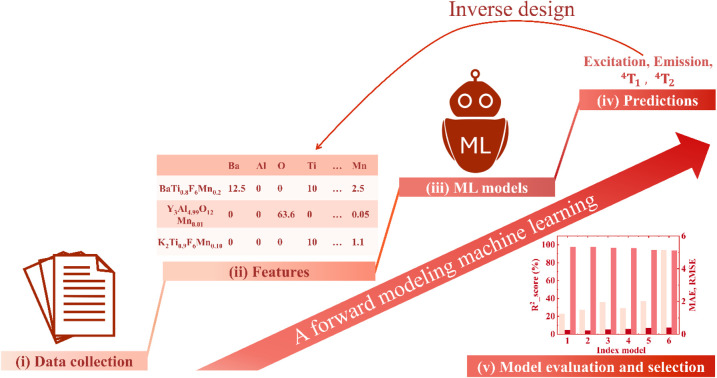

## 1. Introduction

Phosphor materials, also referred to as luminescent materials, are solids capable of converting various forms of energy into electromagnetic radiation beyond simple thermal emission.^1^ Due to their unique optical properties, phosphors have become increasingly significant in both academic research and practical applications. These materials are extensively employed across diverse applications such as display technologies,^2–4^ sensors,^5–7^ biomedical imaging,^7^ food quality analysis,^8^ health monitoring,^9,10^ and agriculture.^11^ Phosphors typically consist of a host material doped with luminescent activator ions. Among these activators, Mn^4+^ ions are well known as efficient red-light emitters because of their excellent thermal and chemical stability, low cost, and environmental friendliness.^1,2,11^ Mn^4+^-doped phosphors exhibit broadband excitation and sharp red emission lines that primarily arise from the ^2^E → ^4^A_2_ transition. Their optical properties can be interpreted using the Tanabe–Sugano diagram, which describes electronic transitions from the ^4^A_2_ ground state to the ^4^T_2_ and ^4^T_1_ excited states, as well as the ^2^E → ^4^A_2_ transition. To fully exploit their potential, it is essential to investigate key spectroscopic and electronic properties that govern their behaviors. In particular, determining the excitation wavelength, emission peak, and electronic transitions (^4^T_1_ and ^4^T_2_) is crucial for controlling the color, brightness, efficiency, and stability of these materials. By studying them, researchers can better design phosphors to meet specific requirements in lighting, imaging, sensing, and other advanced applications.

Accurately predicting the excitation wavelength, emission peak, and the ^4^T_1_ and ^4^T_2_ energy levels is not only crucial for optimizing the performance of phosphor materials, but also fundamental to advancing our theoretical understanding of their luminescent behavior.^12,13^ These physical quantities provide insights into the electronic structure and energy transfer mechanisms that govern how materials interact with light. In particular, the ^4^T_1_ and ^4^T_2_ energy levels are associated with specific electronic transitions of dopant ions, which influence both the position and intensity of emission bands. By analyzing these transitions, researchers can infer the local coordination environment, crystal field strength, and site symmetry of activator ions within the host lattice.^14^ This information is essential for selecting suitable host materials and dopants to achieve the desired emission and thermal stability.^15^ Similarly, the emission peak indicates the energy of photons released as excited electrons return to lower energy states, while the excitation wavelength represents the energy required to trigger this luminescent process. Knowing these two quantities helps select suitable excitation sources, enhance color quality, and evaluate optical efficiency of phosphor materials.^16^ These understanding plays a key role in facilitating fabrication and application by identifying promising material systems prior to synthesis.

While experimental techniques such as photoluminescence, photoluminescence excitation, time-resolved luminescence, and temperature-dependent emission analyses have been widely used,^1,17–19^ they require costly equipment, demanding sample preparation, specialized environments, and time-consuming procedures. These problems limit their use in high-throughput or exploratory studies. In contrast, theoretical methods based on using machine learning (ML) or deep learning (DL) to analyze database give fast and accurate estimation of key optical parameters using only compositional or structural data.^20,21^ These computational approaches dramatically reduce time and resources needed to screen and optimize phosphor materials.

Machine learning and deep learning are increasingly being applied to the research and design of luminescent materials, particularly phosphors used in LED technologies.^22–34^ These models allow us to predict the emission wavelength,^23,24,27,30–32^ thermal quenching temperature,^27,29,31^ spectral bandwidth, and quantum yield^31^ based on a material's composition and crystal structure. Algorithms including artificial neural networks (ANN),^33^ Gradient Boosting Regression,^23,26,30,31^ and Random Forest^22–25,34^ have shown strong performance in accelerating the discovery and optimization of phosphor materials. Among various dopants, europium (Eu)-doped phosphors are the most widely studied using machine learning because a large amount of experimental data is available for them.^28–31,33,34^ In contrast, other dopant systems remain underexplored because of the lack of comprehensive and publicly available data. Another major challenge in this area is the use of many input features, which is typically between 50 and 150, for most machine learning models.^23,26–29,31–34^ These features often include detailed information at the atomic level such as atomic structure data,^29^ ionic radii,^23,33,34^ atomic weights,^32,34^ and electronegativity values.^23,24,27,30,31,34^ Furthermore, collecting full data for each material is often difficult and takes time.

These challenges raise important questions about how to improve the accuracy and usefulness of machine learning models for designing phosphor materials. (1) Can the excitation wavelength, emission peak, ^4^T_1_ and ^4^T_2_ energy levels of Mn^4+^-doped phosphors be accurately predicted using only elemental composition without relying on experimental properties or complex descriptors? (2) Which machine learning algorithms provide the best predictive accuracy for excitation and emission properties of Mn^4+^-doped phosphors? (3) Are models trained solely on Mn^4+^-doped compositions transferable to other dopant systems with different luminescent behavior? (4) Lastly, can an inverse-design approach be developed to propose candidate phosphor compositions based on desired excitation and emission wavelengths? Answering these questions will help create more efficient and generalizable machine-learning tools to better discover and design new phosphor materials.

In this work, we address these challenges by developing a data-driven approach to predict and design phosphor materials. We first collect experimental data on Mn^4+^-doped phosphors and use it to train machine learning models that predict the excitation wavelength, the emission peak, and the wavelength of the ^4^T_1_ and ^4^T_2_ transition based solely on chemical composition. To evaluate the generalizability of our approach, we apply the trained models to predict the optical properties of Eu^3+^-doped phosphors. Once reliable forward prediction models are established, we construct an inverse design algorithm to suggest phosphor compositions based on target properties.

## 2. Theoretical background

**Fig. 1:**
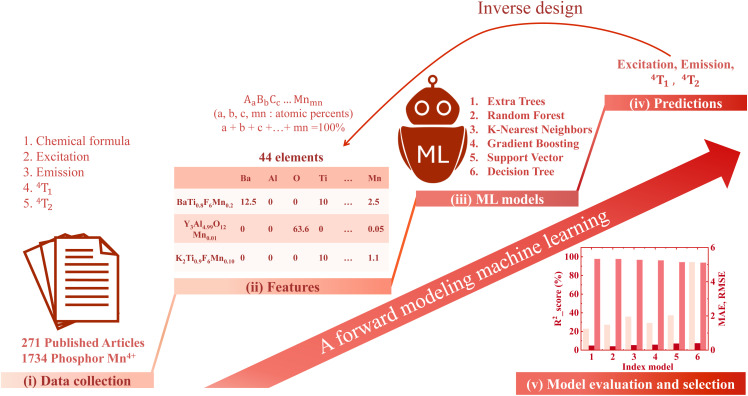
(Color online) Workflow for predicting the excitation, emission, and energy levels ^4^T_1_, ^4^T_2_ of Mn^4+^-activated phosphor using six machine learning algorithms, followed by an inverse design approach to determine the optimal chemical formula of the phosphor material.

Our modeling workflow consists of six main steps as illustrated in Fig. 1. First, a dataset of Mn^4+^-doped phosphors is collected from peer-reviewed papers and books. Second, the data is preprocessed and transformed into numerical features based on elemental composition. In the third step, six different machine learning algorithms including Extra Trees (ET), Random Forest (RF), K-Nearest Neighbors (KNN), Gradient Boosting (XGB), Support Vector Regression (SVR), and Decision Trees (DT) are trained to predict the excitation wavelength, emission wavelength, and the ^4^T_1_ and ^4^T_2_ energy levels. The fourth step uses the coefficient of determination (*R*^2^), root-mean-square error (RMSE), and mean absolute error (MAE) to evaluate model performance. In the fifth step, model generalization is tested using an independent dataset of Eu^3+^-doped phosphors to assess transferability across different dopant systems. Finally, an inverse design model is constructed to propose new phosphor compositions that match user-defined excitation and emission targets.

### 2.1. Data collection

Two datasets were used to develop and validate the predictive models. The first dataset, constructed for the first time, contains information on 1734 Mn^4+^-doped phosphors reported in 271 published studies. In all cases, the activator charge state is taken from experimental papers where Mn is explicitly identified as Mn^4+^. It was collected to train machine learning models to predict excitation wavelength, emission wavelength, and the ^4^T_1_ and ^4^T_2_ energy levels. The host materials in this dataset consist of three to six elements including 373 ternary, 1089 quaternary, 252 quinary, and 20 senary compositions. The experimental data covered broad ranges: excitation wavelengths from 253 to 500 nm, emission wavelengths from 600 to 731 nm, ^4^T_1_ energy levels from 224 to 487 nm, and ^4^T_2_ energy levels from 274 to 680 nm. In total, the dataset includes 1734 data points for excitation and emission wavelengths and 1701 data points for ^4^T_1_ and ^4^T_2_ energy levels. The second dataset, used to test model generalization, consists of 1665 data points for excitation and emission wavelengths of Eu-doped phosphors taken from ref. 31. Following the source experimental literature, the Eu activator in this set is categorized as Eu^3+^. All datasets used in this study are provided in the SI.

### 2.2. Feature engineering

After collecting the datasets, the chemical compositions were converted into a normalized input format suitable for the predictive models. Each material was expressed as A_a_B_b_C_c_⋯Mn_mn_, where A, B, C, and Mn are the constituent elements, and a, b, c, and mn are their atomic percentages. These percentages were calculated by dividing the number of atoms of each element by the total number of atoms in the formula to ensure that the sum equals 100%. The resulting representation is a fixed-length vector comprising 44 features, each corresponding to a possible element, where the feature value is set to zero for elements not present in the composition.

### 2.3. Machine learning modeling

The dataset was randomly divided into training and testing subsets using an 80 : 20 ratio, which is commonly used in machine learning studies. In our previous work,^21^ we examined different splitting ratios ranging from 60 : 40 to 90 : 10 and found that increasing the proportion of training data generally improves the predictive accuracy. However, the improvement becomes marginal when the training set increases from 80 to 90%. To validate our model and avoid overfitting, a 5-fold cross-validation was used during training. Moreover, we performed hyper parameter optimization for each machine learning algorithm using a randomized search approach. In our work, all regression models and the GridSearchCV-based hyperparameter tuning were implemented using the scikit-learn library.^35,36^ This approach scans a broad range of parameter values and selects those that give the best results under cross-validation and improve predictive accuracy.

The model performance was evaluated using the coefficient of determination (*R*^2^), root mean squared error (RMSE), and mean absolute error (MAE), defined as1
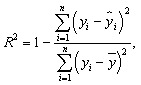
2
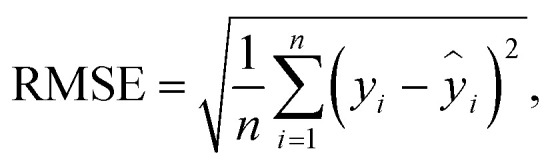
3
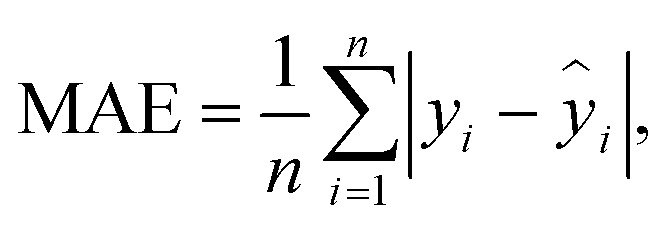
where *y*_*i*_ and *ŷ_i_* are the observed and predicted values for the ith data point, *ȳ* is the mean of the observed values, and *n* is the total number of data points. A model with higher *R*^2^ and lower RMSE and MAE values is considered to have better predictive accuracy.

## 3. Results and discussion

Table 1 provides a summary of previous studies that applied machine learning and deep learning models to predict various properties of phosphor materials. These works include both experimental and DFT-based datasets and use a range of algorithms to model optical and thermal properties such as emission wavelength, thermal quenching temperature, lifetime, and quantum efficiency for different dopant systems. The values in Table 1 serve only as literature benchmarks and are not used as training or test data in our present study. While these studies show the potential of machine learning for phosphor research, they are generally limited by small datasets, narrow dopant types, or the use of complex descriptors that are not always available for new materials. The wide range of predictive accuracy, even when detailed structural or DFT-derived descriptors are employed, indicates that phosphor structure–property relationships remain challenging to capture accurately. There is still significant room for improvement in both descriptors and models. Notably, none of the prior studies analyzed a large and comprehensive dataset for Mn^4+^-doped phosphors. In this work, we collect such dataset for the first time and use it to develop composition-based machine learning models that accurately predict the excitation wavelength, emission peak, and ^4^T_1_ and ^4^T_2_ energy levels, as well as to facilitate inverse design of new phosphor compositions.

**Table 1:** Summary of datasets, ML and DL models, and their corresponding *R*^2^, RMSE and MAE values reported in previous studies for predicting various properties of phosphor materials

Size of data	Type of data	Type of doping	Predicted	DL/ML models	*R* ^2^	RMSE	MAE	Reference
39	Experiment	Mn^4+^	^2^ *E* energy (cm^−1^) (lowest energy excited state)	Linear regression	0.95	149.99	89.33	22
				Robust regression	0.94	153.73	95.68	
				Lasso regression	0.95	149.86	91.05	
				Ridge regression	0.93	168.07	133.81	
				ElasticNet	0.66	383.54	281.9	
				DT	0.31	541.56	401.17	
				RF	0.72	348.04	249.26	
116	Experiment	Mn^4+^	Emission peak (nm)	XGB	0.71	14.25	9.88	23
				RF	0.80	16.65	10.77	
				Lasso regression	0.64	17.09	11.37	
				Ridge regression	0.69	18.93	12.82	
				KNN	0.85	13.08	8.13	
				SVR	0.81	13.6	9.39	
33	Experiment	Mn^4+^	Emission peak (nm)	RF	0.87		0.7	24
65	Experiment	Mn^4+^	Lifetime (ms)	RF			0.432	25
2832	DFT	Ce^3+^	Relative permittivity (ϵ_r_) (eV)	XGB	0.93		0.65	26
219	Experiment	Ce^3+^	Centroid shift (eV)	XGB	0.90	0.18		
76	Experiment	Ce^3+^	Emission peak (nm)	Kernel Ridge	0.79		12.64	27
			Thermal quenching (K)		0.64		37	
2610	DFT	Eu^2+^ and Ce^3+^	Debye temperature (K)	SVR	0.89	59.9	37.9	28
269	Experiment	Eu^3+^	Thermal quenching (K)	SVR	0.71		31	29
129	Experiment	Eu^2+^	Emission peak (nm)	XGB	0.78	42		30
1665	Experiment	Eu^2+^ and Eu^3+^	Emission peak (nm)	XGB	0.866		11.2	31
877			1st excitation max (nm)		0.775		8.83	
951			Decay time (ns)		0.987		0.09	
1252			CIE X coordinate		0.937		0.02	
1252			CIE Y coordinate		0.814		0.02	
183			Thermal quenching (K)		0.574		44.61	
555			Internal quantum efficiency		0.674		9.8	
56			External quantum efficiency		0.675		8.48	
186	Experiment	Cr^3+^	Emission peak (nm)	SVR	0.821		8.761	32
				KNN	0.85		9.125	
95	Experiment	Eu^2+^	Excitation wavelength (nm)	CBP	0.999	1.68		33
				Multiple linear	0.999	1.74		
				ANN	0.9999	1.83		
296	Experiment	Eu^3+^	Asymmetry ratio (*Λ*)	RF	0.90	1.03	0.77	34

### 3.1. Predicting the properties of Mn-doped phosphors

**Fig. 2:**
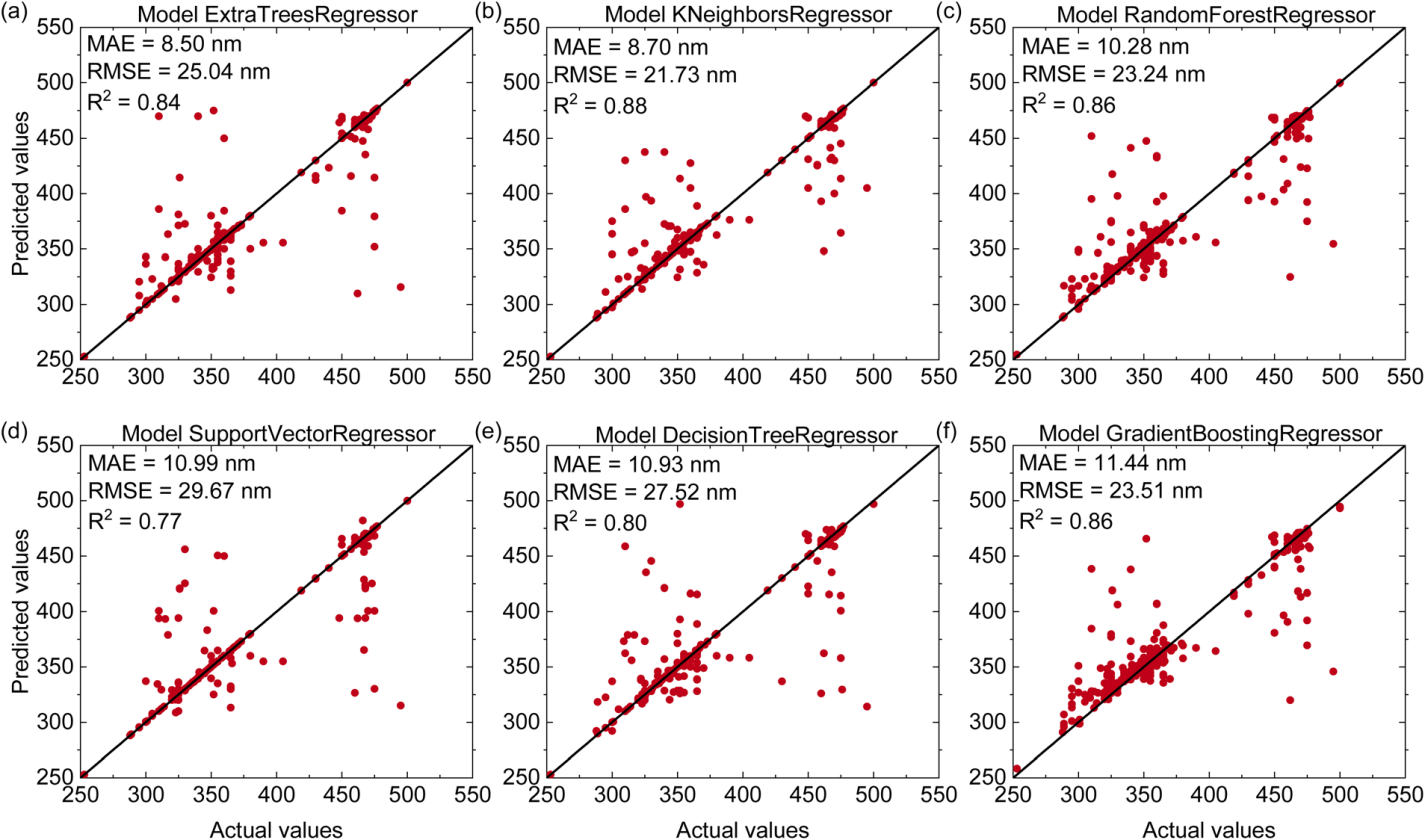
(Color online) Predictive performance of six regression models for excitation wavelength estimation of Mn^4+^-doped phosphors on the test dataset including (a) Extra Trees Regressor, (b) K-Nearest Neighbors Regressor, (c) Random Forest Regressor, (d) Support Vector Regressor, (e) Decision Tree Regressor, (f) Gradient Boosting Regressor.

Fig. 2 shows the predictive performance of six machine learning models for estimating the excitation wavelengths of Mn^4+^-doped phosphors. Among these models, the K-Nearest Neighbors Regressor achieves the best performance with an *R*^2^ of 0.88, an MAE of 8.70 nm, and an RMSE of 21.73 nm on the test set. The remaining models present lower accuracy with *R*^2^ values between 0.77 and 0.86, RMSE values from 23.24 to 29.67 nm, and MAE values from 8.5 to 11.44 nm. These results indicate that KNN captures the relationship between phosphor composition and excitation wavelength more effectively than other models. However, as seen in Fig. 2, there is a smaller subset of samples having noticeably larger deviations. These outliers show that some key factors are not captured by our composition-only representation. In reality, excitation wavelengths are strongly influenced by local coordination geometry, site symmetry, charge-compensation defects, possible multi-centre emission, and experimental issues such as overlapping excitation bands. Because these effects are not explicitly included in our descriptors, these outliers reveal the limits of a purely composition-based model and point to the need for future extensions.

Compared with previous work on Eu-doped phosphors (ref. 31), our models achieve higher *R*^2^ values, which are typically below 0.8 in earlier studies. In contrast, ref. 33 reported *R*^2^ values close to 1 because the dataset is small, less noisy, and based on simple luminescent materials with features strongly related to the predicted property. The evaluation was mainly performed on the training set with a small test set and without rigorous cross-validation. In our case, the Mn^4+^ dataset is much larger and chemically more diverse, so some residual scatter and a limited number of outliers are unavoidable in a minimal-input model. These outliers indicate that additional factors beyond chemical composition such as local structure, defects, or experimental uncertainties also influence the excitation behavior. We therefore view the present results as a realistic baseline for composition-only predictions and as a starting point for future models that incorporate more detailed structural descriptors.

**Fig. 3:**
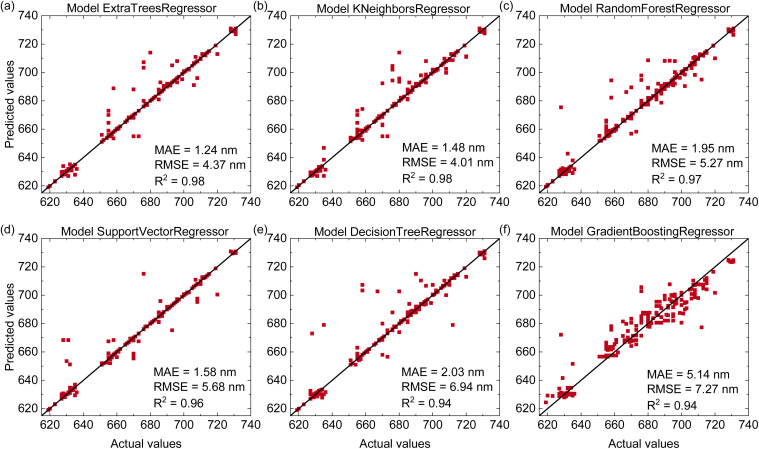
(Color online) Predictive performance of six regression models for emission-peak estimation of Mn^4+^-doped phosphors on the test dataset including (a) Extra Trees Regressor, (b) K-Nearest Neighbors Regressor, (c) Random Forest Regressor, (d) Support Vector Regressor, (e) Decision Tree Regressor, (f) Gradient Boosting Regressor.

The emission-wavelength prediction accuracy of six machine learning models for Mn^4+^-doped phosphors is compared in Fig. 3. All models show high accuracy with *R*^2^ values between 0.94 and 0.98, MAE values ranging from 1.24 to 5.14 nm, and RMSE values from 4.01 to 7.27 nm. Among them, the Extra Trees Regressor exhibits the best performance with the highest *R*^2^ of 0.98, the lowest MAE of 1.24 nm, and RMSE of 4.37 nm. The K-Nearest Neighbors, Random Forest, and Support Vector Regression models also provide good predictions with *R*^2^ values above 0.96. Compared with emission peak predictions in earlier studies^23,24,27,30–32^ (Table 1), our models achieve better accuracy due to the larger and more comprehensive dataset and the use of advanced algorithms. The maximum emission wavelengths of Mn^4+^-doped phosphors typically fall within two ranges: red (620–640 nm) and deep-red/far-red (650–740 nm). The Extra Trees Regressor is the most accurate in the red region, while the Support Vector Regressor performs slightly better for deep-red and far-red emissions. These results suggest that different algorithms capture distinct composition–property relationships, and that ensemble-based methods, particularly Extra Trees, are highly effective for modeling emission behavior.

To gain chemical insight into these predictions, we analyze the feature importance of the Extra Trees model for emission prediction (Fig. S4 in the SI). The analysis shows that fluorine (F), oxygen (O), lanthanum (La), and aluminum (Al) have the highest importance scores, while the remaining elements contribute more weakly. This trend is consistent with physical expectations. The presence of F and O anions significantly affects the local anion environment around Mn^4+^ and therefore have a strong influence on the crystal-field strength, covalency, and nephelauxetic effect.^37^ La and Al act as common host cations that control the local coordination geometry and lattice rigidity.^38,39^ By contrast, many other cations mainly play secondary structural or charge-balancing roles. As a result, they contribute less independent information to the model and thus receive lower feature-importance scores.

**Fig. 4:**
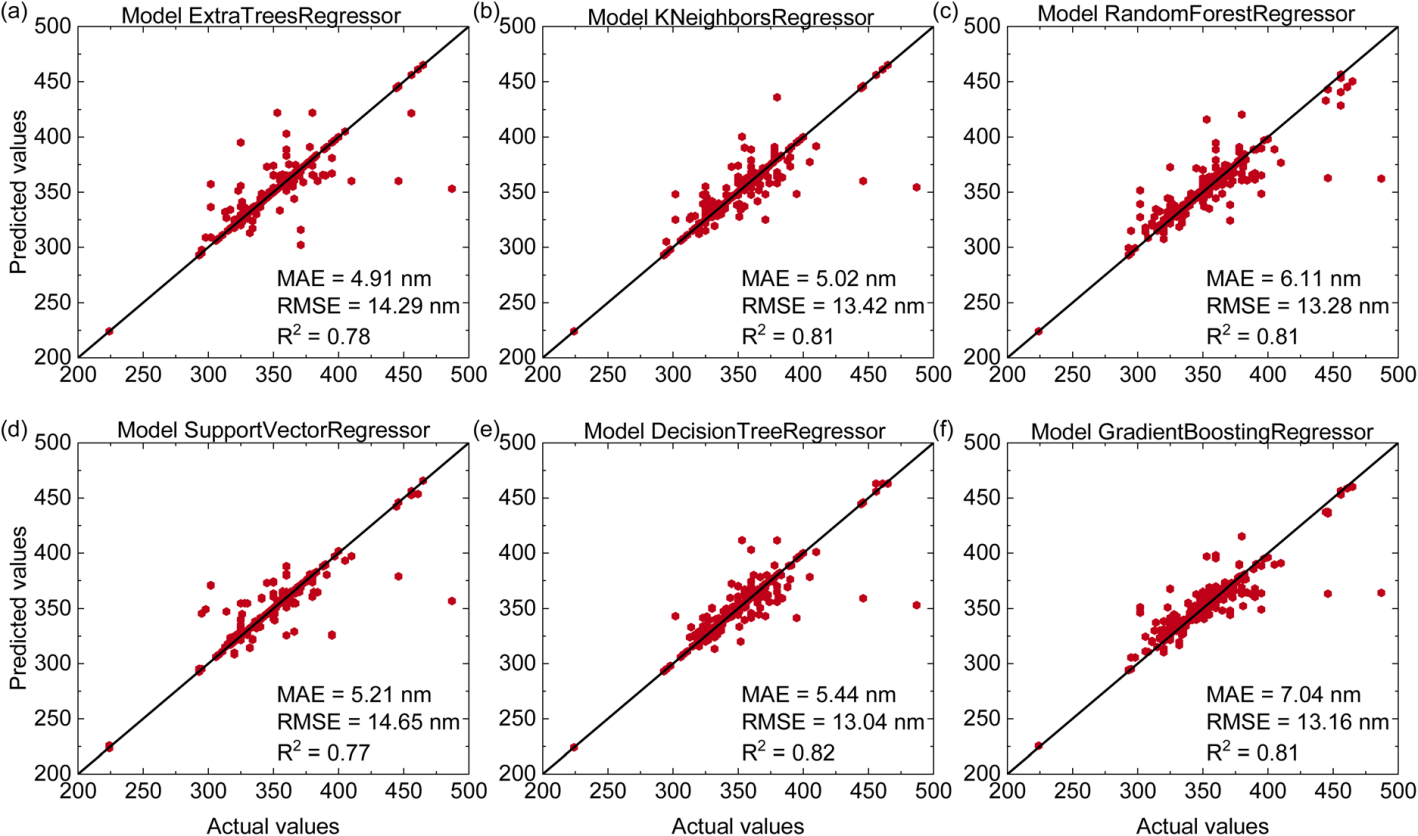
(Color online) Predictive performance of six regression models for estimating the ^4^T_1_ wavelength of Mn^4+^-doped phosphors on the test dataset including (a) Extra Trees Regressor, (b) K-Nearest Neighbors Regressor, (c) Random Forest Regressor, (d) Support Vector Regressor, (e) Decision Tree Regressor, (f) Gradient Boosting Regressor.

The predictive performance of six machine learning models for estimating the ^4^T_1_ energy levels of Mn^4+^-doped phosphors is shown in Fig. 4. The Decision Tree Regressor achieves the highest accuracy with an *R*^2^ of 0.82, an MAE of 5.44 nm, and an RMSE of 13.04 nm. The remaining models (Extra Trees, Support Vector Regression, K-Nearest Neighbors, Gradient Boosting Regressor and Random Forest) provide slightly lower predictive performance with *R*^2^ values ranging from 0.77 to 0.81. Compared with the emission-peak prediction, the accuracy for ^4^T_1_ is clearly reduced. This difference arises mainly from the way ^4^T_1_ energies are determined and from their stronger dependence on local structure. The ^4^A_2_ → ^4^T_1_ transition and other electronic transitions such as the charge-transfer band and the ^4^A_2_ → ^2^T_2_ transition can spectrally overlap. This leads to the experimental determination of ^4^T_1_ energies less precise and introduces uncertainties into the training dataset. In addition, the ^4^T_1_ energy is highly sensitive to local coordination geometry, crystal-field distortions, and covalency, whereas our descriptors do not fully capture these local effects.

**Fig. 5:**
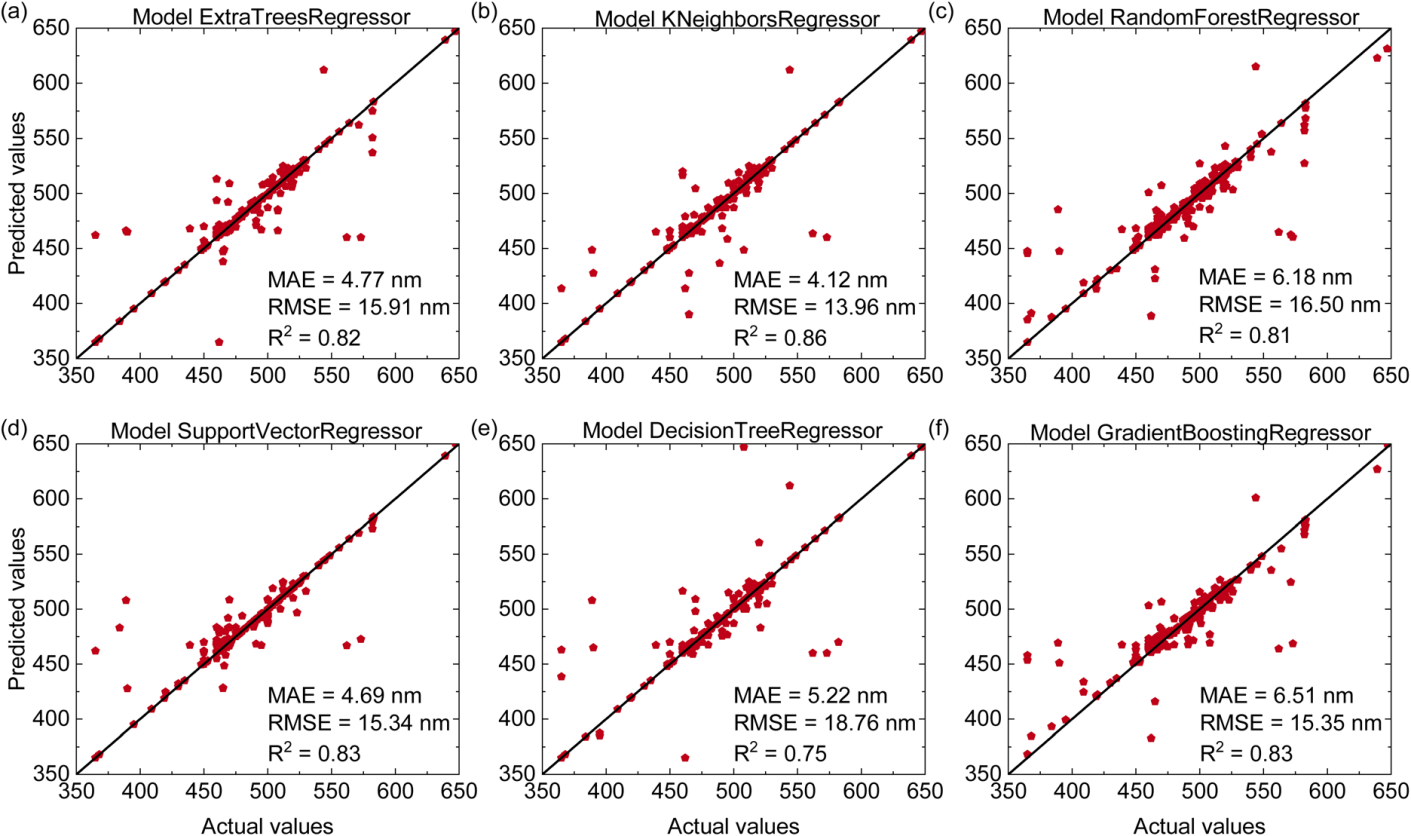
(Color online) Predictive performance of six regression models for estimating the ^4^T_2_ energy levels of Mn^4+^-doped phosphors on the test dataset including (a) Extra Trees Regressor, (b) K-Nearest Neighbors Regressor, (c) Random Forest Regressor, (d) Support Vector Regressor, (e) Decision Tree Regressor, (f) Gradient Boosting Regressor.

Fig. 5 shows the predictive performance of the six machine learning models for the ^4^T_2_ energy level. Unlike the results obtained for the ^4^T_1_ energy level, the K-Nearest Neighbors Regressor outperforms other models with an *R*^2^ of 0.86, MAE of 4.12 nm, and RMSE of 13.96 nm. The Decision Tree Regressor, which previously provided the best results for predicting ^4^T_1_ energies, shows significantly lower accuracy for the ^4^T_2_ level with an *R*^2^ of 0.75, MAE of 5.22 nm, and RMSE of 18.76 nm. The remaining models present intermediate predictive performance with *R*^2^ values ranging from 0.81 to 0.83. These findings suggest that different electronic transitions exhibit distinct relationships with compositional features and machine-learning models may be particularly effective at modeling the ^4^T_2_ energy level.

To further examine the generalization capability of the emission model, we applied the trained Extra Trees regressor to an independent set of Mn^4+^-doped phosphors that were not used in either training or testing. Specifically, we considered all Mn^4+^-activated compositions in very recent works^40–47^ that (i) exhibit a dominant red emission band and (ii) contain only elements represented in our descriptor space. As shown in Table 2, the absolute differences between predicted and experimental wavelengths range from 1.1 to 26.2 nm, with a mean deviation of approximately 10.7 nm and an RMSE of about 13.2 nm. These errors are larger than the internal test-set, as expected for an external validation set comprising newly reported materials. But these findings indicate that the model is able to provide reasonably accurate first-order estimates of emission peaks for previously unseen Mn^4+^-doped phosphors. Rather than serving as an exact line-position predictor, the current model is therefore best viewed as a screening tool to identify promising candidate compositions in the desired spectral range.

**Table 2:** Comparison of emission peak wavelengths (nm) predicted by the Extra Trees regression model with experimental values from recent studies (published in 2025) on Mn^4+^-doped phosphors

Formula	Actual	Predicted	Ref.
LaMg_3_Sb_0.999_O_7_Mn_0.001_	695	696.81	40
CaYMgNb_0.997_O_6_Mn_0.003_	688	691.82	41
CaAl_2_Si_1.992_O_8_Mn_0.1_	680	690.9	42
Mg_28_Ge_6.4_Sn_1.1_O_32_F_15.04_Mn_0.05_	659	638.37	43
Ca_0.8_Na_0.6_Gd_0.6_MgWO_6_Mn_0.0005_	685	698.2	44
La_3_Ga_5_Si_0.9998_O_14_Mn_0.0001_	713	686.8	45
CsNaWO_2_F_4_Mn_0.01_	631	622.55	46
Ca_1.99_Mn_0.01_La_3_Sb_3_O_14_	709	702.6	47
Zn_1.99_Mn_0.01_La_3_Sb_3_O_14_	690	704.75	47
Mg_1.99_Mn_0.01_La_3_Sb_3_O_14_	705	703.87	47

### 3.2. Predicting the properties of Eu-doped phosphors

To examine whether a composition-based representation can capture host-dependent trends that extend beyond a single activator ion, we next performed a transferability test on Eu-doped phosphors. Although Mn^4+^ and Eu^3+^ differ in their electronic configurations and detailed emission mechanisms, the positions of their emission bands are physically governed by the host lattice. These host effects are encoded in the elemental composition. Therefore, we apply the same composition-based modeling approach, originally developed for Mn^4+^-activated phosphors, to an independent Eu^3+^-doped dataset. This allows us to evaluate whether the approach remains effective across different activator ions.

**Fig. 6:**
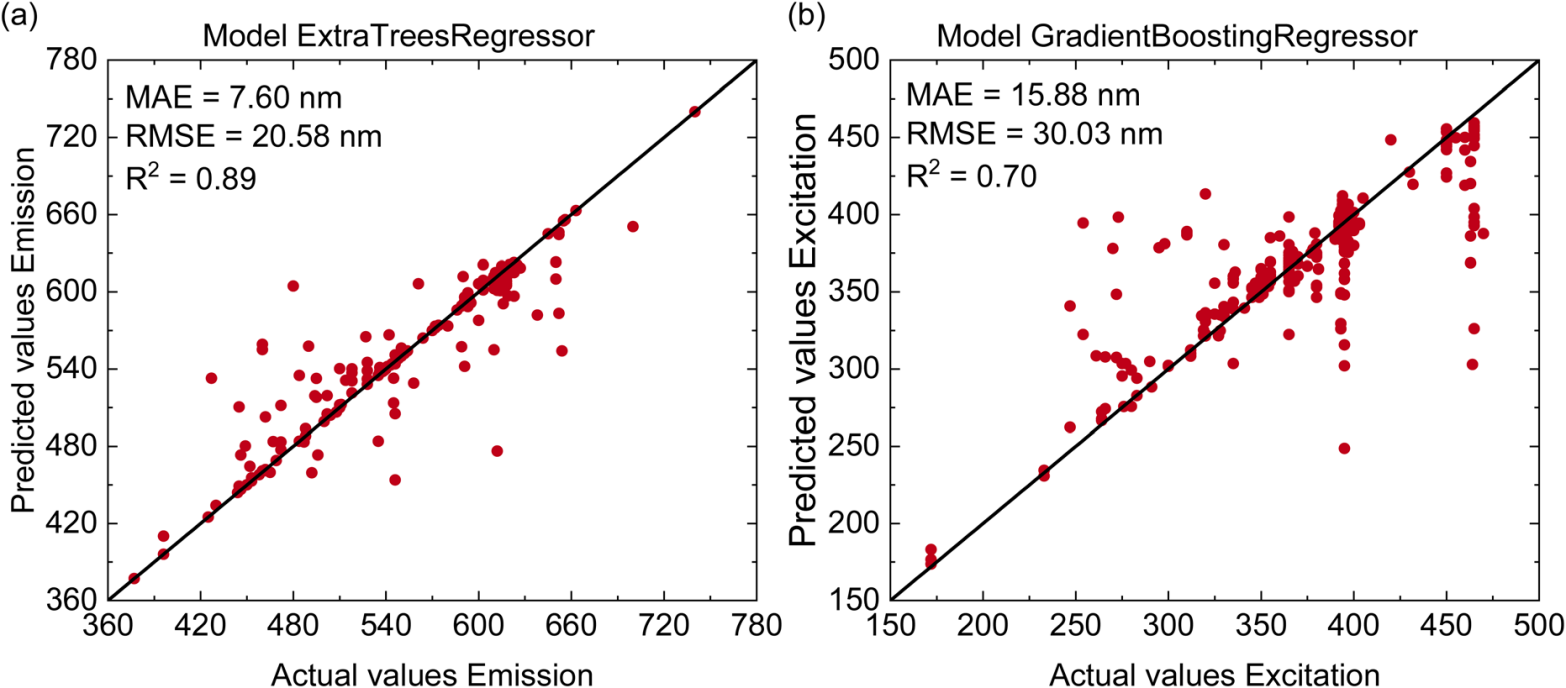
(Color online) Evaluation of prediction performance for (a) emission peak wavelength using the Extra Trees Regressor and (b) excitation wavelength using the Gradient Boosting Regressor on the test dataset for Eu-doped phosphors.

After training the machine learning models on Mn^4+^-doped phosphor data, we evaluate their transferability by applying them to a dataset of Eu-doped phosphors. The experimental dataset was obtained from a recent work of Jang,^31^ and the results are presented in Fig. 6. As shown in Fig. 6a, the Extra Trees Regressor predicts the emission peaks with relatively high accuracy, reaching *R*^2^ = 0.89, MAE = 7.6 nm, and RMSE = 20.58 nm. It is important to note that the model was trained only on Mn^4+^-doped phosphors, which emit in the 620–740 nm range, yet it is able to provide reasonably accurate predictions for a broader spectral range of 360–780 nm. In contrast, Fig. 6b presents the excitation wavelength prediction using the Gradient Boosting Regressor, which obtains an *R*^2^ of 0.7, an MAE of 30.03 nm, and an RMSE of 15.88 nm. These results indicate that the cross-dopant excitation predictions are less accurate than the emission predictions.

Compared with the results reported by Jang *et al.*,^31^ where emission-peak wavelength prediction achieved *R*^2^ = 0.866 and excitation prediction for the first peak reached *R*^2^ = 0.775, our model performs competitively or better. Jang's excitation model was trained only on the first excitation peak, while our model was trained on a broader dataset. For a fair comparison, we also retrain our model using only the first excitation peak data and obtain *R*^2^ = 0.88. Details of this analysis are provided in the SI. In addition to the validation on Eu-doped phosphors, our approach also shows superior performance on the Mn^4+^-doped dataset, where the best emission model reaches *R*^2^ = 0.98. This indicates a significant improvement over previous models, which reported *R*^2^ values between 0.78 and 0.87.^23,24,27,30–32^

To further validate the generalizability of our model, we compare its predictions with experimental data from several recent studies on Eu^3+^-doped phosphors published in 2025. Table 3 presents a direct comparison between predicted and experimental emission peaks for a series of compositions not included in the training and testing process. Across these 13 samples, the mean absolute deviation between predicted and experimental values is on the order of 10–20 nm. The Extra Trees model therefore remains reasonable predictive accuracy for Eu^3+^-doped systems, even though it was trained exclusively on Mn^4+^-doped data. Using very recent experimental data provides an independent check on model performance and indicates that the proposed framework can be applied to newly reported luminescent materials that were not part of the original training set.

**Table 3:** Comparison of emission peak wavelengths (nm) predicted by the Extra Trees regression model with experimental values reported in previous studies of Eu^3+^-doped phosphors

Formula	Actual	Predicted	Ref.
Sr_3_CaNb_1.994_O_9_Eu_0.06_	613	613.3	48
Ca_2_MgWO_6_Eu_0.01_Eu_0.02_	616	613.64	49
Y_4_Al_2_O_9_Eu_0.05_	611	595.45	50
La_2_LiNbO_6_Eu_0.2_	613	614.3	51
LiZnPO_4_Eu_0.03_	594	572.57	52
LiSnPO_4_Eu_0.03_	616	556.17	52
Sr_4_La_6_Si_6_O_24_Cl_2_Eu_0.1_	614	579.35	53
LaNb_2_VO_9_Eu_0.003_	618	614.95	54
SrLaZnNbO_6_Eu_0.11_	618	613.5	55
Ca_2_LaNbO_6_Eu_0.01_	615	614.15	56
Ca_3_Zr_2_SiGa_2_O_12_Eu_0.1_	610	572.15	57
Na_2_ZrO_3_Eu_0.002_	613	602.57	58
Sr_3_La_2_W_2_O_12_Eu_0.09_	616	608.82	59

### 3.3. Inverse design

In the final stage of this work, we propose a simple inverse-design approach. Since the Extra Trees Regressor model has been found to have the highest predictive performance for emission wavelength prediction, we choose this model to construct the inverse design framework. The inverse-design calculations use the same datasets as the forward case but the roles of inputs and outputs are reversed. Particularly, the excitation and emission wavelengths are taken as predictors, and the compositional vectors are used as targets. The continuous predictions are then converted into chemical formulas by rounding the atomic fractions to the nearest meaningful values. A predicted composition is regarded as correctly recovered when the reconstructed formula exactly matches the reported experimental one. The continuous predictions are then converted into chemical formulas by rounding the atomic fractions to the nearest meaningful values, and a predicted composition is regarded correctly recovered when the reconstructed formula exactly matches the reported experimental one. To the best of our knowledge, applying a tree-ensemble regressor in this composition-based inverse direction has not previously been proposed for investigating materials.

Our inverse-design approach is then applied to two datasets, one for Mn^4+^-doped phosphors and one for Eu-doped phosphors. The results show that on the Mn^4+^ dataset, the model successfully predicts the compositions of 265 out of 347 test samples. Similarly, on the Eu dataset, it correctly identifies 144 out of 333 compositions in the test set. Because the Extra Trees regressor is an unconstrained continuous model, its outputs for the atomic fractions are real numbers and are not mathematically forced to satisfy compositional constraints. In principle, the predicted fractions may sum to slightly more or less than 100% or even become negative. In our calculations, we do not observe negative fractions. To obtain chemically meaningful formulas, we therefore discard any predicted composition with a total atomic fraction that deviates from 100%. This screening is applied only to the model outputs in the inverse-design stage. All input compositions in the training and test sets are taken directly from experiment and are already physically valid. Under this constraint, about 96% of the suggested compositions remain valid. This indicates that our inverse-design scheme can propose phosphor compositions from desired optical targets. Rather than serving as a purely generative model, it provides a practical tool to rapidly screen and suggest new phosphor candidates. Thereby, our calculations support experimental synthesis and reduce the time and resources required for materials discovery. The predicted compositions generated by the inverse-design model are listed in SII for Mn^4+^-doped phosphors and SIII for Eu-doped phosphors in the SI.

Our inverse-design calculations are carried out purely in composition space under simple chemical constraints. All atomic fractions are non-negative, renormalized to sum to 100%, and the Mn or Eu dopant content is limited to the experimental range. The present reverse-engineering scheme operates only at the composition level and is consequently more limited than structure-aware inverse-design approaches that explicitly optimize lattice or microstructural degrees of freedom. However, such structure-resolved methods require reliable crystal structure models and high-cost atomistic calculations. This causes their systematic application to thousands of candidate phosphors to be challenging. Consequently, our inverse-design model is intended as a fast-screening tool that can guide subsequent structure-based simulations or experimental validation.

## 4. Conclusion

In conclusion, we have developed a composition-based machine learning framework for predicting key optical properties of Mn^4+^-doped phosphors including the excitation wavelength, emission peak, and ^4^T_1_ and ^4^T_2_ transition wavelengths. For the first time, we collected the largest experimental dataset of Mn^4+^-doped phosphors to train and evaluate multiple machine-learning models. Among these models, the K-Nearest Neighbors and Extra Trees Regressors provided the best predictive performance for excitation and emission wavelengths, respectively. The trained models were further validated on Eu^3+^-doped phosphors to present promising transferability across different dopant systems. An inverse design approach was also developed to generate candidate phosphor compositions based on user-defined optical targets. We note that all Mn-doped compositions in our dataset have nominal Mn contents below 10%, so the predictive performance reported here is valid for the 0–10% Mn-doping range and should not be extrapolated to higher concentrations without additional data. Within the present Mn^4+^ dataset, we do not observe any specific host family or compositional class where the model consistently fails. Prediction errors are distributed across different hosts. Comparable accuracies are also obtained for Eu^3+^-doped phosphors and in our other phosphor studies (under study and not shown here). These observations indicate that the composition-based approach works reliably across a broad range of chemistries, while still leaving room for future structure-informed models.

This work directly addresses the research questions raised in the Introduction. We showed that accurate predictions of the excitation wavelength, emission peak, and ^4^T_1_ and ^4^T_2_ transition wavelengths can be determined using only elemental composition without requiring experimental properties or complex descriptors. We further show that models trained solely on Mn^4+^-doped phosphors can generalize to Eu^3+^-doped systems, highlighting their transferability across different dopant types. Additionally, our models can be optimized for specific spectral regions and integrated into an inverse design to propose candidate compositions that meet desired optical targets. Compared with previous studies, our approach exhibits higher predictive accuracy while requiring only simple compositional input. Our study provides a minimal-input and data-driven approach for accelerating the discovery and design of high-performance phosphors and expanding the search space for next-generation luminescent materials.

## Conflicts of interest

The authors have no conflicts to disclose.

## Supplementary Material

RA-016-D6RA00029K-s001

RA-016-D6RA00029K-s002

RA-016-D6RA00029K-s003

## Data Availability

The source code used in this study can be found at Github with https://github.com/NgoQue/MLPhosphors. Supplementary information (SI) is available. See DOI: https://doi.org/10.1039/d6ra00029k.

## References

[cit1] Blasse G. and Grabmaier B. C., Luminescent Materials, Springer-Verlag, Berlin, Germany, 1994

[cit2] Rahman J. U., Khan S., Jain V., Rajiv A., Dasi S., Fawy K. F., Jindal P. K., Sivaranjani R. (2025). Rev. Inorg. Chem..

[cit3] Zhao M., Zhang Q., Xia Z. (2020). Mater. Today.

[cit4] Chen H.-W., Lee J.-H., Lin B.-Y. (2018). et al.. Light: Sci. Appl..

[cit5] Wang X., Liu Q., Bu Y., Liu C.-S., Liu T., Yan X. (2015). RSC Adv..

[cit6] Crawford S. E., Ohodnicki P. R., Baltrus J. P. (2020). J. Mater. Chem. C.

[cit7] Choi J. R., Yong K. W., Choi J. Y., Nilghaz A., Lin Y., Xu J., Lu X. (2018). Theranostics.

[cit8] Wang C., Wang X., Zhou Y., Zhang S., Li C., Hu D., Xu L., Jiao H. (2019). ACS Appl. Electron. Mater..

[cit9] Shin H. G., Timilsina S., Sohn K.-S., Kim J. S. (2022). Adv. Sci..

[cit10] Qian X., Cai Z., Su M., Li F., Fang W., Li Y., Zhou X., Li Q., Feng X., Li W. (2018). et al.. Adv. Mater..

[cit11] Fang M.-H., Bao Z., Huang W.-T., Liu R.-S. (2022). Chem. Rev..

[cit12] Hariyani S., Sójka M., Setlur A. (2023). et al.. Nat. Rev. Mater..

[cit13] Zhou X., Qiao J., Xia Z. (2021). Chem. Mater..

[cit14] Sugano S. , Tanabe Y., and Kamimura H., Multiplets of Transition-Metal Ions in Crystals, Academic Press, New York, 1970

[cit15] Zhou Y., Ma Q., Lu M., Qiu Z., Zhang A. (2008). J. Phys. Chem. C.

[cit16] Yadav R. S., Rai S. B. (2019). Opt. Laser Technol..

[cit17] Pelant I. and Valenta J., Luminescence Spectroscopy of Semiconductors, Oxford University Press, Oxford, 2012

[cit18] Lakowicz J. R. , Principles of Fluorescence Spectroscopy, Springer, 2006

[cit19] Gfroerer T. H. (2000). et al.. Encycl. Anal. Chem..

[cit20] Que N. T., Phan A. D., Tran T., Huy P. T., Trang M. X., Luong T. V. (2025). Mater. Today Commun..

[cit21] Phan A. D., Que N. T., Nguyen Duyen T. T., Thanh Viet P., Quach Q. K., Mei B. (2025). J. Appl. Phys..

[cit22] Novita M., Chauhan A. S., Ujianti R. M. D., Marlina D., Kusumo H., Anwar M.
T., Piasecki M., Brik M. G. (2024). J. Lumin..

[cit23] Ding C., Li Z., Zhang W., Ou J., Wen X., Xin C., Su M. (2023). New J. Chem..

[cit24] Wang Y., Tang W., Zhang C., Molokeev M. S., Ming H., Zhou Y., Peng S., Song E., Zhang Q. (2024). Adv. Funct. Mater..

[cit25] Ming H., Zhou Y., Molokeev M. S., Zhang C., Huang L., Wang Y., Sun H.-T., Song E., Zhang Q. (2024). ACS Mater. Lett..

[cit26] Zhuo Y., Hariyani S., You S., Dorenbos P., Brgoch J. (2020). J. Appl. Phys..

[cit27] Jiang L., Jiang X., Zhang Y., Wang C., Liu P., Lv G., Su Y. (2022). ACS Appl. Mater. Interfaces.

[cit28] Zhuo Y., Tehrani A. M., Oliynyk A. O., Duke A. C., Brgoch J. (2018). Nat. Commun..

[cit29] Zhuo Y., Hariyani S., Armijo E., Lawson Z. A., Brgoch J. (2019). ACS Appl. Mater. Interfaces.

[cit30] Koyama Y., Ikeno H., Harada M., Funahashi S., Takeda T., Hirosaki N. (2023). Mater. Adv..

[cit31] Jang S., Na G. S., Choi Y., Chang H. (2024). Sci. Rep..

[cit32] Xu W., Wang R., Hu C., Wen G., Cui J., Zheng L., Sun Z., Zhang Y., Zhang Z. (2024). npj Comput. Mater..

[cit33] Sahu S. K., Shrivastav A., Swamy N. K., Dubey V., Halwar D. K., Kumar M. T., Rao M. C. (2024). J. Appl. Spectrosc..

[cit34] Otsuka T., Oka R., Karasuyama M., Hayakawa T. (2024). Phys. Status Solidi RRL.

[cit35] https://scikit-learn.org/stable/

[cit36] https://scikit-learn.org/stable/modules/generated/sklearn.model_selection.GridSearchCV.html

[cit37] Kim Y.-I., Woodward P. M. (2019). Catalysts.

[cit38] Chen Y., Yang C., Deng M., He J., Xu Y., Liu Z.-Q. (2019). Dalton Trans..

[cit39] Lu W., Lv W., Zhao Q., Jiao M., Shao B., You H. (2014). Inorg. Chem..

[cit40] Wang F., Chen H. (2025). J. Alloy. Compd..

[cit41] Liao C., Zhao W., Xiang Y., Zhu S., Huang X., Chen Z., Xu J., Jiang C., Wu M., Zhong J. (2025). Mater. Today Chem..

[cit42] Chi F., Zhang J., Zheng Y., Niu X., Liu J., Zhang X., Jiang B., Liu S., Wei X. (2025). Ceram. Int..

[cit43] Li M., Wang L., Shi Q., Guo H., Qiao J., Han H., Cui C., Huang P. (2025). Ceram. Int..

[cit44] Li C., Kang R., Ma X., Xie J., Wang Y., Seto T. (2025). Small.

[cit45] Zhang N.-N., Wang H.-Y., Yan X.-Y., Wang X.-P., Liu B., Zhang Y.-Y., Yang Y.-G. (2025). J. Mol. Struct..

[cit46] Fang W., Yang Y., Liu Y., Ma D., Huang J., Song B., Xia L. (2025). Inorg. Chem..

[cit47] Kai H.-Y., Wong K.-L., Tanner P. A. (2025). Next Mater..

[cit48] Khan N. Z., Khan S. A., Muhammad N., Chen W., Ahmed J., Padhiar M. A., Chen M., Runowski M., Alshehri S. M., Zhang B. (2025). Adv. Opt. Mater..

[cit49] Kiran R., Kennedy S. M. M., Princy A., Sayyed M. I., Almuqrin A. H., Kamath S. D. (2025). J. Photochem. Photobiol. A Chem..

[cit50] Arunakumar R., Gagana M., Radha Krushna B. R., Pruthviraj I. S., Ramakrishna G., Sharma S. C., Choudhury S. P. N., Shanma E., Kumari B. N., Manjunatha K. (2025). et al.. J. Lumin..

[cit51] Cao B., Lu Y., Zhang T., Wu H., Li Y., Deng C., Huang W. (2025). J. Mol. Struct..

[cit52] İlhan M. İ., Güleryüz L. F., Katı M. İ. (2025). Mater. Sci. Eng. B.

[cit53] Wang Y., Shen M., Zheng H., Lu Y., Du P. (2025). Adv. Opt. Mater..

[cit54] Du Y., Zhao H., Guo T., Xu Z., Qing R., Jabeen S., Che J., Tong S., Du X., Yu R. (2025). J. Photochem. Photobiol. A Chem..

[cit55] Kumar M. N., Samuel P. (2025). Ceram. Int..

[cit56] Liu S., Zhong L., Xiang Y., Chen Z., Xie M., Hong J., Zhou L., Wu M. (2025). Mater. Today Chem..

[cit57] Chen J., Chen Y., Guo H. (2025). J. Alloy. Compd..

[cit58] Khajuria P., Sharma V. D., Kumar I., Khajuria A., Prakash R., Choudhary R. J. (2025). J. Alloys Compd..

[cit59] Wang F., Chen H., Zhang S., Jin H. (2025). J. Am. Ceram. Soc..

