# Supplementary material for: Composition-based machine learning for predicting and designing Mn4+-doped phosphors
Source: RSC Adv. 2026 Feb 27;16(13):11415–25. doi: 10.1039/d6ra00029k (PMC12947634; doi:10.1039/d6ra00029k)
Supplement: RA-016-D6RA00029K-s001 [file RA-016-D6RA00029K-s001.pdf]

This journal is © The Royal Society of Chemistry 2026

Formula	Ref_link
K2Ti0.992F6Mn0.008	https://doi.org/10.1038/s41377-018-0013-1
K2Ti0.999F6Mn0.001	https://doi.org/10.1038/s41377-018-0013-1
K2Ti0.843F6Mn0.157	https://doi.org/10.1038/s41377-018-0013-1
K2Ti0.843F6Mn0.157	https://doi.org/10.1038/s41377-018-0013-1
Mg2Ti0.9999O4Mn0.0001	https://doi.org/10.1039/C3TC30553H
Mg2Ti0.9995O4Mn0.0005	https://doi.org/10.1039/C3TC30553H
Mg2Ti0.999O4Mn0.001	https://doi.org/10.1039/C3TC30553H
Mg2Ti0.995O4Mn0.0015	https://doi.org/10.1039/C3TC30553H
LaMgAl10.995O19Mn0.005	https://doi.org/10.3390/ma12010086
LaMgAl10.99O19Mn0.01	https://doi.org/10.3390/ma12010086
LaMgAl10.95O19Mn0.05	https://doi.org/10.3390/ma12010086
Sr2MgAl21.9995O36Mn0.0005	http://dx.doi.org/10.1149/2.022204jss
Sr2MgAl21.999O36Mn0.001	http://dx.doi.org/10.1149/2.022204jss
Sr2MgAl21.997O36Mn0.003	http://dx.doi.org/10.1149/2.022204jss
Sr2MgAl21.994O36Mn0.006	http://dx.doi.org/10.1149/2.022204jss
Sr2MgAl21.991O36Mn0.009	http://dx.doi.org/10.1149/2.022204jss
Sr2MgAl21.988O36Mn0.012	http://dx.doi.org/10.1149/2.022204jss
Sr2MgAl21.985O36Mn0.015	http://dx.doi.org/10.1149/2.022204jss
Sr2MgAl21.982O36Mn0.018	http://dx.doi.org/10.1149/2.022204jss
Sr2MgAl21.979O36Mn0.021	http://dx.doi.org/10.1149/2.022204jss
Sr2MgAl21.976O36Mn0.024	http://dx.doi.org/10.1149/2.022204jss
Mg1Al11.995O19Mn0.005	https://doi.org/10.1364/OL.33.001816
Ca0.8Mg0.2Al11.995O19Mn0.005	https://doi.org/10.1364/OL.33.001816
Ca0.6Mg0.4Al11.995O19Mn0.005	https://doi.org/10.1364/OL.33.001816
Ca0.5Mg0.5Al11.995O19Mn0.005	https://doi.org/10.1364/OL.33.001816
Ca0.4Mg0.6Al11.995O19Mn0.005	https://doi.org/10.1364/OL.33.001816
Ca0.2Mg0.8Al11.995O19Mn0.005	https://doi.org/10.1364/OL.33.001816
Ca1Al11.995O19Mn0.005	https://doi.org/10.1364/OL.33.001816
BeAl1.997O4:Mn0.003	https://doi.org/10.1016/j.ceramint.2018.08.006
BaMg6Ti5.994O19Mn0.001	https://doi.org/10.1246/cl.140282

CsNaGe0.99F6Mn0.01	https://doi.org/10.1021/acs.inorgchem.8b02488
CsNaGe0.97F6Mn0.03	https://doi.org/10.1021/acs.inorgchem.8b02488
CsNaGe0.95F6Mn0.05	https://doi.org/10.1021/acs.inorgchem.8b02488
CsNaGe0.93F6Mn0.07	https://doi.org/10.1021/acs.inorgchem.8b02488
CsNaGe0.91F6Mn0.09	https://doi.org/10.1021/acs.inorgchem.8b02488
CsNaGe0.89F6Mn0.11	https://doi.org/10.1021/acs.inorgchem.8b02488
CsNaGe0.87F6Mn0.13	https://doi.org/10.1021/acs.inorgchem.8b02488
Na2Ge0.99F6Mn0.01	https://doi.org/10.1021/acs.inorgchem.8b02488
Cs2Ge0.99F6Mn0.01	https://doi.org/10.1021/acs.inorgchem.8b02488
Na2Si0.995F6Mn0.005	https://doi.org/10.1039/C6QM00284F
Na2Si0.99F6Mn0.01	https://doi.org/10.1039/C6QM00284F
Na2Si0.985F6Mn0.015	https://doi.org/10.1039/C6QM00284F
Na2Si0.98F6Mn0.02	https://doi.org/10.1039/C6QM00284F
Na2Si0.97F6Mn0.03	https://doi.org/10.1039/C6QM00284F
Na2Si0.96F6Mn0.04	https://doi.org/10.1039/C6QM00284F
BaTi0.95F6Mn0.05	https://doi.org/10.1039/C6DT03398A
BaTi0.98F6Mn0.02	https://doi.org/10.1039/C6DT03398A
BaTi0.9F6Mn0.1	https://doi.org/10.1039/C6DT03398A
BaTi0.85F6Mn0.15	https://doi.org/10.1039/C6DT03398A
BaTi0.8F6Mn0.2	https://doi.org/10.1039/C6DT03398A
Na3Ga0.995F6Mn0.005	https://doi.org/10.1039/C7TC00011A
Na3Ga0.99F6Mn0.01	https://doi.org/10.1039/C7TC00011A
Na3Ga0.98F6Mn0.02	https://doi.org/10.1039/C7TC00011A
Na3Ga0.95F6Mn0.05	https://doi.org/10.1039/C7TC00011A
Na3Ga0.9F6Mn0.1	https://doi.org/10.1039/C7TC00011A
Li2Sn0.996O3Mn0.004	https://doi.org/10.1016/j.jallcom.2017.02.079
Li2Sn0.998O3Mn0.002	https://doi.org/10.1016/j.jallcom.2017.02.079
Li2Sn0.994O3Mn0.006	https://doi.org/10.1016/j.jallcom.2017.02.079
Li2Sn0.992O3Mn0.008	https://doi.org/10.1016/j.jallcom.2017.02.079
Li2Sn0.99O3Mn0.01	https://doi.org/10.1016/j.jallcom.2017.02.079
CaAl1.998O4Mn0.002	https://doi.org/10.1016/j.jallcom.2017.02.079

CaAl1.996O4Mn0.004	https://doi.org/10.1016/j.jallcom.2017.02.079
CaAl1.994O4Mn0.006	https://doi.org/10.1016/j.jallcom.2017.02.079
CaAl1.99O4Mn0.01	https://doi.org/10.1016/j.jallcom.2017.02.079
CaAl1.992O4Mn0.008	https://doi.org/10.1016/j.jallcom.2017.02.079
CaAl1.982O4Mn0.012	https://doi.org/10.1016/j.jallcom.2017.02.079
CaAl1.986O4Mn0.014	https://doi.org/10.1016/j.jallcom.2017.02.079
CaAl1.984O4Mn0.016	https://doi.org/10.1016/j.jallcom.2017.02.079
SrAl1.99O4Mn4+0.01	https://doi.org/10.1016/j.jallcom.2017.02.079
CaAl3.99O7Mn40.01	https://doi.org/10.1016/j.jallcom.2017.02.079
CaYAl0.999O4Mn0.001	https://doi.org/10.1186/s40539-014-0015-4
CaYAl0.995O4Mn0.005	https://doi.org/10.1186/s40539-014-0015-4
CaYAl0.99O4Mn0.01	https://doi.org/10.1186/s40539-014-0015-4
CaYAl0.97O4Mn0.03	https://doi.org/10.1186/s40539-014-0015-4
CaYAl0.95O4Mn0.05	https://doi.org/10.1186/s40539-014-0015-4
LaAl0.998O3Mn0.002	https://link.springer.com/article/10.1007/s00339-016-9860-x
LaAl0.996O3Mn0.004	https://link.springer.com/article/10.1007/s00339-016-9860-x
LaAl0.994O3Mn0.006	https://link.springer.com/article/10.1007/s00339-016-9860-x
LaAl0.992O3Mn0.008	https://link.springer.com/article/10.1007/s00339-016-9860-x
LaAl0.99O3Mn0.01	https://link.springer.com/article/10.1007/s00339-016-9860-x
LaAl0.988O3Mn0.012	https://link.springer.com/article/10.1007/s00339-016-9860-x
LaAl0.986O3Mn0.014	https://link.springer.com/article/10.1007/s00339-016-9860-x
LaAl0.984O3Mn0.016	https://link.springer.com/article/10.1007/s00339-016-9860-x
La0.992Na0.008Al0.992O3Mn0.008	https://link.springer.com/article/10.1007/s00339-016-9860-x
SrMgAl9.985O17Mn0.015	https://doi.org/10.1007/s10854-014-1928-9
SrMgAl9.9975O17Mn0.0025	https://doi.org/10.1007/s10854-014-1928-9
SrMgAl9.995O17Mn0.005	https://doi.org/10.1007/s10854-014-1928-9
SrMgAl9.99O17Mn0.01	https://doi.org/10.1007/s10854-014-1928-9
SrMgAl9.98O17Mn0.02	https://doi.org/10.1007/s10854-014-1928-9
SrMgAl9.975O17Mn0.025	https://doi.org/10.1007/s10854-014-1928-9
SrMgAl9.965O17Mn0.035	https://doi.org/10.1007/s10854-014-1928-9
KNaSi0.94F6Mn0.06	https://doi.org/10.1021/acsami.6b01905

LiGa0.994TiO4:Mn0.006	https://doi.org/10.1016/j.ceramint.2016.05.138
LiGa0.998TiO4:Mn0.002	https://doi.org/10.1016/j.ceramint.2016.05.138
LiGa0.996TiO4:Mn0.004	https://doi.org/10.1016/j.ceramint.2016.05.138
LiGa0.992TiO4:Mn0.008	https://doi.org/10.1016/j.ceramint.2016.05.138
LiGa0.99TiO4:Mn0.01	https://doi.org/10.1016/j.ceramint.2016.05.138
LiGa0.988TiO4:Mn0.012	https://doi.org/10.1016/j.ceramint.2016.05.138
LiGa0.998TiO4:Mn0.002	https://doi.org/10.1016/j.ceramint.2016.05.138
Ca14Zn6Al9.97O35Mn0.03	https://doi.org/10.1246/cl.140227
Ca14Zn6Ga9.97O35Mn0.04	https://doi.org/10.1246/cl.140227
Li3Mg2Nb0.996O6Mn0.004	https://doi.org/10.1016/j.optmat.2016.04.041
Li3Mg2Nb0.998O6Mn0.002	https://doi.org/10.1016/j.optmat.2016.04.041
Li3Mg2Nb0.994O6Mn0.006	https://doi.org/10.1016/j.optmat.2016.04.041
Li3Mg2Nb0.992O6Mn0.008	https://doi.org/10.1016/j.optmat.2016.04.041
Li3Mg2Nb0.99O6Mn0.010	https://doi.org/10.1016/j.optmat.2016.04.041
Li3Mg2Nb0.988O6Mn0.012	https://doi.org/10.1016/j.optmat.2016.04.041
La2LiTa0.997O6 :Mn0.003	https://doi.org/10.1007/s00339-014-8827-z
La2LiTa0.999O6 :Mn0.001	https://doi.org/10.1007/s00339-014-8827-z
La2LiTa0.995O6 :Mn0.005	https://doi.org/10.1007/s00339-014-8827-z
La2LiTa0.993O6 :Mn0.007	https://doi.org/10.1007/s00339-014-8827-z
La2LiTa0.99O6 :Mn0.01	https://doi.org/10.1007/s00339-014-8827-z
CaAl11.995O19:Mn0.005	https://doi.org/10.1016/j.ceramint.2017.01.105
CaAl11.999O19:Mn0.001	https://doi.org/10.1016/j.ceramint.2017.01.105
CaAl11.992O19:Mn0.008	https://doi.org/10.1016/j.ceramint.2017.01.105
CaAl11.99O19:Mn0.01	https://doi.org/10.1016/j.ceramint.2017.01.105
CaAl11.988O19:Mn0.012	https://doi.org/10.1016/j.ceramint.2017.01.105
CaAl11.985O19:Mn0.015	https://doi.org/10.1016/j.ceramint.2017.01.105
CaAl11.98O19:Mn0.02	https://doi.org/10.1016/j.ceramint.2017.01.105
Rb2Ge3.9998O9:Mn0.0002	https://doi.org/10.1111/jace.14363
Rb2Ge3.9996O9:Mn0.0004	https://doi.org/10.1111/jace.14363
Rb2Ge3.9992O9:Mn0.0008	https://doi.org/10.1111/jace.14363
Rb2Ge3.998O9:Mn0.002	https://doi.org/10.1111/jace.14363

Rb2Ge3.994O9:Mn0.006	https://doi.org/10.1111/jace.14363
Rb2Ge3.98O9:Mn0.02	https://doi.org/10.1111/jace.14363
Rb2Ge3.96O9:Mn0.04	https://doi.org/10.1111/jace.14363
Rb2Ge3.92O9:Mn0.08	https://doi.org/10.1111/jace.14363
K0.5Rb1.5Ge3.9992O9:Mn0.0008	https://doi.org/10.1111/jace.14363
K1Rb1Ge3.9992O9:Mn0.0008	https://doi.org/10.1111/jace.14363
K1.5Rb0.5Ge3.9992O9:Mn0.0008	https://doi.org/10.1111/jace.14363
K2Ge3.9992O9Mn0.0008	https://doi.org/10.1111/jace.14363
Mg2Ti0.9999O4Mn0.0001	https://doi.org/10.1016/0022-2313(74)90003-9
Mg2Ti0.9995O4Mn0.0005	https://doi.org/10.1016/0022-2313(74)90003-9
Mg2Ti0.999O4Mn0.001	https://doi.org/10.1016/0022-2313(74)90003-9
Mg2Ti0.995O4Mn0.005	https://doi.org/10.1016/0022-2313(74)90003-9
Mg2Ti0.99O4Mn0.01	https://doi.org/10.1016/0022-2313(74)90003-9
Mg2Ti0.985O4Mn0.015	https://doi.org/10.1016/0022-2313(74)90003-9
CaZr0.999O3Mn0.001	https://doi.org/10.1016/0022-1902(81)80325-9
Ca0Mg1Al11.995O19Mn0.005	https://doi.org/10.1016/j.jallcom.2010.11.117
Ca0.8Mg0.2Al11.995O19Mn0.005	https://doi.org/10.1016/j.jallcom.2010.11.117
Ca0.6Mg0.4Al11.995O19Mn0.005	https://doi.org/10.1016/j.jallcom.2010.11.117
Ca0.5Mg0.Al11.995O19Mn0.005	https://doi.org/10.1016/j.jallcom.2010.11.117
Ca0.4Mg0.6Al11.995O19Mn0.005	https://doi.org/10.1016/j.jallcom.2010.11.117
Ca0.2Mg0.8Al11.995O19Mn0.005	https://doi.org/10.1016/j.jallcom.2010.11.117
Ca1Mg0Al11.995O19Mn0.005	https://doi.org/10.1016/j.jallcom.2010.11.117
Ca2Mg2Al27.998O46Mn0.002	https://doi.org/10.1016/j.ceramint.2017.02.149
Ca2Mg2Al27.996O46Mn0.004	https://doi.org/10.1016/j.ceramint.2017.02.149
Ca2Mg2Al27.992O46Mn0.008	https://doi.org/10.1016/j.ceramint.2017.02.149
Ca2Mg2Al27.99O46Mn0.01	https://doi.org/10.1016/j.ceramint.2017.02.149
Ca2Mg2Al27.988O46Mn0.012	https://doi.org/10.1016/j.ceramint.2017.02.149
Ca2Mg2Al27.986O46Mn0.014	https://doi.org/10.1016/j.ceramint.2017.02.149
Ca2Mg2Al27.984O46Mn0.016	https://doi.org/10.1016/j.ceramint.2017.02.149
Ca2Mg2Al27.982O46Mn0.018	https://doi.org/10.1016/j.ceramint.2017.02.149
Ca2Mg2Al27.92O46Mn0.02	https://doi.org/10.1016/j.ceramint.2017.02.149

CaMg2Al15.998O27Mn0.002	https://doi.org/10.1016/j.ceramint.2017.02.149
CaMg2Al15.996O27Mn0.004	https://doi.org/10.1016/j.ceramint.2017.02.149
CaMg2Al15.992O27Mn0.008	https://doi.org/10.1016/j.ceramint.2017.02.149
CaMg2Al15.99O27Mn0.01	https://doi.org/10.1016/j.ceramint.2017.02.149
CaMg2Al15.988O27Mn0.012	https://doi.org/10.1016/j.ceramint.2017.02.149
CaMg2Al15.986O27Mn0.014	https://doi.org/10.1016/j.ceramint.2017.02.149
CaMg2Al15.984O27Mn0.016	https://doi.org/10.1016/j.ceramint.2017.02.149
CaMg2Al15.982O27Mn0.018	https://doi.org/10.1016/j.ceramint.2017.02.149
CaMg2Al15.92O27Mn0.02	https://doi.org/10.1016/j.ceramint.2017.02.149
Li2Ge3.998O9Mn0.002	https://doi.org/10.1016/j.jlumin.2017.08.036
LiNaGe3.998O9Mn0.002	https://doi.org/10.1016/j.jlumin.2017.08.036
K2Ge3.998O9Mn0.002	https://doi.org/10.1016/j.jlumin.2017.08.036
Ca14Al9.85Zn6O35Mn0.15	https://doi.org/10.1021/ic501641q
Ca14Al9.95Zn6O35Mn0.05	https://doi.org/10.1021/ic501641q
Ca14Al9.9Zn6O35Mn0.1	https://doi.org/10.1021/ic501641q
Ca14Al9.8Zn6O35Mn0.2	https://doi.org/10.1021/ic501641q
Ca14Al9.75Zn6O35Mn0.25	https://doi.org/10.1021/ic501641q
Ca14Al9.7Zn6O35Mn0.3	https://doi.org/10.1021/ic501641q
Ca14Al9.85Zn5.5Mg0.5O35Mn0.15	https://doi.org/10.1021/ic501641q
Ca14Al9.85Zn5Mg1O35Mn0.15	https://doi.org/10.1021/ic501641q
Ca14Al9.85Zn4.5Mg1.5O35Mn0.15	https://doi.org/10.1021/ic501641q
Ca13.5Sc0.5Al9.85Zn6O35Mn0.15	https://doi.org/10.1021/ic501641q
Ca13.5Y0.5Al9.85Zn6O35Mn0.15	https://doi.org/10.1021/ic501641q
Ca13.5La0.5Al9.85Zn6O35Mn0.15	https://doi.org/10.1021/ic501641q
Ca13.5Gd0.5Al9.85Zn6O35Mn0.15	https://doi.org/10.1021/ic501641q
Ca13.5Lu0.5Al9.85Zn6O35Mn0.15	https://doi.org/10.1021/ic501641q
LiAl0.9985O2Mn0.001	https://doi.org/10.1016/j.jlumin.2012.12.012
LiAl0.9995O2Mn0.0005	https://doi.org/10.1016/j.jlumin.2012.12.012
LiAl0.999O2Mn0.0015	https://doi.org/10.1016/j.jlumin.2012.12.012
LiAl0.9985O2Mn0.02	https://doi.org/10.1016/j.jlumin.2012.12.012
Gd2ZnTi0.9999O6Mn0.002	https://doi.org/10.1039/C6TC00313C

Gd2ZnTi0.9999O6Mn0.0001	https://doi.org/10.1039/C6TC00313C
Gd2ZnTi0.9999O6Mn0.0005	https://doi.org/10.1039/C6TC00313C
Gd2ZnTi0.9999O6Mn0.001	https://doi.org/10.1039/C6TC00313C
Gd2ZnTi0.9999O6Mn0.004	https://doi.org/10.1039/C6TC00313C
Gd2ZnTi0.9999O6Mn0.008	https://doi.org/10.1039/C6TC00313C
CaMg2Al15.998O27Mn0.08	https://doi.org/10.1021/am507316b
CaMg2Al15.984O27Mn0.016	https://doi.org/10.1021/am507316b
CaMg2Al15.968O27Mn0.032	https://doi.org/10.1021/am507316b
CaMg2Al15.94O27Mn0.16	https://doi.org/10.1021/am507316b
CaMg2Al15.86O27Mn0.24	https://doi.org/10.1021/am507316b
CaMg2Al15.62O27Mn0.48	https://doi.org/10.1021/am507316b
N2H8Ti0.99F4Mn0.01	https://doi.org/10.1039/C6RA16417J
N2H8Ti0.98F6Mn0.02	https://doi.org/10.1039/C6RA16417J
N2H8Ti0.97F6Mn0.03	https://doi.org/10.1039/C6RA16417J
N2H8Ti0.96F6Mn0.04	https://doi.org/10.1039/C6RA16417J
N2H8Ti0.95F6Mn0.05	https://doi.org/10.1039/C6RA16417J
N2H8Ti0.94F6Mn0.06	https://doi.org/10.1039/C6RA16417J
N2H8Ti0.92F6Mn0.08	https://doi.org/10.1039/C6RA16417J
N2H8Ti0.84F6Mn0.16	https://doi.org/10.1039/C6RA16417J
N2H8Si0.99F4Mn0.01	https://doi.org/10.1039/C6RA16417J
N2H8Si0.98F6Mn0.02	https://doi.org/10.1039/C6RA16417J
N2H8Si0.97F6Mn0.03	https://doi.org/10.1039/C6RA16417J
N2H8Si0.96F6Mn0.04	https://doi.org/10.1039/C6RA16417J
N2H8Si0.95F6Mn0.05	https://doi.org/10.1039/C6RA16417J
N2H8Si0.94F6Mn0.06	https://doi.org/10.1039/C6RA16417J
N2H8Si0.92F6Mn0.08	https://doi.org/10.1039/C6RA16417J
N2H8Si0.84F6Mn0.16	https://doi.org/10.1039/C6RA16417J
ZnTi0.98F6(H12O6)Mn0.02	https://doi.org/10.1016/j.jallcom.2015.12.075
ZnTi0.995F6(H12O6)Mn0.005	https://doi.org/10.1016/j.jallcom.2015.12.075
ZnTi0.965F6(H12O6)Mn0.035	https://doi.org/10.1016/j.jallcom.2015.12.075
ZnTi0.95F6(H12O6)Mn0.05	https://doi.org/10.1016/j.jallcom.2015.12.075

ZnTi0.935F6(H12O6)Mn0.065	https://doi.org/10.1016/j.jallcom.2015.12.075
ZnTi0.92F6(H12O6)Mn0.08	https://doi.org/10.1016/j.jallcom.2015.12.075
ZnTi0.905F6(H12O6)Mn0.095	https://doi.org/10.1016/j.jallcom.2015.12.075
ZnTi0.89F6(H12O6)Mn0.11	https://doi.org/10.1016/j.jallcom.2015.12.075
ZnTi0.875F6(H12O6)Mn0.125	https://doi.org/10.1016/j.jallcom.2015.12.075
ZnTi0.785F6(H12O6)Mn0.215	https://doi.org/10.1016/j.jallcom.2015.12.075
BaMgAl9.96O16Mg0.02Mn0.02	https://doi.org/10.1021/acs.chemmater.6b01303
BaMgAl9.99O16Mg0.005Mn0.005	https://doi.org/10.1021/acs.chemmater.6b01303
BaMgAl9.98O16Mg0.01Mn0.01	https://doi.org/10.1021/acs.chemmater.6b01303
BaMgAl9.94O16Mg0.03Mn0.03	https://doi.org/10.1021/acs.chemmater.6b01303
BaMgAl9.92O16Mg0.04Mn0.04	https://doi.org/10.1021/acs.chemmater.6b01303
BaMgAl9.9O16Mg0.05Mn0.05	https://doi.org/10.1021/acs.chemmater.6b01303
Sr4Al14O25Mn0.001	https://doi.org/10.1111/jace.12391
Sr4Al14O25Mn0.0001	https://doi.org/10.1111/jace.12391
Sr4Al14O25Mn0.0005	https://doi.org/10.1111/jace.12391
Sr4Al14O25Mn0.002	https://doi.org/10.1111/jace.12391
Sr4Al14O25Mn0.003	https://doi.org/10.1111/jace.12391
Sr4Al14O25Mn0.005	https://doi.org/10.1111/jace.12391
Sr4Al14O25Mn0.01	https://doi.org/10.1111/jace.12391
Sr4Al14O25Mn0.02	https://doi.org/10.1111/jace.12391
Sr4Al14O25Mn0.025	https://doi.org/10.1111/jace.12391
Y3Al4.919Mg0.08O12Mn0.001	https://doi.org/10.1039/C5TC04133C
Y3Al4.989Mg0.01O12Mn0.001	https://doi.org/10.1039/C5TC04133C
Y3Al4.979Mg0.02O12Mn0.001	https://doi.org/10.1039/C5TC04133C
Y3Al4.959Mg0.04O12Mn0.001	https://doi.org/10.1039/C5TC04133C
Y3Al4.879Mg0.12O12Mn0.001	https://doi.org/10.1039/C5TC04133C
Y3Al4.9195Mg0.08O12Mn0.0005	https://doi.org/10.1039/C5TC04133C
Y3Al4.9175Mg0.08O12Mn0.0025	https://doi.org/10.1039/C5TC04133C
Y3Al4.9145Mg0.08O12Mn0.005	https://doi.org/10.1039/C5TC04133C
Y3Al4.91Mg0.08O12Mn0.01	https://doi.org/10.1039/C5TC04133C
Y3Al4.88Mg0.08O12Mn0.02	https://doi.org/10.1039/C5TC04133C

Y3Al4.919Ge0.08O12Mn0.001	https://doi.org/10.1039/C5TC04133C
Y3Al4.9195Ge0.08O12Mn0.0005	https://doi.org/10.1039/C5TC04133C
Y3Al4.9175Ge0.08O12Mn0.0025	https://doi.org/10.1039/C5TC04133C
Y3Al4.9145Ge0.08O12Mn0.005	https://doi.org/10.1039/C5TC04133C
Y3Al4.91Ge0.08O12Mn0.01	https://doi.org/10.1039/C5TC04133C
Y3Al4.88Ge0.08O12Mn0.02	https://doi.org/10.1039/C5TC04133C
Y3Al4.919Ca0.08O12Mn0.001	https://doi.org/10.1039/C5TC04133C
Y3Al4.9195Ca0.08O12Mn0.0005	https://doi.org/10.1039/C5TC04133C
Y3Al4.9175Ca0.08O12Mn0.0025	https://doi.org/10.1039/C5TC04133C
Y3Al4.9145Ca0.08O12Mn0.005	https://doi.org/10.1039/C5TC04133C
Y3Al4.91Ca0.08O12Mn0.01	https://doi.org/10.1039/C5TC04133C
Y3Al4.88Ca0.08O12Mn0.02	https://doi.org/10.1039/C5TC04133C
Y3Al4.999O12Mn0.0005	https://doi.org/10.1039/C5TC04133C
Y3Al4.9175O12Mn0.0025	https://doi.org/10.1039/C5TC04133C
Y3Al4.9145O12Mn0.005	https://doi.org/10.1039/C5TC04133C
Y3Al4.91O12Mn0.01	https://doi.org/10.1039/C5TC04133C
Y3Al4.88O12Mn0.02	https://doi.org/10.1039/C5TC04133C
Y3Al4.999O12Mn0.001	https://doi.org/10.1039/C5TC04133C
Mg2Ti0.9999O4Mn0.0001	https://doi.org/10.1039/C5TC04133C
Mg2Ti9.9995O4Mn0.0005	https://doi.org/10.1039/C5TC04133C
Mg2Ti9.999O4Mn0.001	https://doi.org/10.1039/C5TC04133C
Mg2Ti9.9975O4Mn0.0025	https://doi.org/10.1039/C5TC04133C
Mg2Ti9.999O2Mn0.001	https://doi.org/10.1039/C5TC04133C
Mg2Ti0.9999O4Mn0.0001	https://doi.org/10.1039/C3TC30553H
Mg2Ti9.9995O4Mn0.0005	https://doi.org/10.1039/C3TC30553H
Mg2Ti9.999O4Mn0.001	https://doi.org/10.1039/C3TC30553H
Mg2Ti9.9975O4Mn0.0025	https://doi.org/10.1039/C3TC30553H
Mg1.998Ti9.999Bi0.001Li0.001O2Mn0.001	https://doi.org/10.1016/j.jlumin.2014.09.032
Mg1.996Ti9.999Bi0.002Li0.002O2Mn0.001	https://doi.org/10.1016/j.jlumin.2014.09.032
Mg1.994Ti9.999Bi0.003Li0.003O2Mn0.001	https://doi.org/10.1016/j.jlumin.2014.09.032
Mg1.992Ti9.999Bi0.004Li0.004O2Mn0.001	https://doi.org/10.1016/j.jlumin.2014.09.032

Mg1.99Ti9.999Bi0.001Li0.005O2Mn0.001	https://doi.org/10.1016/j.jlumin.2014.09.032
CaAl11.99O19Mn0.01	https://doi.org/10.1016/j.mseb.2011.12.016
CaAl11.985Ge0.005O19Mn0.01	https://doi.org/10.1016/j.mseb.2011.12.016
CaAl11.98Ge0.01O19Mn0.01	https://doi.org/10.1016/j.mseb.2011.12.016
CaAl11.975Ge0.015O19Mn0.01	https://doi.org/10.1016/j.mseb.2011.12.016
CaAl11.97Ge0.02O19Mn0.01	https://doi.org/10.1016/j.mseb.2011.12.016
CaAl11.965Ge0.025O19Mn0.01	https://doi.org/10.1016/j.mseb.2011.12.016
CaAl11.96Ge0.03O19Mn0.01	https://doi.org/10.1016/j.mseb.2011.12.016
CaAl3.99O7Mn0.01	https://doi.org/10.1016/j.ceramint.2012.10.149
CaAl3.999O7Mn0.001	https://doi.org/10.1016/j.ceramint.2012.10.149
CaAl3.997O7Mn0.003	https://doi.org/10.1016/j.ceramint.2012.10.149
CaAl3.995O7Mn0.005	https://doi.org/10.1016/j.ceramint.2012.10.149
CaAl3.98O7Mn0.02	https://doi.org/10.1016/j.ceramint.2012.10.149
CaAl3.97O7Mn0.03	https://doi.org/10.1016/j.ceramint.2012.10.149
CaAl3.95O7Mn0.05	https://doi.org/10.1016/j.ceramint.2012.10.149
SrAl11.9997O19Mn0.0003	https://doi.org/10.1002/pssa.201228815
SrAl11.9995O19Mn0.0005	https://doi.org/10.1002/pssa.201228815
SrAl11.999O19Mn0.001	https://doi.org/10.1002/pssa.201228815
SrAl11.997O19Mn0.003	https://doi.org/10.1002/pssa.201228815
SrAl11.995O19Mn0.005	https://doi.org/10.1002/pssa.201228815
SrAl11.995Li0.05O19Mn0.005	https://doi.org/10.1002/pssa.201228815
SrAl11.995Na0.05O19Mn0.005	https://doi.org/10.1002/pssa.201228815
SrAl11.995Mg0.05O19Mn0.005	https://doi.org/10.1002/pssa.201228815
SrAl11.995K0.05O19Mn0.005	https://doi.org/10.1002/pssa.201228815
Sr4Na0Al13.995O25Mn0.01	https://doi.org/10.1016/j.matchemphys.2014.12.041
Sr3.99Na0.02Al13.995O25Mn0.01	https://doi.org/10.1016/j.matchemphys.2014.12.041
Sr3.98Na0.04Al13.99O25Mn0.01	https://doi.org/10.1016/j.matchemphys.2014.12.041
Sr3.975Na0.05Al13.995O25Mn0.01	https://doi.org/10.1016/j.matchemphys.2014.12.041
Sr3.965Na0.07Al13.995O25Mn0.01	https://doi.org/10.1016/j.matchemphys.2014.12.041
Sr3.955Na0.09Al13.995O25Mn0.01	https://doi.org/10.1016/j.matchemphys.2014.12.041
Sr4Al13.9986O25Mn0.0014	https://doi.org/10.1016/j.jallcom.2012.09.139

Sr4Al13.997O25Mn0.003	https://doi.org/10.1016/j.jallcom.2012.09.139
Sr4Al13.993O25Mn0.007	https://doi.org/10.1016/j.jallcom.2012.09.139
Sr4Al13.986O25Mn0.014	https://doi.org/10.1016/j.jallcom.2012.09.139
Sr4Al13.972O25Mn0.028	https://doi.org/10.1016/j.jallcom.2012.09.139
Sr4Al13.958O25Mn0.042	https://doi.org/10.1016/j.jallcom.2012.09.139
Sr4Al13.944O25Mn0.056	https://doi.org/10.1016/j.jallcom.2012.09.139
Sr4Al13.993O25Mn0.007Cl0.044	https://doi.org/10.1016/j.jallcom.2012.09.139
Sr4Al13.993O25Mn0.007Cl0.087	https://doi.org/10.1016/j.jallcom.2012.09.139
Sr4Al13.993O25Mn0.007Cl0.131	https://doi.org/10.1016/j.jallcom.2012.09.139
Sr4Al13.993O25Mn0.007Cl0.175	https://doi.org/10.1016/j.jallcom.2012.09.139
Sr4Al13.993O25Mn0.007Pb0.0033	https://doi.org/10.1016/j.jallcom.2012.09.139
Sr4Al13.993O25Mn0.007Pb0.0066	https://doi.org/10.1016/j.jallcom.2012.09.139
Sr4Al13.993O25Mn0.007Pb0.01	https://doi.org/10.1016/j.jallcom.2012.09.139
Sr4Al13.993O25Mn0.007Pb0.0131	https://doi.org/10.1016/j.jallcom.2012.09.139
Sr4Al13.9997O25Mn0.0003	https://doi.org/10.1016/j.materresbull.2014.08.055
Sr4Al13.9995O25Mn0.0005	https://doi.org/10.1016/j.materresbull.2014.08.055
Sr4Al13.999O25Mn0.001	https://doi.org/10.1016/j.materresbull.2014.08.055
Sr4Al13.998O25Mn0.002	https://doi.org/10.1016/j.materresbull.2014.08.055
Sr4Al13.995O25Mn0.005	https://doi.org/10.1016/j.materresbull.2014.08.055
Sr4Al13.99O25Mn0.01	https://doi.org/10.1016/j.materresbull.2014.08.055
SrMgAl9.99O17Mn0.01	https://doi.org/10.1166/jnn.2016.11793
SrMgAl9.998O17Mn0.002	https://doi.org/10.1166/jnn.2016.11793
SrMgAl9.996O17Mn0.004	https://doi.org/10.1166/jnn.2016.11793
SrMgAl9.994O17Mn0.006	https://doi.org/10.1166/jnn.2016.11793
SrMgAl9.992O17Mn0.008	https://doi.org/10.1166/jnn.2016.11793
SrMgAl9.988O17Mn0.012	https://doi.org/10.1166/jnn.2016.11793
SrMgAl9.986O17Mn0.014	https://doi.org/10.1166/jnn.2016.11793
SrMgAl9.984O17Mn0.016	https://doi.org/10.1166/jnn.2016.11793
LaMg0.4985Ti0.4985O3Mn0.0025	https://doi.org/10.1021/acsami.6b15866
LaMg0.498Ti0.498O3Mn0.003	https://doi.org/10.1021/acsami.6b15866
LaMg0.496Ti0.496O3Mn0.004	https://doi.org/10.1021/acsami.6b15866

LaMg0.4975Ti0.4975O3Mn0.005	https://doi.org/10.1021/acsami.6b15866
LaMg0.495Ti0.495O3Mn0.01	https://doi.org/10.1021/acsami.6b15866
Li3Na3Al1.9975F12Mn0.0025	https://doi.org/10.1039/C7TC03805D
Li3Na3Al1.995F12Mn0.005	https://doi.org/10.1039/C7TC03805D
Li3Na3Al1.99F12Mn0.01	https://doi.org/10.1039/C7TC03805D
Li3Na3Al1.98F12Mn0.02	https://doi.org/10.1039/C7TC03805D
Li3Na3Al1.96F12Mn0.04	https://doi.org/10.1039/C7TC03805D
Li3Na3Al1.92F12Mn0.08	https://doi.org/10.1039/C7TC03805D
Li3Na3Al0.984F12Mn0.016	https://doi.org/10.1039/C7TC03805D
BaMg6Ti5.999O19Mn0.001	https://doi.org/10.4028/www.scientific.net/MSF.868.73
Mg2Ti0.999O4Mn0.001	https://doi.org/10.4028/www.scientific.net/MSF.868.73
Rb2Zr0.9921F6Mn0.0079	https://doi.org/10.1039/C7TC02196H
Rb2Zr0.98F6Mn0.02	https://doi.org/10.1039/C7TC02196H
Rb2Zr0.961F6Mn0.039	https://doi.org/10.1039/C7TC02196H
Rb2Zr0.924F6Mn0.076	https://doi.org/10.1039/C7TC02196H
Rb2Zr0.863F6Mn0.137	https://doi.org/10.1039/C7TC02196H
La1Al0.999O2.9985Mn0.001	https://doi.org/10.1039/C3TC32075H
La1Al0.997O2.9955Mn0.003	https://doi.org/10.1039/C3TC32075H
La1Al0.995O2.9925Mn0.005	https://doi.org/10.1039/C3TC32075H
La1Al0.99O2.985Mn0.01	https://doi.org/10.1039/C3TC32075H
La1Al0.994O2.991Mn0.005Ge0.001	https://doi.org/10.1039/C3TC32075H
La1Al0.99O2.985Mn0.005Ge0.005	https://doi.org/10.1039/C3TC32075H
La1Al0.985O2.9775Mn0.005Ge0.01	https://doi.org/10.1039/C3TC32075H
La1Al0.945O2.9175Mn0.005Ge0.05	https://doi.org/10.1039/C3TC32075H
Ca14Zn6Ga9.97O35Mn0.03	https://doi.org/10.1039/C7RA02112G
Ca14Zn6Ga9.88O35Mn0.12	https://doi.org/10.1039/C7RA02112G
Ca14Zn6Ga9.75O35Mn0.25	https://doi.org/10.1039/C7RA02112G
Ca14Zn6Ga9.91O35Mn0.09	https://doi.org/10.1039/C7RA02112G
Ca14Zn6Ga9.85O35Mn0.15	https://doi.org/10.1039/C7RA02112G
Ca14Zn6Ga9.80O35Mn0.20	https://doi.org/10.1039/C7RA02112G
K3W0.09O2F5Â·2H2OMn0.01	https://doi.org/10.1016/j.jallcom.2022.165522

K3W0.08O2F5Â·2H2OMn0.02	https://doi.org/10.1016/j.jallcom.2022.165522
K3W0.06O2F5Â·2H2OMn0.04	https://doi.org/10.1016/j.jallcom.2022.165522
K3W0.04O2F5Â·2H2OMn0.06	https://doi.org/10.1016/j.jallcom.2022.165522
K3W0.02O2F5Â·2H2OMn0.08	https://doi.org/10.1016/j.jallcom.2022.165522
Sr4Al140.995O25Mn0.005	https://doi.org/10.1016/j.jallcom.2022.165522
Ba2LaNb0.999O6Mn0.001	https://doi.org/10.1016/j.jlumin.2023.119683
Ba2LaNb0.991O6Mn0.009	https://doi.org/10.1016/j.jlumin.2023.119683
(Ca,Sr)2InNb0.999O6Mn0.001	https://doi.org/10.1016/j.jallcom.2022.166498
(Ca,Sr)2InNb0.997O6Mn0.003	https://doi.org/10.1016/j.jallcom.2022.166498
(Ca,Sr)2InNb0.995O6Mn0.005	https://doi.org/10.1016/j.jallcom.2022.166498
(Ca,Sr)2InNb0.993O6Mn0.007	https://doi.org/10.1016/j.jallcom.2022.166498
(Ca,Sr)2InNb0.991O6Mn0.009	https://doi.org/10.1016/j.jallcom.2022.166498
LaSrAL0.996O4Mn0.004	https://doi.org/10.1016/j.molstruc.2022.133484
LaSrAL0.996O4Mn0.001	https://doi.org/10.1016/j.molstruc.2022.133484
LaSrAL0.996O4Mn0.002	https://doi.org/10.1016/j.molstruc.2022.133484
LaSrAL0.996O4Mn0.006	https://doi.org/10.1016/j.molstruc.2022.133484
LaSrAL0.996O4Mn0.008	https://doi.org/10.1016/j.molstruc.2022.133484
LaSrGa0.996O4Mn0.004	https://doi.org/10.1016/j.molstruc.2022.133484
LaSrGa0.996O4Mn0.001	https://doi.org/10.1016/j.molstruc.2022.133484
LaSrGa0.996O4Mn0.002	https://doi.org/10.1016/j.molstruc.2022.133484
LaSrGa0.996O4Mn0.006	https://doi.org/10.1016/j.molstruc.2022.133484
LaSrGa0.996O4Mn0.008	https://doi.org/10.1016/j.molstruc.2022.133484
BaLaCa0.999MgSbO6Mn0.001	https://doi.org/10.1016/j.cej.2021.132924
BaLaCa0.997Mg?SbO6Mn0.003	https://doi.org/10.1016/j.cej.2021.132924
BaLaCa0.995Mg?SbO6Mn0.005	https://doi.org/10.1016/j.cej.2021.132924
BaLaCa0.993Mg?SbO6Mn0.007	https://doi.org/10.1016/j.cej.2021.132924
BaLaCa0.991Mg?SbO6Mn0.009	https://doi.org/10.1016/j.cej.2021.132924
SrLa2Al20.995O7Mn0.005	https://doi.org/10.1016/j.jlumin.2023.120163
SrLa2Al20.999O7Mn0.001	https://doi.org/10.1016/j.jlumin.2023.120163
SrLa2Al20.998O7Mn0.002	https://doi.org/10.1016/j.jlumin.2023.120163
SrLa2Al20.997O7Mn0.003	https://doi.org/10.1016/j.jlumin.2023.120163

SrLa2Al20.996O7Mn0.004	https://doi.org/10.1016/j.jlumin.2023.120163
SrLa2Al20.994O7Mn0.006	https://doi.org/10.1016/j.jlumin.2023.120163
SrLa2Al20.993O7Mn0.007	https://doi.org/10.1016/j.jlumin.2023.120163
Ba2LuNb0.994O6Mn0.006	https://doi.org/10.1016/j.mtcomm.2022.104817
Ba2LuNb1O6Mn0.000	https://doi.org/10.1016/j.mtcomm.2022.104817
Ba2LuNb0.998O6Mn0.002	https://doi.org/10.1016/j.mtcomm.2022.104817
Ba2LuNb0.996O6Mn0.004	https://doi.org/10.1016/j.mtcomm.2022.104817
Ba2LuNb0.992O6Mn0.008	https://doi.org/10.1016/j.mtcomm.2022.104817
Ba2LaTa0.996O6Mn0.004	https://doi.org/10.1016/j.jlumin.2022.118752
Ba2LaTa0.999O6Mn0.001	https://doi.org/10.1016/j.jlumin.2022.118752
Ba2LaTa0.998O6Mn0.002	https://doi.org/10.1016/j.jlumin.2022.118752
Ba2LaTa0.997O6Mn0.003	https://doi.org/10.1016/j.jlumin.2022.118752
Ba2LaTa0.995O6Mn0.005	https://doi.org/10.1016/j.jlumin.2022.118752
Gd2ZnTi0.999O6Mn0.001	https://doi.org/10.1016/j.cej.2022.136839
Gd2ZnTi0.997O6Mn0.003	https://doi.org/10.1016/j.cej.2022.136839
Gd2ZnTi0.995O6Mn0.005	https://doi.org/10.1016/j.cej.2022.136839
Gd2ZnTi0.993O6Mn0.007	https://doi.org/10.1016/j.cej.2022.136839
Gd2ZnTi0.991O6Mn0.009	https://doi.org/10.1016/j.cej.2022.136839
Gd2ZnTi0.99O6Mn0.01	https://doi.org/10.1016/j.cej.2022.136839
Gd2ZnTi0.98O6Mn0.02	https://doi.org/10.1016/j.cej.2022.136839
K1Rb1Si0.982F6Mn0.018	https://doi.org/10.1021/acsami.3c13715
K1Rb1Si0.999F6Mn0.001	https://doi.org/10.1021/acsami.3c13715
K1Rb1Si0.995F6Mn0.005	https://doi.org/10.1021/acsami.3c13715
K1Rb1Si0.99F6Mn0.01	https://doi.org/10.1021/acsami.3c13715
K1Rb1Si0.97F6Mn0.03	https://doi.org/10.1021/acsami.3c13715
K1Rb1Si0.95F6Mn0.05	https://doi.org/10.1021/acsami.3c13715
Li3La3W2O12Mn0.006	https://doi.org/10.1016/j.mtchem.2023.101584
Li3La3W2O12Mn0.01	https://doi.org/10.1016/j.mtchem.2023.101584
Li3La3W2O12Mn0.012	https://doi.org/10.1016/j.mtchem.2023.101584
Li3La3W2O12Mn0.015	https://doi.org/10.1016/j.mtchem.2023.101584
Li3La3W2O12Mn0.02	https://doi.org/10.1016/j.mtchem.2023.101584

K3Zr0.98174F7Mn0.01826	https://doi.org/10.1016/j.jlumin.2023.119884
K3Zr0.9779F7Mn0.01221	https://doi.org/10.1016/j.jlumin.2023.119884
K3Zr0.9825F7Mn0.0175	https://doi.org/10.1016/j.jlumin.2023.119884
K3Zr0.99225F7Mn0.00885	https://doi.org/10.1016/j.jlumin.2023.119884
K3Zr0.99372F7Mn0.00627	https://doi.org/10.1016/j.jlumin.2023.119884
K3Zr0.99618F7Mn0.00382	https://doi.org/10.1016/j.jlumin.2023.119884
K3Zr0.99941F7Mn0.00059	https://doi.org/10.1016/j.jlumin.2023.119884
K3Zr0.99986F7Mn0.00014	https://doi.org/10.1016/j.jlumin.2023.119884
K2Si0.95F6Mn0.05	https://doi.org/10.1016/j.ceramint.2022.08.289
K2Si0.99F6Mn0.01	https://doi.org/10.1016/j.ceramint.2022.08.289
K2Si0.97F6Mn0.03	https://doi.org/10.1016/j.ceramint.2022.08.289
K2Si0.93F6Mn0.07	https://doi.org/10.1016/j.ceramint.2022.08.289
K2Si0.91F6Mn0.09	https://doi.org/10.1016/j.ceramint.2022.08.289
K2Si0.94F6Mn0.06	https://doi.org/10.1016/j.ceramint.2022.08.289
K2Si0.98F6Mn0.02	https://doi.org/10.1016/j.ceramint.2022.08.289
K2Si0.96F6Mn0.04	https://doi.org/10.1016/j.ceramint.2022.08.289
K2Si0.92F6Mn0.08	https://doi.org/10.1016/j.ceramint.2022.08.289
K2Si0.9F6Mn0.10	https://doi.org/10.1016/j.ceramint.2022.08.289
K2Si0.82F6Mn0.12	https://doi.org/10.1016/j.ceramint.2022.08.289
K2Si0.86F6Mn0.14	https://doi.org/10.1016/j.ceramint.2022.08.289
K3Al0.94F6Mn0.06	https://doi.org/10.1016/j.optmat.2022.112552
K3Al0.99F6Mn0.01	https://doi.org/10.1016/j.optmat.2022.112552
K3Al0.98F6Mn0.02	https://doi.org/10.1016/j.optmat.2022.112552
K3Al0.97F6Mn0.03	https://doi.org/10.1016/j.optmat.2022.112552
K3Al0.96F6Mn0.04	https://doi.org/10.1016/j.optmat.2022.112552
K3Al0.95F6Mn0.05	https://doi.org/10.1016/j.optmat.2022.112552
K3Al0.93F6Mn0.07	https://doi.org/10.1016/j.optmat.2022.112552
K3Al0.92F6Mn0.08	https://doi.org/10.1016/j.optmat.2022.112552
K3Al0.91F6Mn0.09	https://doi.org/10.1016/j.optmat.2022.112552
Sr2InSb0.999O6Mn0.001	https://doi.org/10.1016/j.jallcom.2021.162042
Sr2InSb0.9995O6Mn0.0005	https://doi.org/10.1016/j.jallcom.2021.162042

Sr2InSb0.995O6Mn0.005	https://doi.org/10.1016/j.jallcom.2021.162042
Sr2InSb0.97O6Mn0.03	https://doi.org/10.1016/j.jallcom.2021.162042
Sr2InSb0.98O6Mn0.02	https://doi.org/10.1016/j.jallcom.2021.162042
Sr2InSb0.99O6Mn0.01	https://doi.org/10.1016/j.jallcom.2021.162042
Ca2YInSb0.994Ta0.4O6Mn0.006	https://doi.org/10.1016/j.mtchem.2022.101006
Ca2YInSb0.998Ta0.4O6Mn0.002	https://doi.org/10.1016/j.mtchem.2022.101006
Ca2YInSb0.996Ta0.4O6Mn0.004	https://doi.org/10.1016/j.mtchem.2022.101006
Ca2YInSb0.992Ta0.4O6Mn0.008	https://doi.org/10.1016/j.mtchem.2022.101006
Ca2YInSb0.99Ta0.4O6Mn0.010	https://doi.org/10.1016/j.mtchem.2022.101006
Ca2YInSb0.985Ta0.4O6Mn0.015	https://doi.org/10.1016/j.mtchem.2022.101006
Ba3In20.98F12Mn0.02	https://doi.org/10.1016/j.jlumin.2021.118564
Ba3In20.99F12Mn0.01	https://doi.org/10.1016/j.jlumin.2021.118564
Ba3In20.96F12Mn0.04	https://doi.org/10.1016/j.jlumin.2021.118564
Ba3In20.975F12Mn0.025	https://doi.org/10.1016/j.jlumin.2021.118564
Ba3In20.995F12Mn0.005	https://doi.org/10.1016/j.jlumin.2021.118564
Ba3In20.92F12Mn0.08	https://doi.org/10.1016/j.jlumin.2021.118564
Ca2YNb0.996O6Mn0.004	https://doi.org/10.1016/j.jssc.2021.122840
Ca2YNb0.998O6Mn0.002	https://doi.org/10.1016/j.jssc.2021.122840
Ca2YNb0.997O6Mn0.003	https://doi.org/10.1016/j.jssc.2021.122840
Ca2YNb0.995O6Mn0.005	https://doi.org/10.1016/j.jssc.2021.122840
Ca2YNb0.993O6Mn0.007	https://doi.org/10.1016/j.jssc.2021.122840
Ca2YNb0.991O6Mn0.009	https://doi.org/10.1016/j.jssc.2021.122840
LaGe0.996SbO6Mn0.004	https://doi.org/10.1016/j.jlumin.2023.119767
LaGe0.998SbO6Mn0.002	https://doi.org/10.1016/j.jlumin.2023.119767
LaGe0.994SbO6Mn0.006	https://doi.org/10.1016/j.jlumin.2023.119767
LaGe0.992SbO6Mn0.008	https://doi.org/10.1016/j.jlumin.2023.119767
LaGe0.99SbO6Mn0.010	https://doi.org/10.1016/j.jlumin.2023.119767
LaGe0.98SbO6Mn0.012	https://doi.org/10.1016/j.jlumin.2023.119767
LaGe0.96SbO6Mn0.014	https://doi.org/10.1016/j.jlumin.2023.119767
Sr2ScSb0.998O6Mn0.002	https://doi.org/10.1016/j.ceramint.2022.12.249
Sr2ScSb0.999O6Mn0.001	https://doi.org/10.1016/j.ceramint.2022.12.249

Sr2ScSb0.996O6Mn0.004	https://doi.org/10.1016/j.ceramint.2022.12.249
Sr2ScSb0.994O6Mn0.006	https://doi.org/10.1016/j.ceramint.2022.12.249
Sr2ScSb0.992O6Mn0.008	https://doi.org/10.1016/j.ceramint.2022.12.249
Sr2ScSb0.99O6Mn0.010	https://doi.org/10.1016/j.ceramint.2022.12.249
BaNbF0.97O1.5H1.5Mn0.03	https://doi.org/10.1016/j.jallcom.2023.168806
BaNbF0.95O1.5H1.5Mn0.05	https://doi.org/10.1016/j.jallcom.2023.168806
BaNbF0.93O1.5H1.5Mn0.07	https://doi.org/10.1016/j.jallcom.2023.168806
BaNbF0.91O1.5H1.5Mn0.09	https://doi.org/10.1016/j.jallcom.2023.168806
BaNbF0.99O1.5H1.5Mn0.01	https://doi.org/10.1016/j.jallcom.2023.168806
BaGa0.972F5Mn0.028	https://doi.org/10.1016/j.jallcom.2023.168806
BaGa0.986F5Mn0.014	https://doi.org/10.1016/j.jallcom.2023.168806
BaGa0.956F5Mn0.044	https://doi.org/10.1016/j.jallcom.2023.168806
BaGa0.94F5Mn0.060	https://doi.org/10.1016/j.jallcom.2023.168806
LaTi0.997SbO6Mn0.003	https://doi.org/10.1016/j.ceramint.2022.06.206
LaTi0.995SbO6Mn0.005	https://doi.org/10.1016/j.ceramint.2022.06.206
LaTi0.993SbO6Mn0.007	https://doi.org/10.1016/j.ceramint.2022.06.206
LaTi0.991SbO6Mn0.009	https://doi.org/10.1016/j.ceramint.2022.06.206
LaTi0.988SbO6Mn0.011	https://doi.org/10.1016/j.ceramint.2022.06.206
LaTi0.999SbO6Mn0.001	https://doi.org/10.1016/j.ceramint.2022.06.206
K2Ge0.2Ti0.8F6Mn0.01	https://doi.org/10.1016/j.ceramint.2023.06.022
K2Ge0.4Ti0.6F6Mn0.01	https://doi.org/10.1016/j.ceramint.2023.06.022
K2Ge0.5Ti0.5F6Mn0.01	https://doi.org/10.1016/j.ceramint.2023.06.022
K2Ge0.6Ti0.4F6Mn0.01	https://doi.org/10.1016/j.ceramint.2023.06.022
K2Ge0.8Ti0.8F6Mn0.01	https://doi.org/10.1016/j.ceramint.2023.06.022
K2GeF6Mn0.01	https://doi.org/10.1016/j.ceramint.2023.06.022
K2Ti0.985F6Mn0.015	https://doi.org/10.1016/j.jlumin.2022.118728
K2Ti0.9875F6Mn0.0125	https://doi.org/10.1016/j.jlumin.2022.118728
K2Ti0.99F6Mn0.01	https://doi.org/10.1016/j.jlumin.2022.118728
K2Ti0.9925F6Mn0.0075	https://doi.org/10.1016/j.jlumin.2022.118728
K2Ti0.995F6Mn0.005	https://doi.org/10.1016/j.jlumin.2022.118728
K2Ti0.9985F6Mn0.0025	https://doi.org/10.1016/j.jlumin.2022.118728

Ca2InNb0.997O6Mn0.003	https://doi.org/10.1016/j.jlumin.2023.120165
Ca2InNb0.996O6Mn0.004	https://doi.org/10.1016/j.jlumin.2023.120165
Ca2InNb0.995O6Mn0.005	https://doi.org/10.1016/j.jlumin.2023.120165
Ca2InNb0.994O6Mn0.006	https://doi.org/10.1016/j.jlumin.2023.120165
Ca2InNb0.999O6Mn0.001	https://doi.org/10.1016/j.jlumin.2023.120165
Ca2InNb0.998O6Mn0.002	https://doi.org/10.1016/j.jlumin.2023.120165
Ba2LaTa0.997O6Mn0.003	https://doi.org/10.1016/j.mtchem.2022.101109
Ba2LaTa0.995O6Mn0.005	https://doi.org/10.1016/j.mtchem.2022.101109
Ba2LaTa0.993O6Mn0.007	https://doi.org/10.1016/j.mtchem.2022.101109
Ba2LaTa0.991O6Mn0.009	https://doi.org/10.1016/j.mtchem.2022.101109
Ba2LaTa0.999O6Mn0.001	https://doi.org/10.1016/j.mtchem.2022.101109
CaSrMgW0.99O6Mn0.01	https://doi.org/10.1016/j.mtchem.2022.101010
CaSrMgW0.995O6Mn0.005	https://doi.org/10.1016/j.mtchem.2022.101010
CaSrMgW0.985O6Mn0.015	https://doi.org/10.1016/j.mtchem.2022.101010
CaSrMgW0.98O6Mn0.02	https://doi.org/10.1016/j.mtchem.2022.101010
CaLaLiTe0.994O6Mn0.006	https://doi.org/10.1016/j.jallcom.2023.169436
CaLaLiTe0.998O6Mn0.002	https://doi.org/10.1016/j.jallcom.2023.169436
CaLaLiTe0.996O6Mn0.004	https://doi.org/10.1016/j.jallcom.2023.169436
CaLaLiTe0.992O6Mn0.008	https://doi.org/10.1016/j.jallcom.2023.169436
CaLaLiTe0.99O6Mn0.010	https://doi.org/10.1016/j.jallcom.2023.169436
CaLaLiTe0.988O6Mn0.012	https://doi.org/10.1016/j.jallcom.2023.169436
CaLaLiTe0.986O6Mn0.014	https://doi.org/10.1016/j.jallcom.2023.169436
Ba2SrW0.995O6Mn0.005	https://doi.org/10.1016/j.jallcom.2022.168418
Ba2SrW0.994O6Mn0.006	https://doi.org/10.1016/j.jallcom.2022.168418
Ba2SrW0.993O6Mn0.007	https://doi.org/10.1016/j.jallcom.2022.168418
Ba2SrW0.999O6Mn0.001	https://doi.org/10.1016/j.jallcom.2022.168418
Ba2SrW0.998O6Mn0.002	https://doi.org/10.1016/j.jallcom.2022.168418
Ba2SrW0.997O6Mn0.003	https://doi.org/10.1016/j.jallcom.2022.168418
Ba2SrW0.996O6Mn0.004	https://doi.org/10.1016/j.jallcom.2022.168418
BaLaZnSb0.994O6Mn0.006	https://doi.org/10.1016/j.jlumin.2023.120019
BaLaZnSb0.999O6Mn0.001	https://doi.org/10.1016/j.jlumin.2023.120019

BaLaZnSb0.998O6Mn0.002	https://doi.org/10.1016/j.jlumin.2023.120019
BaLaZnSb0.996O6Mn0.004	https://doi.org/10.1016/j.jlumin.2023.120019
BaLaZnSb0.992O6Mn0.008	https://doi.org/10.1016/j.jlumin.2023.120019
BaLaZnSb0.99O6Mn0.010	https://doi.org/10.1016/j.jlumin.2023.120019
BaLaZnSb0.985O6Mn0.015	https://doi.org/10.1016/j.jlumin.2023.120019
Sr2ScTa0.999O6Mn0.001	https://doi.org/10.1016/j.jlumin.2021.118724
Sr2ScTa0.997O6Mn0.003	https://doi.org/10.1016/j.jlumin.2021.118724
Sr2ScTa0.995O6Mn0.005	https://doi.org/10.1016/j.jlumin.2021.118724
Sr2ScTa0.991O6Mn0.009	https://doi.org/10.1016/j.jlumin.2021.118724
Sr2ScTa0.989O6Mn0.011	https://doi.org/10.1016/j.jlumin.2021.118724
Sr2ScTa0.993O6Mn0.007	https://doi.org/10.1016/j.jlumin.2021.118724
Sr2ScNbO6Mn0.004	https://doi.org/10.1016/j.jallcom.2023.171333
Sr2ScNb0.995O6Mn0.005	https://doi.org/10.1016/j.jallcom.2023.171333
Sr2ScNb0.994O6Mn0.006	https://doi.org/10.1016/j.jallcom.2023.171333
Sr2ScNb0.993O6Mn0.007	https://doi.org/10.1016/j.jallcom.2023.171333
Sr2ScNb0.992O6Mn0.008	https://doi.org/10.1016/j.jallcom.2023.171333
Sr2ScNb0.999O6Mn0.001	https://doi.org/10.1016/j.jallcom.2023.171333
Sr2ScNb0.998O6Mn0.002	https://doi.org/10.1016/j.jallcom.2023.171333
Sr2ScNb0.997O6Mn0.003	https://doi.org/10.1016/j.jallcom.2023.171333
SrLa2Al20.994O7Mn0.006	https://doi.org/10.1016/j.jlumin.2023.119759
SrLa2Al20.993O7Mn0.007	https://doi.org/10.1016/j.jlumin.2023.119759
SrLa2Al20.992O7Mn0.008	https://doi.org/10.1016/j.jlumin.2023.119759
SrLa2Al20.999O7Mn0.001	https://doi.org/10.1016/j.jlumin.2023.119759
SrLa2Al20.998O7Mn0.002	https://doi.org/10.1016/j.jlumin.2023.119759
SrLa2Al20.997O7Mn0.003	https://doi.org/10.1016/j.jlumin.2023.119759
SrLa2Al20.996O7Mn0.004	https://doi.org/10.1016/j.jlumin.2023.119759
SrLa2Al20.995O7Mn0.005	https://doi.org/10.1016/j.jlumin.2023.119759
Ba2LaTa0.99O6Mn0.01	https://doi.org/10.1016/j.ceramint.2022.08.170
Ba2LaTa0.988O6Mn0.012	https://doi.org/10.1016/j.ceramint.2022.08.170
Ba2LaTa0.984O6Mn0.016	https://doi.org/10.1016/j.ceramint.2022.08.170
Ba2LaTa0.986O6Mn0.024	https://doi.org/10.1016/j.ceramint.2022.08.170

Ba2LaTa0.998O6Mn0.002	https://doi.org/10.1016/j.ceramint.2022.08.170
Ba2LaTa0.996O6Mn0.004	https://doi.org/10.1016/j.ceramint.2022.08.170
Ba2LaTa0.992O6Mn0.008	https://doi.org/10.1016/j.ceramint.2022.08.170
Na2Nb0.9953OF5Mn0.0047	https://doi.org/10.1016/j.cej.2023.147165
Na2Nb0.9992OF5Mn0.0008	https://doi.org/10.1016/j.cej.2023.147165
Na2Nb0.9872OF5Mn0.0128	https://doi.org/10.1016/j.cej.2023.147165
Na2W0.9953O2F4Mn0.0047	https://doi.org/10.1016/j.ceramint.2023.01.022
Sr2ScNb0.994O6Mn0.006	https://doi.org/10.1016/j.optmat.2023.114212
Sr2ScNb0.992O6Mn0.008	https://doi.org/10.1016/j.optmat.2023.114212
Sr2ScNb0.99O6Mn0.010	https://doi.org/10.1016/j.optmat.2023.114212
Sr2ScNb0.998O6Mn0.002	https://doi.org/10.1016/j.optmat.2023.114212
Sr2ScNb0.996O6Mn0.004	https://doi.org/10.1016/j.optmat.2023.114212
Sr2ScNb0.97O6Mn0.03	https://doi.org/10.1016/j.optmat.2023.114212
K2Nb0.97Mo0.03F7Mn0.005	https://doi.org/10.3390/molecules28114566
K2Nb0.95Mo0.05F7Mn0.005	https://doi.org/10.3390/molecules28114566
K2Nb0.93Mo0.07F7Mn0.005	https://doi.org/10.3390/molecules28114566
K2Nb0.9Mo0.1F7Mn0.005	https://doi.org/10.3390/molecules28114566
K2Nb0.85Mo0.15F7Mn0.007	https://doi.org/10.3390/molecules28114566
K2Nb5MoF7Mn0.007	https://doi.org/10.3390/molecules28114566
Sr2LuNb0.97O6Mn0.03	https://doi.org/10.1016/j.jlumin.2022.119267
Sr2LuNb0.96O6Mn0.04	https://doi.org/10.1016/j.jlumin.2022.119267
Sr2LuNb0.95O6Mn0.05	https://doi.org/10.1016/j.jlumin.2022.119267
Sr2LuNb0.99O6Mn0.01	https://doi.org/10.1016/j.jlumin.2022.119267
Sr2LuNb0.98O6Mn0.02	https://doi.org/10.1016/j.jlumin.2022.119267
Sr2InTa0.998O6Mn0.002	https://doi.org/10.1016/j.jlumin.2022.119351
Sr2InTa0.996O6Mn0.004	https://doi.org/10.1016/j.jlumin.2022.119351
Sr2InTa0.994O6Mn0.006	https://doi.org/10.1016/j.jlumin.2022.119351
Sr2InTa0.992O6Mn0.008	https://doi.org/10.1016/j.jlumin.2022.119351
Sr2InTa0.999O6Mn0.001	https://doi.org/10.1016/j.jlumin.2022.119351
Ba2YSb0.92O6Mn0.08	https://doi.org/10.1016/j.ceramint.2022.09.281
Ba2YSb1O6Mn0.00	https://doi.org/10.1016/j.ceramint.2022.09.281

Ba2YSb0.98O6Mn0.02	https://doi.org/10.1016/j.ceramint.2022.09.281
Ba2YSb0.96O6Mn0.04	https://doi.org/10.1016/j.ceramint.2022.09.281
Ba2YSb0.94O6Mn0.06	https://doi.org/10.1016/j.ceramint.2022.09.281
Ba2YSb0.9O6Mn0.10	https://doi.org/10.1016/j.ceramint.2022.09.281
Ba2YSb0.88O6Mn0.12	https://doi.org/10.1016/j.ceramint.2022.09.281
NaLiTi30.997O7Mn0.003	https://doi.org/10.1016/j.jlumin.2022.119591
NaLiTi30.996O7Mn0.004	https://doi.org/10.1016/j.jlumin.2022.119591
NaLiTi30.995O7Mn0.005	https://doi.org/10.1016/j.jlumin.2022.119591
NaLiTi30.994O7Mn0.006	https://doi.org/10.1016/j.jlumin.2022.119591
NaLiTi30.993O7Mn0.007	https://doi.org/10.1016/j.jlumin.2022.119591
NaLiTi30.992O7Mn0.008	https://doi.org/10.1016/j.jlumin.2022.119591
NaLiTi30.991O7Mn0.009	https://doi.org/10.1016/j.jlumin.2022.119591
NaLiTi30.99O7Mn0.010	https://doi.org/10.1016/j.jlumin.2022.119591
NaLiTi30.999O7Mn0.001	https://doi.org/10.1016/j.jlumin.2022.119591
NaLiTi30.998O7Mn0.002	https://doi.org/10.1016/j.jlumin.2022.119591
Mg2Si0.1Ge0.9O4Mn0.001	https://doi.org/10.1016/j.optmat.2023.113500
Mg2Si0.2Ge0.8O4Mn0.001	https://doi.org/10.1016/j.optmat.2023.113500
Mg2Si0.3Ge0.7O4Mn0.001	https://doi.org/10.1016/j.optmat.2023.113500
Mg2Si0.4Ge0.6O4Mn0.001	https://doi.org/10.1016/j.optmat.2023.113500
Mg2Si0.5Ge0.5O4Mn0.001	https://doi.org/10.1016/j.optmat.2023.113500
Mg2Si0.6Ge0.4O4Mn0.001	https://doi.org/10.1016/j.optmat.2023.113500
Mg2Si0.7Ge0.3O4Mn0.001	https://doi.org/10.1016/j.optmat.2023.113500
Mg2Si0.8Ge0.2O4Mn0.001	https://doi.org/10.1016/j.optmat.2023.113500
Mg2Si0.9Ge0.1O4Mn0.001	https://doi.org/10.1016/j.optmat.2023.113500
La2Mg1.33Ta0.996O6Mn0.004	https://doi.org/10.1016/j.jlumin.2022.119347
La2Mg1.33Ta0.994O6Mn0.006	https://doi.org/10.1016/j.jlumin.2022.119347
La2Mg1.33Ta0.992O6Mn0.008	https://doi.org/10.1016/j.jlumin.2022.119347
La2Mg1.33Ta0.99O6Mn0.010	https://doi.org/10.1016/j.jlumin.2022.119347
La2Mg1.33Ta0.988O6Mn0.012	https://doi.org/10.1016/j.jlumin.2022.119347
La2Mg1.33Ta0.998O6Mn0.002	https://doi.org/10.1016/j.jlumin.2022.119347
La2Mg1.33Ta0.999O6Mn0.001	https://doi.org/10.1016/j.jlumin.2022.119347

La2Mg0.998TiO6Mn0.002	https://doi.org/10.1016/j.jlumin.2023.119778
La2Mg0.995TiO6Mn0.005	https://doi.org/10.1016/j.jlumin.2023.119778
La2Mg0.993TiO6Mn0.007	https://doi.org/10.1016/j.jlumin.2023.119778
La2Mg0.992TiO6Mn0.008	https://doi.org/10.1016/j.jlumin.2023.119778
La2Mg0.991TiO6Mn0.01	https://doi.org/10.1016/j.jlumin.2023.119778
La2Mg0.98TiO6Mn0.02	https://doi.org/10.1016/j.jlumin.2023.119778
La2Mg0.97TiO6Mn0.3	https://doi.org/10.1016/j.jlumin.2023.119778
La2Mg0.96TiO6Mn0.4	https://doi.org/10.1016/j.jlumin.2023.119778
Ba2GdTa0.992O6Mn0.008	https://doi.org/10.1016/j.optlastec.2022.108144
Ba2GdTa0.996O6Mn0.004	https://doi.org/10.1016/j.optlastec.2022.108144
Ba2GdTa0.994O6Mn0.006	https://doi.org/10.1016/j.optlastec.2022.108144
Ba2GdTa0.99O6Mn0.010	https://doi.org/10.1016/j.optlastec.2022.108144
Ba2GdTa0.988O6Mn0.012	https://doi.org/10.1016/j.optlastec.2022.108144
BaLaLiW0.999O6Mn0.001	https://doi.org/10.1016/j.jallcom.2022.167927
BaLaLiW0.995O6Mn0.005	https://doi.org/10.1016/j.jallcom.2022.167927
BaLaLiW0.97O6Mn0.03	https://doi.org/10.1016/j.jallcom.2022.167927
BaLaLiW0.99O6Mn0.010	https://doi.org/10.1016/j.jallcom.2022.167927
BaLaLiW0.98O6Mn0.020	https://doi.org/10.1016/j.jallcom.2022.167927
Mg2ScSb0.999O6Mn0.001	https://doi.org/10.1016/j.jlumin.2022.119624
Mg2ScSb0.995O6Mn0.005	https://doi.org/10.1016/j.jlumin.2022.119624
Mg2ScSb0.99O6Mn0.010	https://doi.org/10.1016/j.jlumin.2022.119624
Mg2ScSb0.98O6Mn0.020	https://doi.org/10.1016/j.jlumin.2022.119624
Mg2ScSb0.97O6Mn0.030	https://doi.org/10.1016/j.jlumin.2022.119624
Sr2GdTa0.994O6Mn0.006	https://doi.org/10.1016/j.mtchem.2021.100737
Sr2GdTa0.999O6Mn0.001	https://doi.org/10.1016/j.mtchem.2021.100737
Sr2GdTa0.997O6Mn0.003	https://doi.org/10.1016/j.mtchem.2021.100737
Sr2GdTa0.995O6Mn0.005	https://doi.org/10.1016/j.mtchem.2021.100737
Sr2GdTa0.993O6Mn0.007	https://doi.org/10.1016/j.mtchem.2021.100737
Sr2GdTa0.991O6Mn0.009	https://doi.org/10.1016/j.mtchem.2021.100737
Sr2GdTa0.99O6Mn0.010	https://doi.org/10.1016/j.mtchem.2021.100737
CaGdMgNb0.995O6Mn0.005	https://doi.org/10.1016/j.mtcomm.2022.103809

CaGdMgNb0.999O6Mn0.001	https://doi.org/10.1016/j.mtcomm.2022.103809
CaGdMgNb0.997O6Mn0.003	https://doi.org/10.1016/j.mtcomm.2022.103809
CaGdMgNb0.993O6Mn0.007	https://doi.org/10.1016/j.mtcomm.2022.103809
CaGdMgNb0.991O6Mn0.009	https://doi.org/10.1016/j.mtcomm.2022.103809
SrKYTe0.996O6Mn0.004	https://doi.org/10.1016/j.jallcom.2022.164243
SrKYTe0.998O6Mn0.002	https://doi.org/10.1016/j.jallcom.2022.164243
SrKYTe0.994O6Mn0.006	https://doi.org/10.1016/j.jallcom.2022.164243
SrKYTe0.992O6Mn0.008	https://doi.org/10.1016/j.jallcom.2022.164243
SrKYTe0.99O6Mn0.010	https://doi.org/10.1016/j.jallcom.2022.164243
SrKYTe0.98O6Mn0.012	https://doi.org/10.1016/j.jallcom.2022.164243
Ca2GdZr20.997Al3O12Mn0.003	https://doi.org/10.1016/j.jlumin.2022.119132
Ca2GdZr20.996Al3O12Mn0.004	https://doi.org/10.1016/j.jlumin.2022.119132
Ca2GdZr20.995Al3O12Mn0.005	https://doi.org/10.1016/j.jlumin.2022.119132
Ca2GdZr20.994Al3O12Mn0.006	https://doi.org/10.1016/j.jlumin.2022.119132
Ca2GdZr20.999Al3O12Mn0.001	https://doi.org/10.1016/j.jlumin.2022.119132
Ca2GdZr20.998Al3O12Mn0.002	https://doi.org/10.1016/j.jlumin.2022.119132
Ca2InNb0.999O6Mn0.001	https://doi.org/10.1016/j.jallcom.2022.166498
Ca2InNb0.997O6Mn0.003	https://doi.org/10.1016/j.jallcom.2022.166498
Ca2InNb0.995O6Mn0.005	https://doi.org/10.1016/j.jallcom.2022.166498
Ca2InNb0.993O6Mn0.007	https://doi.org/10.1016/j.jallcom.2022.166498
Ca2InNb0.991O6Mn0.009	https://doi.org/10.1016/j.jallcom.2022.166498
K3W0.98O2F5Â·2H2OMn0.02	https://doi.org/10.1016/j.jallcom.2022.166498
K3W0.96O2F5Â·2H2OMn0.04	https://doi.org/10.1016/j.jallcom.2022.166498
K3W0.94O2F5Â·2H2OMn0.06	https://doi.org/10.1016/j.jallcom.2022.166498
K3W0.92O2F5Â·2H2OMn0.08	https://doi.org/10.1016/j.jallcom.2022.166498
K3W0.99O2F5Â·2H2OMn0.01	https://doi.org/10.1016/j.jallcom.2022.166498
Na3Li3In20.97F12Mn0.03	https://doi.org/10.1016/j.ceramint.2022.12.059
Na3Li3In20.95F12Mn0.05	https://doi.org/10.1016/j.ceramint.2022.12.059
Na3Li3In20.93F12Mn0.07	https://doi.org/10.1016/j.ceramint.2022.12.059
Na3Li3In20.91F12Mn0.09	https://doi.org/10.1016/j.ceramint.2022.12.059
Na3Li3In2F0.9912Mn0.01	https://doi.org/10.1016/j.ceramint.2022.12.059

KLiSn0.92F6Mn0.08	https://doi.org/10.1016/j.ceramint.2023.09.298
KLiSn0.9F6Mn0.10	https://doi.org/10.1016/j.ceramint.2023.09.298
KLiSn0.88F6Mn0.12	https://doi.org/10.1016/j.ceramint.2023.09.298
KLiSn0.96F6Mn0.04	https://doi.org/10.1016/j.ceramint.2023.09.298
KLiSn0.94F6Mn0.06	https://doi.org/10.1016/j.ceramint.2023.09.298
K2Ti0.95F6Mn0.05	https://doi.org/10.1016/j.apsusc.2022.154461
K2Ti0.94F6Mn0.06	https://doi.org/10.1016/j.apsusc.2022.154461
K2Ti0.93F6Mn0.07	https://doi.org/10.1016/j.apsusc.2022.154461
K2Ti0.92F6Mn0.08	https://doi.org/10.1016/j.apsusc.2022.154461
K2Ti0.99F6Mn0.01	https://doi.org/10.1016/j.apsusc.2022.154461
K2Ti0.98F6Mn0.02	https://doi.org/10.1016/j.apsusc.2022.154461
K2Ti0.97F6Mn0.03	https://doi.org/10.1016/j.apsusc.2022.154461
K2Ti0.96F6Mn0.04	https://doi.org/10.1016/j.apsusc.2022.154461
Ba2CaW0.9925O6Mn0.0075	https://doi.org/10.1016/j.optmat.2022.112052
Ba2CaW0.9985O6Mn0.0015	https://doi.org/10.1016/j.optmat.2022.112052
Ba2CaW0.997O6Mn0.003	https://doi.org/10.1016/j.optmat.2022.112052
Ba2CaW0.9955O6Mn0.0045	https://doi.org/10.1016/j.optmat.2022.112052
Ba2CaW0.994O6Mn0.006	https://doi.org/10.1016/j.optmat.2022.112052
Ba2CaW0.991O6Mn0.009	https://doi.org/10.1016/j.optmat.2022.112052
Ba2CaW0.9895O6Mn0.0105	https://doi.org/10.1016/j.optmat.2022.112052
Ba2CaW0.988O6Mn0.012	https://doi.org/10.1016/j.optmat.2022.112052
Na2W0.99O2F4Mn0.01	https://doi.org/10.1016/j.ceramint.2022.11.186
Na2W0.995O2F4Mn0.005	https://doi.org/10.1016/j.ceramint.2022.11.186
Na2W0.985O2F4Mn0.015	https://doi.org/10.1016/j.ceramint.2022.11.186
Na2W0.98O2F4Mn0.02	https://doi.org/10.1016/j.ceramint.2022.11.186
LaCa0.5Zr0.494O3Mn0.006	https://doi.org/10.1016/j.materresbull.2021.111610
La0.5Ca0.5Zr0.498O3Mn0.002	https://doi.org/10.1016/j.materresbull.2021.111610
La0.5Ca0.5Zr0.496O3Mn0.004	https://doi.org/10.1016/j.materresbull.2021.111610
La0.5Ca0.5Zr0.492O3Mn0.008	https://doi.org/10.1016/j.materresbull.2021.111610
La0.5Ca0.5Zr0.490O3Mn0.010	https://doi.org/10.1016/j.materresbull.2021.111610
La0.5Ca0.5Zr0.488O3Mn0.012	https://doi.org/10.1016/j.materresbull.2021.111610

La0.5Ca0.5Zr0.486O3Mn0.014	https://doi.org/10.1016/j.materresbull.2021.111610
Ba2Y0.8Eu0.2Nb0.993O6Mn0.007	https://doi.org/10.1016/j.mtchem.2021.100710
Ba2Y0.8Eu0.2Nb0.999O6Mn0.001	https://doi.org/10.1016/j.mtchem.2021.100710
Ba2Y0.8Eu0.2Nb0.997O6Mn0.003	https://doi.org/10.1016/j.mtchem.2021.100710
Ba2Y0.8Eu0.2Nb0.995O6Mn0.005	https://doi.org/10.1016/j.mtchem.2021.100710
Ba2Y0.8Eu0.2Nb0.991O6Mn0.009	https://doi.org/10.1016/j.mtchem.2021.100710
Mg2Ti0.994O4Mn0.006	https://doi.org/10.1016/j.jlumin.2022.119417
Mg2Ti0.993O4Mn0.007	https://doi.org/10.1016/j.jlumin.2022.119417
Mg2Ti0.992O4Mn0.008	https://doi.org/10.1016/j.jlumin.2022.119417
Mg2Ti0.991O4Mn0.009	https://doi.org/10.1016/j.jlumin.2022.119417
Mg2Ti0.99O4Mn0.010	https://doi.org/10.1016/j.jlumin.2022.119417
Mg2Ti0.999O4Mn0.001	https://doi.org/10.1016/j.jlumin.2022.119417
Mg2Ti0.997O4Mn0.003	https://doi.org/10.1016/j.jlumin.2022.119417
Mg2Ti0.995O4Mn0.005	https://doi.org/10.1016/j.jlumin.2022.119417
K3Mo0.98O2F5Â·2H2OMn0.02	https://doi.org/10.1016/j.jallcom.2023.171314
K3Mo0.99O2F5Â·2H2OMn0.01	https://doi.org/10.1016/j.jallcom.2023.171314
K3Mo0.96O2F5Â·2H2OMn0.04	https://doi.org/10.1016/j.jallcom.2023.171314
K3Mo0.94O2F5Â·2H2OMn0.06	https://doi.org/10.1016/j.jallcom.2023.171314
K3Mo0.92O2F5Â·2H2OMn0.08	https://doi.org/10.1016/j.jallcom.2023.171314
N2H8NaAl0.98F6Mn0.02	https://doi.org/10.1016/j.jlumin.2022.119242
N2H8NaAl0.97F6Mn0.03	https://doi.org/10.1016/j.jlumin.2022.119242
N2H8NaAl0.96F6Mn0.04	https://doi.org/10.1016/j.jlumin.2022.119242
N2H8NaAl0.95F6Mn0.05	https://doi.org/10.1016/j.jlumin.2022.119242
N2H8NaGa0.95F6Mn0.05	https://doi.org/10.1016/j.jlumin.2022.119242
N2H8NaIn0.95F6Mn0.05	https://doi.org/10.1016/j.jlumin.2022.119242
K0.5La0.5SrMgW0.985O6Mn0.015	https://doi.org/10.1016/j.mtcomm.2022.103214
K0.5La0.5SrMgW0.999O6Mn0.001	https://doi.org/10.1016/j.mtcomm.2022.103214
K0.5La0.5SrMgW0.994O6Mn0.006	https://doi.org/10.1016/j.mtcomm.2022.103214
K0.5La0.5SrMgW0.988O6Mn0.012	https://doi.org/10.1016/j.mtcomm.2022.103214
K0.5La0.5SrMgW0.982O6Mn0.018	https://doi.org/10.1016/j.mtcomm.2022.103214
K0.5La0.5SrMgW0.976O6Mn0.024	https://doi.org/10.1016/j.mtcomm.2022.103214

K0.5La0.5SrMgW0.97O6Mn0.03	https://doi.org/10.1016/j.mtcomm.2022.103214
BaLaMgTa0.994O6Mn0.006	https://doi.org/10.3390/ma16114029
BaLaMgTa0.992O6Mn0.008	https://doi.org/10.3390/ma16114029
BaLaMgTa0.99O6Mn0.010	https://doi.org/10.3390/ma16114029
BaLaMgTa0.988O6Mn0.012	https://doi.org/10.3390/ma16114029
BaLaMgTa0.996O6Mn0.004	https://doi.org/10.3390/ma16114029
BaLaMgTa0.998O6Mn0.002	https://doi.org/10.3390/ma16114029
KRb3Ge20.99F12Mn0.01	https://doi.org/10.1016/j.ceramint.2021.10.212
KRb3Ge20.9975F12Mn0.0025	https://doi.org/10.1016/j.ceramint.2021.10.212
KRb3Ge20.995F12Mn0.005	https://doi.org/10.1016/j.ceramint.2021.10.212
KRb3Ge20.98F12Mn0.02	https://doi.org/10.1016/j.ceramint.2021.10.212
KRb3Ge20.97F12Mn0.03	https://doi.org/10.1016/j.ceramint.2021.10.212
KRb3Ge20.96F12Mn0.04	https://doi.org/10.1016/j.ceramint.2021.10.212
KRb3Ge20.95F12Mn0.05	https://doi.org/10.1016/j.ceramint.2021.10.212
KRb3Ge20.94F12Mn0.06	https://doi.org/10.1016/j.ceramint.2021.10.212
KRb3Ge20.93F12Mn0.07	https://doi.org/10.1016/j.ceramint.2021.10.212
KRb3Ge20.92F12Mn0.08	https://doi.org/10.1016/j.ceramint.2021.10.212
Na2Si0.97F6Mn0.03	https://doi.org/10.1016/j.jlumin.2023.119684
Na2Si0.95F6Mn0.05	https://doi.org/10.1016/j.jlumin.2023.119684
Na2Si0.93F6Mn0.07	https://doi.org/10.1016/j.jlumin.2023.119684
Na2Si0.91F6Mn0.09	https://doi.org/10.1016/j.jlumin.2023.119684
Na2Si0.99F6Mn0.01	https://doi.org/10.1016/j.jlumin.2023.119684
N2C8H24Zr0.9929F6Mn0.0071	https://doi.org/10.3390/ma15196543
Ba2LaNb0.995O6Mn0.005	https://doi.org/10.1016/j.jallcom.2022.167435
Ba2LaNb0.8Zr0.995W0.1O6Mn0.005	https://doi.org/10.1016/j.jallcom.2022.167435
Ba2LaZr0.5W0.995O6Mn0.005	https://doi.org/10.1016/j.jallcom.2022.167435
Ba2LaNb0.994O6Mn0.006	https://doi.org/10.1016/j.jallcom.2022.167435
Ba2LaNb1O6Mn0.000	https://doi.org/10.1016/j.jallcom.2022.167435
Ba2LaNb0.998O6Mn0.002	https://doi.org/10.1016/j.jallcom.2022.167435
Ba2LaNb0.996O6Mn0.004	https://doi.org/10.1016/j.jallcom.2022.167435
Ba2LaNb0.982O6Mn0.018	https://doi.org/10.1016/j.jallcom.2022.167435

Ba2LaNb0.9995O6Mn0.0005	https://doi.org/10.1016/j.jallcom.2022.167435
Cs3V20.9985O2F7Mn0.0015	https://doi.org/10.1016/j.materresbull.2022.111798
Cs3V20.998O2F7Mn0.002	https://doi.org/10.1016/j.materresbull.2022.111798
Cs3V20.9975O2F7Mn0.0025	https://doi.org/10.1016/j.materresbull.2022.111798
Cs3V20.999O2F7Mn0.001	https://doi.org/10.1016/j.materresbull.2022.111798
Cs3V20.9995O2F7Mn0.0005	https://doi.org/10.1016/j.materresbull.2022.111798
Ba2CaW0.997O6Mn0.003	https://doi.org/10.1016/j.materresbull.2022.111798
Ba2CaW0.999O6Mn0.001	https://doi.org/10.1016/j.materresbull.2022.111798
Ba2CaW0.998O6Mn0.002	https://doi.org/10.1016/j.materresbull.2022.111798
Ba2CaW.995O6Mn0.005	https://doi.org/10.1016/j.materresbull.2022.111798
Ba2CaW0.993O6Mn0.007	https://doi.org/10.1016/j.materresbull.2022.111798
Ba2CaW0.988O6Mn0.012	https://doi.org/10.1016/j.materresbull.2022.111798
K5In30.9951F14Mn0.0059	https://doi.org/10.1016/j.optmat.2022.112223%E2%80%9D
K5In30.9965F14Mn0.0035	https://doi.org/10.1016/j.optmat.2022.112223%E2%80%9D
K5In30.9874F14Mn0.0126	https://doi.org/10.1016/j.optmat.2022.112223%E2%80%9D
K5In30.912F14Mn0.0188	https://doi.org/10.1016/j.optmat.2022.112223%E2%80%9D
K5In30.98F14Mn0.02	https://doi.org/10.1016/j.optmat.2022.112223%E2%80%9D
Rb2KSc0.9751F6Mn0.0249	https://doi.org/10.1016/j.jlumin.2022.119531
Rb2KSc0.9851F6Mn0.0149	https://doi.org/10.1016/j.jlumin.2022.119531
Rb2KSc0.9822F6Mn0.0178	https://doi.org/10.1016/j.jlumin.2022.119531
Rb2KSc0.9704F6Mn0.0296	https://doi.org/10.1016/j.jlumin.2022.119531
Rb2KSc0.9659F6Mn0.0341	https://doi.org/10.1016/j.jlumin.2022.119531
BaTi0.9997F6Mn0.0003	https://doi.org/10.1016/j.optmat.2023.114129
BaTi0.9995F6Mn0.0005	https://doi.org/10.1016/j.optmat.2023.114129
BaTi0.9993F6Mn0.0007	https://doi.org/10.1016/j.optmat.2023.114129
BaTi0.9991F6Mn0.0009	https://doi.org/10.1016/j.optmat.2023.114129
BaTi0.9999F6Mn0.0001	https://doi.org/10.1016/j.optmat.2023.114129
K5In30.9941F14Mn0.0059	https://doi.org/10.1016/j.optmat.2022.112223
K5In30.9965F14Mn0.0035	https://doi.org/10.1016/j.optmat.2022.112223
K5In30.9874F14Mn0.0126	https://doi.org/10.1016/j.optmat.2022.112223
K5In30.9812F14Mn0.0188	https://doi.org/10.1016/j.optmat.2022.112223

K5In30.98F14Mn0.02	https://doi.org/10.1016/j.optmat.2022.112223
CoSi0.945F6?6H2OMn0.055	https://doi.org/10.1016/j.optmat.2022.112223
CsNaGe0.92Ti0.5F6Mn0.08	https://doi.org/10.1016/j.optmat.2022.112223
CsNaGe0.995Ti0.5F6Mn0.005	https://doi.org/10.1016/j.optmat.2022.112223
CsNaGe0.99Ti0.5F6Mn0.01	https://doi.org/10.1016/j.optmat.2022.112223
CsNaGe0.98Ti0.5F6Mn0.02	https://doi.org/10.1016/j.optmat.2022.112223
CsNaGe0.96Ti0.5F6Mn0.04	https://doi.org/10.1016/j.optmat.2022.112223
CsNaGe0.88Ti0.5F6Mn0.12	https://doi.org/10.1016/j.optmat.2022.112223
CsNaGe0.84Ti0.5F6Mn0.16	https://doi.org/10.1016/j.optmat.2022.112223
Ca2YTa0.994O6Mn0.006	https://doi.org/10.1016/j.jlumin.2022.119458
Ca2YTa0.998O6Mn0.002	https://doi.org/10.1016/j.jlumin.2022.119458
Ca2YTa0.996O6Mn0.004	https://doi.org/10.1016/j.jlumin.2022.119458
Ca2YTa0.992O6Mn0.008	https://doi.org/10.1016/j.jlumin.2022.119458
Ca2YTa0.99O6Mn0.010	https://doi.org/10.1016/j.jlumin.2022.119458
Ca2YSb0.994O6Mn0.006	https://doi.org/10.1016/j.jlumin.2022.119458
Ca2YSb0.992O6Mn0.008	https://doi.org/10.1016/j.jlumin.2022.119458
Ca2YSb0.998O6Mn0.002	https://doi.org/10.1016/j.jlumin.2022.119458
Ca2YSb0.996O6Mn0.004	https://doi.org/10.1016/j.jlumin.2022.119458
Ca2YSb0.99O6Mn0.010	https://doi.org/10.1016/j.jlumin.2022.119458
Rb2KIn0.976F6Mn0.024	https://doi.org/10.1016/j.optmat.2022.112307
Rb2KIn0.992F6Mn0.008	https://doi.org/10.1016/j.optmat.2022.112307
Rb2KIn0.9908F6Mn0.0092	https://doi.org/10.1016/j.optmat.2022.112307
Rb2KIn0.9852F6Mn0.0148	https://doi.org/10.1016/j.optmat.2022.112307
Rb2KIn0.9565F6Mn0.0435	https://doi.org/10.1016/j.optmat.2022.112307
Rb2KIn0.9212F6Mn0.0798	https://doi.org/10.1016/j.optmat.2022.112307
Rb2KIn0.98614F6Mn0.01386	https://doi.org/10.1016/j.optmat.2022.112307
Gd2Zn0.8Mg0.2Ti0.998O6Mn0.002	https://doi.org/10.1016/j.jlumin.2022.118895
Gd2Zn0.8Mg0.2Ti0.995O6Mn0.005	https://doi.org/10.1016/j.jlumin.2022.118895
Gd2Zn0.8Mg0.2Ti0.99O6Mn0.01	https://doi.org/10.1016/j.jlumin.2022.118895
Gd2Zn0.8Mg0.2Ti0.98O6Mn0.02	https://doi.org/10.1016/j.jlumin.2022.118895
Gd2Zn0.8Mg0.2Ti0.97O6Mn0.03	https://doi.org/10.1016/j.jlumin.2022.118895

K2NaIn0.98F6Mn0.02	https://doi.org/10.1016/j.jlumin.2022.118895
K2NaIn0.99F6Mn0.01	https://doi.org/10.1016/j.jlumin.2022.118895
K2NaIn0.96F6Mn0.04	https://doi.org/10.1016/j.jlumin.2022.118895
K2NaIn0.92F6Mn0.08	https://doi.org/10.1016/j.jlumin.2022.118895
K2NaIn0.995F6Mn0.005	https://doi.org/10.1016/j.jlumin.2022.118895
K2NaIn0.9975F6Mn0.0025	https://doi.org/10.1016/j.jlumin.2022.118895
Na3Al0.999F6Mn0.001	https://doi.org/10.1016/j.omx.2023.100276
K5Nb30.995OF18Mn0.005	https://doi.org/10.1016/j.optmat.2022.113175
K5Nb30.9975OF18Mn0.0025	https://doi.org/10.1016/j.optmat.2022.113175
K5Nb30.9925OF18Mn0.0075	https://doi.org/10.1016/j.optmat.2022.113175
K5Nb30.99OF18Mn0.01	https://doi.org/10.1016/j.optmat.2022.113175
K5Nb30.9875OF18Mn0.0125	https://doi.org/10.1016/j.optmat.2022.113175
Rb2KTi0.995OF5Mn0.005	https://doi.org/10.1021/acsaom.2c00005.
LaAl0.999O3Mn0.001	https://doi.org/10.1039/d2qi01683d
LaAl0.998O3Mn0.002	https://doi.org/10.1039/d2qi01683d
LaAl0.9995O3Mn0.0005	https://doi.org/10.1039/d2qi01683d
K3Nb0.9993OF5(HF2)Mn0.0007	https://doi.org//abstract=4593152
K3Nb0.9991OF5(HF2)Mn0.0009	https://doi.org//abstract=4593152
K3Nb0.9989OF5(HF2)Mn0.0011	https://doi.org//abstract=4593152
K3Nb0.9987OF5(HF2)Mn0.0013	https://doi.org//abstract=4593152
K3Nb0.9995OF5(HF2)Mn0.0005	https://doi.org//abstract=4593152
K3Nb0.9997OF5(HF2)Mn0.0003	https://doi.org//abstract=4593152
Cs3RbGe20.9487F12Mn0.0513	https://doi.org//abstract=4613345
Cs3RbGe20.9885F12Mn0.0115	https://doi.org//abstract=4613345
Cs3RbGe20.9794F12Mn0.0206	https://doi.org//abstract=4613345
Cs3RbGe20.9277F12Mn0.0723	https://doi.org//abstract=4613345
Cs3RbGe20.9029F12Mn0.0971	https://doi.org//abstract=4613345
K2Ta0.98F7Mn0.02	https://doi.org//abstract=4580627
K2Ta0.995F7Mn0.005	https://doi.org//abstract=4580627
K2Ta0.99F7Mn0.01	https://doi.org//abstract=4580627
K2Ta0.97F7Mn0.03	https://doi.org//abstract=4580627

K2Ta0.96F7Mn0.04	https://doi.org//abstract=4580627
K2Ta0.95F7Mn0.05	https://doi.org//abstract=4580627
K2Si0.92F6Mn0.08	https://doi.org/10.1149/2.0051601jss
K2Si0.92F6Mn0.08	https://doi.org/10.1039/C8TC00133B
K2Si0.952F6Mn0.048	https://doi.org/10.1111/jace.16762
Na2Si0.968F6Mn0.0032	https://doi.org/10.1111/jace.16762
Cs2Si0.968F6Mn0.0032	https://doi.org/10.1111/jace.16762
K2Ti0.968F6Mn0.0032	https://doi.org/10.1111/jace.16762
K2Ge0.968F6Mn0.0032	https://doi.org/10.1111/jace.16762
Rb2Si0.938F6Mn0.062	https://doi.org/10.1111/jace.16762
Cs2Si0.8925F6Mn0.1075	https://doi.org/10.1021/acsphotonics.7b00852
Rb2Ti0.9F6Mn0.051	https://doi.org/10.1039/C8TC02615G
K2Ge0.8826F60.1174	https://doi.org/10.1021/acsami.8b03525
K2Ge0.9F6Mn0.1	https://doi.org/10.1021/acs.inorgchem.7b01890
Cs2Ge0.9F6Mn0.1	https://doi.org/10.1021/acs.inorgchem.7b01890
Na2Ge0.9F6Mn0.1	https://doi.org/10.1021/acs.inorgchem.7b01890
Rb2Ge0.992F6Mn0.008	https://doi.org/10.1021/acs.inorgchem.7b01890
K2Si0.996F6Mn0.004	https://doi.org/10.1016/j.jphotochem.2017.09.051
K2Si0.996F6Mn0.004	https://doi.org/10.1149/2.005204jes
K2Si0.98F6Mn0.02	https://doi.org/10.1149/2.005204jes
Ba2GdTa0.998O6Mn0.002	https://doi.org/10.1021/acsomega.9b01787
Ba2GdTa0.996O6Mn0.004	https://doi.org/10.1021/acsomega.9b01787
Ba2GdTa0.994O6Mn0.006	https://doi.org/10.1021/acsomega.9b01787
Ba2GdTa0.992O6Mn0.008	https://doi.org/10.1021/acsomega.9b01787
Ba2GdTa0.99O6Mn0.01	https://doi.org/10.1021/acsomega.9b01787
Ba2GdTa0.988O6Mn0.012	https://doi.org/10.1021/acsomega.9b01787
Ba2GdSb0.997O6Mn0.003	https://doi.org/10.1039/C8DT01575A
Ba2YNb0.995O6Mn0.005	https://doi.org/10.1016/j.optmat.2017.05.028
Ca2GdTa0.998O6Mn0.002	https://doi.org/10.1016/j.jlumin.2019.116525
Ca2GdTa0.996O6Mn0.004	https://doi.org/10.1016/j.jlumin.2019.116525
Ca2GdTa0.994O6Mn0.006	https://doi.org/10.1016/j.jlumin.2019.116525

Ca2GdTa0.992O6Mn0.008	https://doi.org/10.1016/j.jlumin.2019.116525
Ca2GdTa0.99O6Mn0.01	https://doi.org/10.1016/j.jlumin.2019.116525
Ca2GdTa0.988O6Mn0.012	https://doi.org/10.1016/j.jlumin.2019.116525
Ca2LaTa0.996OMn0.004	https://doi.org/10.1016/j.jallcom.2018.12.027
Ca2GdSb0.999O6Mn0.001	https://doi.org/10.1111/jace.16353
Ca2GdSb0.998O6Mn0.002	https://doi.org/10.1111/jace.16353
Ca2GdSb0.996O6Mn0.004	https://doi.org/10.1111/jace.16353
Ca2GdSb0.994O6Mn0.006	https://doi.org/10.1111/jace.16353
Ca2GdSb0.992O6Mn0.008	https://doi.org/10.1111/jace.16353
Ca2GdSb0.99O6Mn0.01	https://doi.org/10.1111/jace.16353
Ca2GdSb0.988O6Mn0.012	https://doi.org/10.1111/jace.16353
SrLaGa0.999O4Mn0.001	https://doi.org/10.1111/jace.16353
SrLaGa0.9985O4Mn0.0015	https://doi.org/10.1111/jace.16353
SrLaGa0.998O4Mn0.002	https://doi.org/10.1111/jace.16353
SrLaGa0.997O4Mn0.003	https://doi.org/10.1111/jace.16353
SrLaGa0.995O4Mn0.005	https://doi.org/10.1111/jace.16353
SrLaGa0.993O4Mn0.007	https://doi.org/10.1111/jace.16353
KGa0.99P2O7Mn0.01	https://doi.org/10.1016/j.jlumin.2019.116565
KGa0.97P2O7Mn0.03	https://doi.org/10.1016/j.jlumin.2019.116565
KGa0.95P2O7Mn0.05	https://doi.org/10.1016/j.jlumin.2019.116565
KGa0.93P2O7Mn0.07	https://doi.org/10.1016/j.jlumin.2019.116565
KGa0.91P2O7Mn0.09	https://doi.org/10.1016/j.jlumin.2019.116565
KGa0.89P2O7Mn0.11	https://doi.org/10.1016/j.jlumin.2019.116565
CaGdAl0.9995O4Mn0.0005	https://doi.org/10.1016/j.jlumin.2018.06.072
CaGdAl0.999O4Mn0.001	https://doi.org/10.1016/j.jlumin.2018.06.072
CaGdAl0.998O4Mn0.002	https://doi.org/10.1016/j.jlumin.2018.06.072
CaGdAl0.996O4Mn0.004	https://doi.org/10.1016/j.jlumin.2018.06.072
CaGdAl0.994O4Mn0.006	https://doi.org/10.1016/j.jlumin.2018.06.072
CaGdAl0.992O4Mn0.008	https://doi.org/10.1016/j.jlumin.2018.06.072
CaGdAl0.99O4Mn0.01	https://doi.org/10.1016/j.jlumin.2018.06.072
K3Sc0.9966F6Mn0.0034	https://doi.org/10.1021/acsami.8b01885

K3Sc0.9947F6Mn0.0053	https://doi.org/10.1021/acsami.8b01885
K3Sc0.9932F6Mn0.0068	https://doi.org/10.1021/acsami.8b01885
K3Sc0.9903F6Mn0.0097	https://doi.org/10.1021/acsami.8b01885
K3Sc0.9812F6Mn0.0188	https://doi.org/10.1021/acsami.8b01885
K3Sc0.9654F6Mn0.0346	https://doi.org/10.1021/acsami.8b01885
K3Sc0.9483F6Mn0.0517	https://doi.org/10.1021/acsami.8b01885
KLaMgW0.999O6Mn0.001	https://doi.org/10.1016/j.ceramint.2018.11.143
KLaMgW0.997O6Mn0.003	https://doi.org/10.1016/j.ceramint.2018.11.143
KLaMgW0.994O6Mn0.006	https://doi.org/10.1016/j.ceramint.2018.11.143
KLaMgW0.99O6Mn0.01	https://doi.org/10.1016/j.ceramint.2018.11.143
KLaMgW0.985O6Mn0.015	https://doi.org/10.1016/j.ceramint.2018.11.143
Li2ZnTi2.99O8Mn0.01	https://doi.org/10.1016/j.saa.2020.118567
Li2ZnTi2.96O8Mn0.04	https://doi.org/10.1016/j.saa.2020.118567
Li2ZnTi2.94O8Mn0.06	https://doi.org/10.1016/j.saa.2020.118567
Li2ZnTi2.92O8Mn0.08	https://doi.org/10.1016/j.saa.2020.118567
Li2ZnTi2.9O8Mn0.1	https://doi.org/10.1016/j.saa.2020.118567
Li2ZnTi2.8O8Mn0.2	https://doi.org/10.1016/j.saa.2020.118567
Li2ZnTi2.7O8Mn0.3	https://doi.org/10.1016/j.saa.2020.118567
Li2ZnTi2.6O8Mn0.4	https://doi.org/10.1016/j.saa.2020.118567
Li6CaLa2Sb1.9975O12Mn0.0025	https://doi.org/10.1016/j.jlumin.2020.117031
Li6CaLa2Sb1.9965O12Mn0.0035	https://doi.org/10.1016/j.jlumin.2020.117031
Li6CaLa2Sb1.9955O12Mn0.0045	https://doi.org/10.1016/j.jlumin.2020.117031
Li6CaLa2Sb1.995O12Mn0.005	https://doi.org/10.1016/j.jlumin.2020.117031
Li6CaLa2Sb1.9945O12Mn0.0055	https://doi.org/10.1016/j.jlumin.2020.117031
Li6CaLa2Sb1.9936O12Mn0.0065	https://doi.org/10.1016/j.jlumin.2020.117031
CaMg2La2W1.996O12Mn0.004	https://doi.org/10.1016/j.mtchem.2021.100512
CaMg2La2W1.994O12Mn0.006	https://doi.org/10.1016/j.mtchem.2021.100512
CaMg2La2W1.992O12Mn0.008	https://doi.org/10.1016/j.mtchem.2021.100512
CaMg2La2W1.99O12Mn0.010	https://doi.org/10.1016/j.mtchem.2021.100512
CaMg2La2W1.988O12Mn0.012	https://doi.org/10.1016/j.mtchem.2021.100512
CaMg2La2W1.985O12Mn0.015	https://doi.org/10.1016/j.mtchem.2021.100512

CaMg2La2W1.98O12Mn0.02	https://doi.org/10.1016/j.mtchem.2021.100512
SrLaSc0.995O4Mn0.005	https://doi.org/10.1016/j.dyepig.2018.01.052
SrLaSc0.992O4Mn0.008	https://doi.org/10.1016/j.dyepig.2018.01.052
SrLaSc0.988O4Mn0.012	https://doi.org/10.1016/j.dyepig.2018.01.052
SrLaSc0.985O4Mn0.015	https://doi.org/10.1016/j.dyepig.2018.01.052
SrLaSc0.98O4Mn0.02	https://doi.org/10.1016/j.dyepig.2018.01.052
Li3Mg2Ta0.999O6Mn0.001	https://doi.org/10.1016/j.inoche.2020.107903
Li3Mg2Ta0.997O6Mn0.003	https://doi.org/10.1016/j.inoche.2020.107903
Li3Mg2Ta0.995O6Mn0.005	https://doi.org/10.1016/j.inoche.2020.107903
Li3Mg2Ta0.993O6Mn0.007	https://doi.org/10.1016/j.inoche.2020.107903
Li3Mg2Ta0.991O6Mn0.009	https://doi.org/10.1016/j.inoche.2020.107903
Li3Mg2Ta0.989O6Mn0.011	https://doi.org/10.1016/j.inoche.2020.107903
Sr2LuTa0.999O6Mn0.001	https://doi.org/10.1016/j.jlumin.2021.117901
Sr2LuTa0.998O6Mn0.002	https://doi.org/10.1016/j.jlumin.2021.117901
Sr2LuTa0.996O6Mn0.004	https://doi.org/10.1016/j.jlumin.2021.117901
Sr2LuTa0.994O6Mn0.006	https://doi.org/10.1016/j.jlumin.2021.117901
Sr2LuTa0.99O6Mn0.01	https://doi.org/10.1016/j.jlumin.2021.117901
Sr2LuTa0.988O6Mn0.012	https://doi.org/10.1016/j.jlumin.2021.117901
Sr2LuTa0.985O6Mn0.015	https://doi.org/10.1016/j.jlumin.2021.117901
Sr2LuTa0.98O6Mn0.02	https://doi.org/10.1016/j.jlumin.2021.117901
Ca2YTa0.999O6Mn0.001	https://doi.org/10.1016/j.optlastec.2020.106349
Ca2YTa0.998O6Mn0.002	https://doi.org/10.1016/j.optlastec.2020.106349
Ca2YTa0.996O6Mn0.004	https://doi.org/10.1016/j.optlastec.2020.106349
Ca2YTa0.994O6Mn0.006	https://doi.org/10.1016/j.optlastec.2020.106349
Ca2YTa0.992O6Mn0.008	https://doi.org/10.1016/j.optlastec.2020.106349
Ca2YTa0.99O6Mn0.01	https://doi.org/10.1016/j.optlastec.2020.106349
Li2ZnSn1.998O6Mn0.002	https://doi.org/10.1016/j.jlumin.2018.01.023
Li2ZnSn1.996O6Mn0.004	https://doi.org/10.1016/j.jlumin.2018.01.023
Li2ZnSn1.994O6Mn0.006	https://doi.org/10.1016/j.jlumin.2018.01.023
Li2ZnSn1.992O6Mn0.008	https://doi.org/10.1016/j.jlumin.2018.01.023
Li2ZnSn1.99O6Mn0.01	https://doi.org/10.1016/j.jlumin.2018.01.023

Li5La3Ta1.998O12Mn0.002	https://doi.org/10.1111/jace.16447
Li5La3Ta1.996O12Mn0.004	https://doi.org/10.1111/jace.16447
Li5La3Ta1.994O12Mn0.006	https://doi.org/10.1111/jace.16447
Li5La3Ta1.992O12Mn0.008	https://doi.org/10.1111/jace.16447
Li5La3Ta1.99O12Mn0.01	https://doi.org/10.1111/jace.16447
CaY0.5Ta0.498O3Mn0.002	https://doi.org/10.1016/j.jallcom.2020.156875
CaY0.5Ta0.4975O3Mn0.0025	https://doi.org/10.1016/j.jallcom.2020.156875
CaY0.5Ta0.497O3Mn0.003	https://doi.org/10.1016/j.jallcom.2020.156875
CaY0.5Ta0.495O3Mn0.005	https://doi.org/10.1016/j.jallcom.2020.156875
CaY0.5Ta0.492O3Mn0.008	https://doi.org/10.1016/j.jallcom.2020.156875
CaY0.5Ta0.49O3Mn0.010	https://doi.org/10.1016/j.jallcom.2020.156875
CaY0.5Ta0.48O3Mn0.02	https://doi.org/10.1016/j.jallcom.2020.156875
CaY0.5Ta0.47O3Mn0.03	https://doi.org/10.1016/j.jallcom.2020.156875
CaY0.5Ta0.45O3Mn0.05	https://doi.org/10.1016/j.jallcom.2020.156875
Mg2La3Nb0.998O9Mn0.002	https://doi.org/10.1016/j.apt.2020.08.009
Mg2La3Nb0.996O9Mn0.004	https://doi.org/10.1016/j.apt.2020.08.009
Mg2La3Nb0.994O9Mn0.006	https://doi.org/10.1016/j.apt.2020.08.009
Mg2La3Nb0.992O9Mn0.008	https://doi.org/10.1016/j.apt.2020.08.009
Mg2La3Nb0.99O9Mn0.01	https://doi.org/10.1016/j.apt.2020.08.009
Sr2MgGe2O7	https://doi.org/10.1016/j.jallcom.2018.05.264
Sr2MgGe1.999O7Mn0.00	https://doi.org/10.1016/j.jallcom.2018.05.264
Sr2MgGe1.997O7Mn0.003	https://doi.org/10.1016/j.jallcom.2018.05.264
Sr2MgGe1.995O7Mn0.005	https://doi.org/10.1016/j.jallcom.2018.05.264
Sr2MgGe1.993O7Mn0.007	https://doi.org/10.1016/j.jallcom.2018.05.264
Sr2MgGe1.991O7Mn0.009	https://doi.org/10.1016/j.jallcom.2018.05.264
Sr2MgGe1.989O7Mn0.011	https://doi.org/10.1016/j.jallcom.2018.05.264
Sr2MgGe1.98O7Mn0.02	https://doi.org/10.1016/j.jallcom.2018.05.264
Ca2LaSb0.998O6Mn0.002	https://doi.org/10.1016/j.ceramint.2018.11.166
Ca2LaSb0.997O6Mn0.003	https://doi.org/10.1016/j.ceramint.2018.11.166
Ca2LaSb0.996O6Mn0.004	https://doi.org/10.1016/j.ceramint.2018.11.166
Ca2LaSb0.995O6Mn0.005	https://doi.org/10.1016/j.ceramint.2018.11.166

Ca2LaSb0.994O6Mn0.006	https://doi.org/10.1016/j.ceramint.2018.11.166
Ca2LaSb0.992O6Mn0.008	https://doi.org/10.1016/j.ceramint.2018.11.166
LaSrZnNb0.998O6Mn0.002	https://doi.org/10.1016/j.optmat.2021.111063
LaSrZnNb0.997O6Mn0.003	https://doi.org/10.1016/j.optmat.2021.111063
LaSrZnNb0.996O6Mn0.004	https://doi.org/10.1016/j.optmat.2021.111063
LaSrZnNb0.995O6Mn0.005	https://doi.org/10.1016/j.optmat.2021.111063
LaSrZnNb0.994O6Mn0.006	https://doi.org/10.1016/j.optmat.2021.111063
LaSrZnNb0.993O6Mn0.007	https://doi.org/10.1016/j.optmat.2021.111063
LaSrZnNb0.992O6Mn0.008	https://doi.org/10.1016/j.optmat.2021.111063
Sr2GdNb0.9985O6Mn0.0015	https://doi.org/10.1016/j.jlumin.2019.116968
Sr2GdNb0.998O6Mn0.002	https://doi.org/10.1016/j.jlumin.2019.116968
Sr2GdNb0.9975O6Mn0.0025	https://doi.org/10.1016/j.jlumin.2019.116968
Sr2GdNb0.997O6Mn0.003	https://doi.org/10.1016/j.jlumin.2019.116968
Sr2GdNb0.996O6Mn0.004	https://doi.org/10.1016/j.jlumin.2019.116968
Sr2GdNb0.995O6Mn0.005	https://doi.org/10.1016/j.jlumin.2019.116968
Sr2GdNb0.9935O6Mn0.0065	https://doi.org/10.1016/j.jlumin.2019.116968
Ba2LuTa0.998O6Mn0.002	https://doi.org/10.1016/j.materresbull.2021.111301
Ba2LuTa0.997O6Mn0.003	https://doi.org/10.1016/j.materresbull.2021.111301
Ba2LuTa0.996O6Mn0.004	https://doi.org/10.1016/j.materresbull.2021.111301
Ba2LuTa0.995O6Mn0.005	https://doi.org/10.1016/j.materresbull.2021.111301
Ba2LuTa0.994O6Mn0.006	https://doi.org/10.1016/j.materresbull.2021.111301
Ba2LuTa0.993O6Mn0.007	https://doi.org/10.1016/j.materresbull.2021.111301
Ba2LuTa0.992O6Mn0.008	https://doi.org/10.1016/j.materresbull.2021.111301
Na4Mg0.995(WO4)3Mn0.005	https://doi.org/10.1021/acs.chemmater.9b03886
Na4Mg0.99(WO4)3Mn0.01	https://doi.org/10.1021/acs.chemmater.9b03886
Na4Mg0.98(WO4)3Mn0.02	https://doi.org/10.1021/acs.chemmater.9b03886
Na4Mg0.97(WO4)3Mn0.03	https://doi.org/10.1021/acs.chemmater.9b03886
Na4Mg0.96(WO4)3Mn0.04	https://doi.org/10.1021/acs.chemmater.9b03886
Na4Mg0.95(WO4)3Mn0.05	https://doi.org/10.1021/acs.chemmater.9b03886
Na4Mg0.94(WO4)3Mn0.06	https://doi.org/10.1021/acs.chemmater.9b03886
Na4Mg0.93(WO4)3Mn0.07	https://doi.org/10.1021/acs.chemmater.9b03886

Ca3La2W1.998O12Mn0.002	https://doi.org/10.1016/j.dyepig.2018.01.022
Ca3La2W1.996O12Mn0.004	https://doi.org/10.1016/j.dyepig.2018.01.022
Ca3La2W1.992O12Mn0.008	https://doi.org/10.1016/j.dyepig.2018.01.022
Ca3La2W1.99O12Mn0.01	https://doi.org/10.1016/j.dyepig.2018.01.022
Ca3La2W1.988O12Mn0.012	https://doi.org/10.1016/j.dyepig.2018.01.022
Ca3La2W1.985O12Mn0.015	https://doi.org/10.1016/j.dyepig.2018.01.022
Ca3La2W1.98O12Mn0.02	https://doi.org/10.1016/j.dyepig.2018.01.022
Ba2LaSb0.997O6Mn0.003	https://doi.org/10.1016/j.jlumin.2018.09.059
Ba2LaSb0.995O6Mn0.005	https://doi.org/10.1016/j.jlumin.2018.09.059
Ba2LaSb0.993O6Mn0.007	https://doi.org/10.1016/j.jlumin.2018.09.059
Ba2LaSb0.99O6Mn0.010	https://doi.org/10.1016/j.jlumin.2018.09.059
Ba2LaSb0.987O6Mn0.013	https://doi.org/10.1016/j.jlumin.2018.09.059
Ba2LaSb0.985O6Mn0.015	https://doi.org/10.1016/j.jlumin.2018.09.059
Ba2LaSb0.983O6Mn0.017	https://doi.org/10.1016/j.jlumin.2018.09.059
Ba2LaSb0.98O6Mn0.02	https://doi.org/10.1016/j.jlumin.2018.09.059
La2ZnTi0.9995O6Mn0.0005	https://doi.org/10.1016/j.optmat.2020.109980
La2ZnTi0.999O6Mn0.001	https://doi.org/10.1016/j.optmat.2020.109980
La2ZnTi0.998O6Mn0.002	https://doi.org/10.1016/j.optmat.2020.109980
La2ZnTi0.996O6Mn0.004	https://doi.org/10.1016/j.optmat.2020.109980
La2ZnTi0.994O6Mn0.006	https://doi.org/10.1016/j.optmat.2020.109980
Na3Ga0.995F6Mn0.005	https://doi.org/10.1016/j.jlumin.2019.116960
Na3Ga0.99F6Mn0.01	https://doi.org/10.1016/j.jlumin.2019.116960
Na3Ga0.97F6Mn0.03	https://doi.org/10.1016/j.jlumin.2019.116960
Na3Ga0.95F6Mn0.05	https://doi.org/10.1016/j.jlumin.2019.116960
Na3Ga0.93F6Mn0.07	https://doi.org/10.1016/j.jlumin.2019.116960
NaLaMgW0.999O6Mn0.001	https://doi.org/10.1364/OL.43.003305
NaLaMgW0.997O6Mn0.003	https://doi.org/10.1364/OL.43.003305
NaLaMgW0.995O6Mn0.005	https://doi.org/10.1364/OL.43.003305
NaLaMgW0.993O6Mn0.007	https://doi.org/10.1364/OL.43.003305
NaLaMgW0.991O6Mn0.009	https://doi.org/10.1364/OL.43.003305
NaLaMgW0.988O6Mn0.012	https://doi.org/10.1364/OL.43.003305

NaLaMgW0.985O6Mn0.015	https://doi.org/10.1364/OL.43.003305
LiLa2Nb0.999O6Mn0.001	https://doi.org/10.1016/j.jallcom.2018.04.295
LiLa2Nb0.997O6Mn0.003	https://doi.org/10.1016/j.jallcom.2018.04.295
LiLa2Nb0.995O6Mn0.005	https://doi.org/10.1016/j.jallcom.2018.04.295
LiLa2Nb0.993O6Mn0.007	https://doi.org/10.1016/j.jallcom.2018.04.295
LiLa2Nb0.99O6Mn0.01	https://doi.org/10.1016/j.jallcom.2018.04.295
K2LiAl0.99F6Mn0.01	https://doi.org/10.1016/j.optmat.2021.111392
K2LiAl0.97F6Mn0.03	https://doi.org/10.1016/j.optmat.2021.111392
K2LiAl0.95F6Mn0.05	https://doi.org/10.1016/j.optmat.2021.111392
K2LiAl0.93F6Mn0.07	https://doi.org/10.1016/j.optmat.2021.111392
K2LiAl0.91F6Mn0.09	https://doi.org/10.1016/j.optmat.2021.111392
Ba2MgGe1.998O7Mn0.002	https://doi.org/10.1016/j.jlumin.2018.06.061
Ba2MgGe1.992O7Mn0.008	https://doi.org/10.1016/j.jlumin.2018.06.061
Ba2MgGe1.987O7Mn0.013	https://doi.org/10.1016/j.jlumin.2018.06.061
Ba2MgGe1.998O7Mn0.02	https://doi.org/10.1016/j.jlumin.2018.06.061
LiAl3.9995O6FMn0.0005	https://doi.org/10.1016/j.jallcom.2018.09.199
LiAl3.999O6FMn0.001	https://doi.org/10.1016/j.jallcom.2018.09.199
LiAl3.998O6FMn0.002	https://doi.org/10.1016/j.jallcom.2018.09.199
LiAl3.996O6FMn0.004	https://doi.org/10.1016/j.jallcom.2018.09.199
LiAl3.994O6FMn0.006	https://doi.org/10.1016/j.jallcom.2018.09.199
LiAl3.992O6FMn0.008	https://doi.org/10.1016/j.jallcom.2018.09.199
LiAl3.99O6FMn0.01	https://doi.org/10.1016/j.jallcom.2018.09.199
K2Nb0.9953F7Mn0.0047	https://doi.org/10.1016/j.optmat.2018.10.031
K2Nb0.9909F7Mn0.0091	https://doi.org/10.1016/j.optmat.2018.10.031
K2Nb0.9867F7Mn0.0139	https://doi.org/10.1016/j.optmat.2018.10.031
K2Nb0.9753F7Mn0.0247	https://doi.org/10.1016/j.optmat.2018.10.031
K2Nb0.9587F7Mn0.0413	https://doi.org/10.1016/j.optmat.2018.10.031
K2Nb0.9468F7Mn0.0532	https://doi.org/10.1016/j.optmat.2018.10.031
Rb1.99MoO2F4Mn0.01	https://doi.org/10.1016/j.cej.2021.128974
Rb1.97MoO2F4Mn0.03	https://doi.org/10.1016/j.cej.2021.128974
Rb1.95MoO2F4Mn0.05	https://doi.org/10.1016/j.cej.2021.128974

Rb1.93MoO2F4Mn0.07	https://doi.org/10.1016/j.cej.2021.128974
Rb1.9MoO2F4Mn0.1	https://doi.org/10.1016/j.cej.2021.128974
Rb1.85MoO2F4Mn0.15	https://doi.org/10.1016/j.cej.2021.128974
Mg2InSb0.999O6Mn0.001	https://doi.org/10.1016/j.ceramint.2021.03.217
Mg2InSb0.998O6Mn0.002	https://doi.org/10.1016/j.ceramint.2021.03.217
Mg2InSb0.997O6Mn0.003	https://doi.org/10.1016/j.ceramint.2021.03.217
Mg2InSb0.995O6Mn0.005	https://doi.org/10.1016/j.ceramint.2021.03.217
Mg2InSb0.992O6Mn0.008	https://doi.org/10.1016/j.ceramint.2021.03.217
Mg2InSb0.99O6Mn0.01	https://doi.org/10.1016/j.ceramint.2021.03.217
K2Ti0.95F6Mn0.05	https://doi.org/10.1016/j.cej.2020.127161
K2Ti0.94F6Mn0.06	https://doi.org/10.1016/j.cej.2020.127161
K2Ti0.93F6Mn0.07	https://doi.org/10.1016/j.cej.2020.127161
K2Ti0.92F6Mn0.08	https://doi.org/10.1016/j.cej.2020.127161
K2Ti0.91F6Mn0.09	https://doi.org/10.1016/j.cej.2020.127161
K2Ti0.9F6Mn0.10	https://doi.org/10.1016/j.cej.2020.127161
Ca2ScTa0.9995O6Mn0.0005	https://doi.org/10.1016/j.optmat.2020.110274
Ca2ScTa0.999O6Mn0.001	https://doi.org/10.1016/j.optmat.2020.110274
Ca2ScTa0.998O6Mn0.002	https://doi.org/10.1016/j.optmat.2020.110274
Ca2ScTa0.996O6Mn0.004	https://doi.org/10.1016/j.optmat.2020.110274
Ca2ScTa0.994O6Mn0.006	https://doi.org/10.1016/j.optmat.2020.110274
Ca2ScTa0.992O6Mn0.008	https://doi.org/10.1016/j.optmat.2020.110274
Ca2ScTa0.99O6Mn0.01	https://doi.org/10.1016/j.optmat.2020.110274
Li4Ti4.999O12Mn0.001	https://doi.org/10.1016/j.jlumin.2020.117646
Li4Ti4.9975O12Mn0.0025	https://doi.org/10.1016/j.jlumin.2020.117646
Li4Ti4.99O12Mn0.01	https://doi.org/10.1016/j.jlumin.2020.117646
Li4Ti4.98O12Mn0.02	https://doi.org/10.1016/j.jlumin.2020.117646
Li4Ti4.97O12Mn0.03	https://doi.org/10.1016/j.jlumin.2020.117646
Li4Ti4.96O12Mn0.04	https://doi.org/10.1016/j.jlumin.2020.117646
Li4Ti4.95O12Mn0.05	https://doi.org/10.1016/j.jlumin.2020.117646
Li4Ti4.94O12Mn0.06	https://doi.org/10.1016/j.jlumin.2020.117646
Li4Ti4.93O12Mn0.07	https://doi.org/10.1016/j.jlumin.2020.117646

Li4Ti4.92O12Mn0.08	https://doi.org/10.1016/j.jlumin.2020.117646
SrLaZnSb0.999O6Mn0.001	https://doi.org/10.1016/j.jlumin.2020.117646
SrLaZnSb0.997O6Mn0.003	https://doi.org/10.1016/j.jphotochem.2021.113166
SrLaZnSb0.995O6Mn0.005	https://doi.org/10.1016/j.jphotochem.2021.113166
SrLaZnSb0.993O6Mn0.007	https://doi.org/10.1016/j.jphotochem.2021.113166
SrLaZnSb0.989O6Mn0.011	https://doi.org/10.1016/j.jphotochem.2021.113166
SrLaZnSb0.987O6Mn0.013	https://doi.org/10.1016/j.jphotochem.2021.113166
Ba2LaNb0.999O6Mn0.001	https://doi.org/10.1016/j.ceramint.2019.12.253
Ba2LaNb0.975O6Mn0.0025	https://doi.org/10.1016/j.ceramint.2019.12.253
Ba2LaNb0.995O6Mn0.005	https://doi.org/10.1016/j.ceramint.2019.12.253
Ba2LaNb0.925O6Mn0.0075	https://doi.org/10.1016/j.ceramint.2019.12.253
Ba2LaNb0.99O6Mn0.01	https://doi.org/10.1016/j.ceramint.2019.12.253
Ba2LaNb0.98O6Mn0.02	https://doi.org/10.1016/j.ceramint.2019.12.253
Li5La3Nb1.9995O12Mn0.0005	https://doi.org/10.1016/j.jlumin.2019.116888
Li5La3Nb1.999O12Mn0.0010	https://doi.org/10.1016/j.jlumin.2019.116888
Li5La3Nb1.998O12Mn0.002	https://doi.org/10.1016/j.jlumin.2019.116888
Li5La3Nb1.997O12Mn0.003	https://doi.org/10.1016/j.jlumin.2019.116888
Li5La3Nb1.996O12Mn0.004	https://doi.org/10.1016/j.jlumin.2019.116888
Li5La3Nb1.994O12Mn0.006	https://doi.org/10.1016/j.jlumin.2019.116888
LaTiSb0.997O6Mn0.003	https://doi.org/10.1016/j.jlumin.2021.118100
LaTiSb0.993O6Mn0.007	https://doi.org/10.1016/j.jlumin.2021.118100
LaTiSb0.99O6Mn0.01	https://doi.org/10.1016/j.jlumin.2021.118100
LaTiSb0.987O6Mn0.013	https://doi.org/10.1016/j.jlumin.2021.118100
LaTiSb0.984O6Mn0.016	https://doi.org/10.1016/j.jlumin.2021.118100
LaTiSb0.999O6Mn0.001	https://doi.org/10.1016/j.jlumin.2021.118100
Ba2YTa0.999O6Mn0.001	https://doi.org/10.1016/j.jlumin.2020.117621
Ba2YTa0.998O6Mn0.002	https://doi.org/10.1016/j.jlumin.2020.117621
Ba2YTa0.996O6Mn0.004	https://doi.org/10.1016/j.jlumin.2020.117621
Ba2YTa0.994O6Mn0.006	https://doi.org/10.1016/j.jlumin.2020.117621
Ba2YTa0.992O6Mn0.008	https://doi.org/10.1016/j.jlumin.2020.117621
Ba2YTa0.99O6Mn0.01	https://doi.org/10.1016/j.jlumin.2020.117621

K2LiGa0.99F6Mn0.01	https://doi.org/10.1016/j.jallcom.2017.12.184
K2LiGa0.975F6Mn0.025	https://doi.org/10.1016/j.jallcom.2017.12.184
K2LiGa0.95F6Mn0.05	https://doi.org/10.1016/j.jallcom.2017.12.184
K2LiGa0.9F6Mn0.1	https://doi.org/10.1016/j.jallcom.2017.12.184
K2LiGa0.8F6Mn0.2	https://doi.org/10.1016/j.jallcom.2017.12.184
BaSn0.98(PO4)2Mn0.02	https://doi.org/10.1016/j.molstruc.2020.129839
BaSn0.96(PO4)2Mn0.04	https://doi.org/10.1016/j.molstruc.2020.129839
BaSn0.94(PO4)2Mn0.06	https://doi.org/10.1016/j.molstruc.2020.129839
BaSn0.92(PO4)2Mn0.08	https://doi.org/10.1016/j.molstruc.2020.129839
BaSn0.9(PO4)2Mn0.1	https://doi.org/10.1016/j.molstruc.2020.129839
Sr3NaSb0.998O6Mn0.002	https://doi.org/10.1016/j.jlumin.2018.12.039
Sr3NaSb0.997O6Mn0.003	https://doi.org/10.1016/j.jlumin.2018.12.039
Sr3NaSb0.996O6Mn0.004	https://doi.org/10.1016/j.jlumin.2018.12.039
Sr3NaSb0.995O6Mn0.005	https://doi.org/10.1016/j.jlumin.2018.12.039
Sr3NaSb0.994O6Mn0.006	https://doi.org/10.1016/j.jlumin.2018.12.039
Sr3NaSb0.992O6Mn0.008	https://doi.org/10.1016/j.jlumin.2018.12.039
Sr3NaSb0.99O6Mn0.01	https://doi.org/10.1016/j.jlumin.2018.12.039
Na2Ti0.95F6Mn0.05	https://doi.org/10.1016/j.ceramint.2018.12.104
Na2Ti0.94F6Mn0.06	https://doi.org/10.1016/j.ceramint.2018.12.104
Na2Ti0.93F6Mn0.07	https://doi.org/10.1016/j.ceramint.2018.12.104
Na2Ti0.92F6Mn0.08	https://doi.org/10.1016/j.ceramint.2018.12.104
Na2Ti0.91F6Mn0.09	https://doi.org/10.1016/j.ceramint.2018.12.104
Na2Ti0.9F6Mn0.10	https://doi.org/10.1016/j.ceramint.2018.12.104
Na2Ti0.989F6Mn0.11	https://doi.org/10.1016/j.ceramint.2018.12.104
Li2Ge3.999O9Mn0.001	https://doi.org/10.1016/j.optmat.2019.109442
Li2Ge3.998O9Mn0.002	https://doi.org/10.1016/j.optmat.2019.109442
Li2Ge3.997O9Mn0.003	https://doi.org/10.1016/j.optmat.2019.109442
Li2Ge3.996O9Mn0.004	https://doi.org/10.1016/j.optmat.2019.109442
Li2Ge3.995O9Mn0.005	https://doi.org/10.1016/j.optmat.2019.109442
Ba2LaSb0.999O6Mn0.001	https://doi.org/10.1016/j.ceramint.2021.03.287
Ba2LaSb0.998O6Mn0.002	https://doi.org/10.1016/j.ceramint.2021.03.287

Ba2LaSb0.997O6Mn0.003	https://doi.org/10.1016/j.ceramint.2021.03.287
Ba2LaSb0.996O6Mn0.004	https://doi.org/10.1016/j.ceramint.2021.03.287
K2Nb0.99F7Mn0.01	https://doi.org/10.1016/j.jallcom.2020.158058
K2Nb0.98F7Mn0.02	https://doi.org/10.1016/j.jallcom.2020.158058
K2Nb0.96F7Mn0.04	https://doi.org/10.1016/j.jallcom.2020.158058
K2Nb0.95F7Mn0.05	https://doi.org/10.1016/j.jallcom.2020.158058
K2Nb0.94F7Mn0.06	https://doi.org/10.1016/j.jallcom.2020.158058
K2Nb0.92F7Mn0.08	https://doi.org/10.1016/j.jallcom.2020.158058
Mg2La2Sn0.999O7Mn0.001	https://doi.org/10.1016/j.materresbull.2018.08.037
Mg2La2Sn0.997O7Mn0.003	https://doi.org/10.1016/j.materresbull.2018.08.037
Mg2La2Sn0.994O7Mn0.006	https://doi.org/10.1016/j.materresbull.2018.08.037
Mg2La2Sn0.991O7Mn0.009	https://doi.org/10.1016/j.materresbull.2018.08.037
Mg2La2Sn0.988O7Mn0.012	https://doi.org/10.1016/j.materresbull.2018.08.037
Ba3Y2W0.999O9Mn0.001	https://doi.org/10.1016/j.jlumin.2020.117406
Ba3Y2W0.997O9Mn0.003	https://doi.org/10.1016/j.jlumin.2020.117406
Ba3Y2W0.995O9Mn0.005	https://doi.org/10.1016/j.jlumin.2020.117406
Ba3Y2W0.991O9Mn0.009	https://doi.org/10.1016/j.jlumin.2020.117406
Ba3Y2W0.987O9Mn0.013	https://doi.org/10.1016/j.jlumin.2020.117406
Mg2Al3.995Si5O18Mn0.005	https://doi.org/10.1016/j.dyepig.2017.08.050
Mg2Al3.99Si5O18Mn0.010	https://doi.org/10.1016/j.dyepig.2017.08.050
Mg2Al3.985Si5O18Mn0.015	https://doi.org/10.1016/j.dyepig.2017.08.050
Mg2Al3.98Si5O18Mn0.02	https://doi.org/10.1016/j.dyepig.2017.08.050
Mg2Al3.975Si5O18Mn0.025	https://doi.org/10.1016/j.dyepig.2017.08.050
Mg2Al3.97Si5O18Mn0.03	https://doi.org/10.1016/j.dyepig.2017.08.050
Lu3Al4.98O12Mn0.02	https://doi.org/10.1016/j.jeurceramsoc.2018.08.031
Lu3Al4.96O12Mn0.04	https://doi.org/10.1016/j.jeurceramsoc.2018.08.031
Lu3Al4.94O12Mn0.06	https://doi.org/10.1016/j.jeurceramsoc.2018.08.031
Lu3Al4.92O12Mn0.08	https://doi.org/10.1016/j.jeurceramsoc.2018.08.031
Rb2NaAl0.995F6Mn0.005	https://doi.org/10.1016/j.jlumin.2020.117491
Rb2NaAl0.99F6Mn0.010	https://doi.org/10.1016/j.jlumin.2020.117491
Rb2NaAl0.984F6Mn0.016	https://doi.org/10.1016/j.jlumin.2020.117491

Rb2NaAl0.96F6Mn0.04	https://doi.org/10.1016/j.jlumin.2020.117491
Rb2NaAl0.947F6Mn0.053	https://doi.org/10.1016/j.jlumin.2020.117491
Rb2NaAl0.94F6Mn0.06	https://doi.org/10.1016/j.jlumin.2020.117491
Li4Al0.999SbO6Mn0.001	https://doi.org/10.1016/j.ceramint.2021.06.185
Li4Al0.9985SbO6Mn0.0015	https://doi.org/10.1016/j.ceramint.2021.06.185
Li4Al0.998SbO6Mn0.002	https://doi.org/10.1016/j.ceramint.2021.06.185
Li4Al0.995SbO6Mn0.005	https://doi.org/10.1016/j.ceramint.2021.06.185
Li4Al0.99SbO6Mn0.01	https://doi.org/10.1016/j.ceramint.2021.06.185
Li4Al0.98SbO6Mn0.02	https://doi.org/10.1016/j.ceramint.2021.06.185
LiLaMgW0.999O6Mn0.001	https://doi.org/10.1039/C8RA05669B
LiLaMgW0.998O6Mn0.002	https://doi.org/10.1039/C8RA05669B
LiLaMgW0.996O6Mn0.004	https://doi.org/10.1039/C8RA05669B
LiLaMgW0.993O6Mn0.007	https://doi.org/10.1039/C8RA05669B
LiLaMgW0.99O6Mn0.01	https://doi.org/10.1039/C8RA05669B
LiLaMgW0.985O6Mn0.015	https://doi.org/10.1039/C8RA05669B
LiLaMgW0.98O6Mn0.02	https://doi.org/10.1039/C8RA05669B
Li6SrLa2Sb1.998O12Mn0.002	https://doi.org/10.1016/j.materresbull.2020.111040
Li6SrLa2Sb1.994O12Mn0.006	https://doi.org/10.1016/j.materresbull.2020.111040
Li6SrLa2Sb1.99O12Mn0.01	https://doi.org/10.1016/j.materresbull.2020.111040
Li6SrLa2Sb1.986O12Mn0.014	https://doi.org/10.1016/j.materresbull.2020.111040
Li6SrLa2Sb1.982O12Mn0.018	https://doi.org/10.1016/j.materresbull.2020.111040
Ca2GdNb0.998O6Mn0.002	https://doi.org/10.1016/j.spmi.2018.03.054
Ca2GdNb0.996O6Mn0.004	https://doi.org/10.1016/j.spmi.2018.03.054
Ca2GdNb0.994O6Mn0.006	https://doi.org/10.1016/j.spmi.2018.03.054
Ca2GdNb0.992O6Mn0.008	https://doi.org/10.1016/j.spmi.2018.03.054
Ca2GdNb0.99O6Mn0.01	https://doi.org/10.1016/j.spmi.2018.03.054
Ca2GdNb0.9987O6Mn0.013	https://doi.org/10.1016/j.spmi.2018.03.054
Ca2GdNb0.9984O6Mn0.016	https://doi.org/10.1016/j.spmi.2018.03.054
Ca2GdNb0.998O6Mn0.02	https://doi.org/10.1016/j.spmi.2018.03.054
SrLaAl0.999O4Mn0.001	https://doi.org/10.1039/C8RA06356G
SrLaAl0.998O4Mn0.002	https://doi.org/10.1039/C8RA06356G

SrLaAl0.996O4Mn0.004	https://doi.org/10.1039/C8RA06356G
SrLaAl0.994O4Mn0.006	https://doi.org/10.1039/C8RA06356G
SrLaAl0.992O4Mn0.008	https://doi.org/10.1039/C8RA06356G
SrLaAl0.99O4Mn0.01	https://doi.org/10.1039/C8RA06356G
Li3Mg2Sb0.995O6Mn0.005	https://doi.org/10.1016/j.jallcom.2018.09.162
Li3Mg2Sb0.99O6Mn0.01	https://doi.org/10.1016/j.jallcom.2018.09.162
Li3Mg2Sb0.985O6Mn0.0015	https://doi.org/10.1016/j.jallcom.2018.09.162
Li3Mg2Sb0.98O6Mn0.02	https://doi.org/10.1016/j.jallcom.2018.09.162
Li3Mg2Sb0.975O6Mn0.025	https://doi.org/10.1016/j.jallcom.2018.09.162
Li3Mg2Sb0.97O6Mn0.03	https://doi.org/10.1016/j.jallcom.2018.09.162
Li3Mg2Sb0.965O6Mn0.035	https://doi.org/10.1016/j.jallcom.2018.09.162
Li3Mg2Sb0.96O6Mn0.04	https://doi.org/10.1016/j.jallcom.2018.09.162
BaAl1.9995Ge2O8Mn0.0005	https://doi.org/10.1016/j.ijleo.2019.02.131
BaAl1.999Ge2O8Mn0.0010	https://doi.org/10.1016/j.ijleo.2019.02.131
BaAl1.997Ge2O8Mn0.003	https://doi.org/10.1016/j.ijleo.2019.02.131
BaAl1.995Ge2O8Mn0.005	https://doi.org/10.1016/j.ijleo.2019.02.131
BaAl1.993Ge2O8Mn0.007	https://doi.org/10.1016/j.ijleo.2019.02.131
BaAl1.99Ge2O8Mn0.01	https://doi.org/10.1016/j.ijleo.2019.02.131
BaAl1.98Ge2O8Mn0.02	https://doi.org/10.1016/j.ijleo.2019.02.131
K3Ta0.9427O2F4Mn0.0573	https://doi.org/10.1021/acs.inorgchem.8b03577
K3Ta0.9331O2F4Mn0.0669	https://doi.org/10.1021/acs.inorgchem.8b03577
K3Ta0.9318O2F4Mn0.0692	https://doi.org/10.1021/acs.inorgchem.8b03577
K3Ta0.9255O2F4Mn0.0745	https://doi.org/10.1021/acs.inorgchem.8b03577
K3Ta0.923O2F4Mn0.077	https://doi.org/10.1021/acs.inorgchem.8b03577
K3Ta0.9194O2F4Mn0.0806	https://doi.org/10.1021/acs.inorgchem.8b03577
CaMgLaSb0.998O6Mn0.002	https://doi.org/10.1016/j.inoche.2019.107607
CaMgLaSb0.997O6Mn0.003	https://doi.org/10.1016/j.inoche.2019.107607
CaMgLaSb0.996O6Mn0.004	https://doi.org/10.1016/j.inoche.2019.107607
CaMgLaSb0.994O6Mn0.006	https://doi.org/10.1016/j.inoche.2019.107607
CaMgLaSb0.992O6Mn0.008	https://doi.org/10.1016/j.inoche.2019.107607
CaMgLaSb0.991O6Mn0.009	https://doi.org/10.1016/j.inoche.2019.107607

SrLaLiTe0.995O6Mn0.005	https://doi.org/10.1111/jace.17910
SrLaLiTe0.99O6Mn0.01	https://doi.org/10.1111/jace.17910
SrLaLiTe0.98O6Mn0.02	https://doi.org/10.1111/jace.17910
SrLaLiTe0.97O6Mn0.03	https://doi.org/10.1111/jace.17910
SrLaLiTe0.96O6Mn0.04	https://doi.org/10.1111/jace.17910
SrLaLiTe0.95O6Mn0.05	https://doi.org/10.1111/jace.17910
SrLaLiTe0.94O6Mn0.06	https://doi.org/10.1111/jace.17910
SrLaLiTe0.93O6Mn0.07	https://doi.org/10.1111/jace.17910
SrLaLiTe0.92O6Mn0.08	https://doi.org/10.1111/jace.17910
Ba2LaSb0.999O6Mn0.001	https://doi.org/10.1016/j.jlumin.2019.01.014
Ba2LaSb0.998O6Mn0.002	https://doi.org/10.1016/j.jlumin.2019.01.014
Ba2LaSb0.996O6Mn0.004	https://doi.org/10.1016/j.jlumin.2019.01.014
Ba2LaSb0.994O6Mn0.006	https://doi.org/10.1016/j.jlumin.2019.01.014
Ba2LaSb0.992O6Mn0.008	https://doi.org/10.1016/j.jlumin.2019.01.014
Ba2LaSb0.99O6Mn0.01	https://doi.org/10.1016/j.jlumin.2019.01.014
SrGdAl0.9995O4Mn0.0005	https://doi.org/10.1039/C8RA08551J
SrGdAl0.999O4Mn0.0010	https://doi.org/10.1039/C8RA08551J
SrGdAl0.998O4Mn0.002	https://doi.org/10.1039/C8RA08551J
SrGdAl0.996O4Mn0.004	https://doi.org/10.1039/C8RA08551J
SrGdAl0.994O4Mn0.006	https://doi.org/10.1039/C8RA08551J
SrGdAl0.992O4Mn0.008	https://doi.org/10.1039/C8RA08551J
Ba2YTa0.998O6Mn0.002	https://doi.org/10.1016/j.inoche.2021.108568
Ba2YTa0.996O6Mn0.004	https://doi.org/10.1016/j.inoche.2021.108568
Ba2YTa0.994O6Mn0.006	https://doi.org/10.1016/j.inoche.2021.108568
Ba2YTa0.992O6Mn0.008	https://doi.org/10.1016/j.inoche.2021.108568
Ba2YTa0.99O6Mn0.01	https://doi.org/10.1016/j.inoche.2021.108568
Ba2YTa0.988O6Mn0.012	https://doi.org/10.1016/j.inoche.2021.108568
BaLaMgSb0.998O6Mn0.002	https://doi.org/10.1039/C8RA09928F
BaLaMgSb0.996O6Mn0.004	https://doi.org/10.1039/C8RA09928F
BaLaMgSb0.994O6Mn0.006	https://doi.org/10.1039/C8RA09928F
BaLaMgSb0.992O6Mn0.008	https://doi.org/10.1039/C8RA09928F

BaLaMgSb0.99O6Mn0.01	https://doi.org/10.1039/C8RA09928F
BaLaMgSb0.988O6Mn0.012	https://doi.org/10.1039/C8RA09928F
Ca2LaNb0.998O6Mn0.002	https://doi.org/10.1016/j.optlastec.2018.06.062
Ca2LaNb0.996O6Mn0.004	https://doi.org/10.1016/j.optlastec.2018.06.062
Ca2LaNb0.994O6Mn0.006	https://doi.org/10.1016/j.optlastec.2018.06.062
Ca2LaNb0.992O6Mn0.008	https://doi.org/10.1016/j.optlastec.2018.06.062
Ca2LaNb0.99O6Mn0.01	https://doi.org/10.1016/j.optlastec.2018.06.062
Ca2LaNb0.987O6Mn0.013	https://doi.org/10.1016/j.optlastec.2018.06.062
Ca2LaNb0.984O6Mn0.016	https://doi.org/10.1016/j.optlastec.2018.06.062
BaTi0.99OF4Mn0.01	https://doi.org/10.1016/j.optmat.2019.01.075
Sr2LaTa0.9995O6Mn0.0005	https://doi.org/10.1016/j.ijleo.2021.167348
Sr2LaTa0.999O6Mn0.001	https://doi.org/10.1016/j.ijleo.2021.167348
Sr2LaTa0.997O6Mn0.003	https://doi.org/10.1016/j.ijleo.2021.167348
Sr2LaTa0.995O6Mn0.005	https://doi.org/10.1016/j.ijleo.2021.167348
Sr2LaTa0.993O6Mn0.007	https://doi.org/10.1016/j.ijleo.2021.167348
Li3Mg2Nb0.999O6Mn0.001	https://doi.org/10.1111/jace.16527
Li3Mg2Nb0.998O6Mn0.002	https://doi.org/10.1111/jace.16527
Li3Mg2Nb0.997O6Mn0.003	https://doi.org/10.1111/jace.16527
Li3Mg2Nb0.996O6Mn0.004	https://doi.org/10.1111/jace.16527
Li3Mg2Nb0.995O6Mn0.005	https://doi.org/10.1111/jace.16527
Li3Mg2Nb0.994O6Mn0.006	https://doi.org/10.1111/jace.16527
Ba2Zr0.995F8Mn0.005	https://doi.org/10.1016/j.materresbull.2018.07.031
Ba2Zr0.9833F8Mn0.0167	https://doi.org/10.1016/j.materresbull.2018.07.031
Ba2Zr0.968F8Mn0.032	https://doi.org/10.1016/j.materresbull.2018.07.031
Ba2Zr0.96F8Mn0.04	https://doi.org/10.1016/j.materresbull.2018.07.031
Ba2Zr0.94F8Mn0.06	https://doi.org/10.1016/j.materresbull.2018.07.031
Ba2Zr0.92F8Mn0.08	https://doi.org/10.1016/j.materresbull.2018.07.031
Ba2Zr0.9F8Mn0.1	https://doi.org/10.1016/j.materresbull.2018.07.031
K2Mg0.995SiO4Mn0.005	https://doi.org/10.1088/1755-1315/714/3/032060
K2Mg0.99SiO4Mn0.01	https://doi.org/10.1088/1755-1315/714/3/032060
K2Mg0.98SiO4Mn0.02	https://doi.org/10.1088/1755-1315/714/3/032060

K2Mg0.96SiO4Mn0.04	https://doi.org/10.1088/1755-1315/714/3/032060
SrLaLiTe0.996O6Mn0.004	https://doi.org/10.1016/j.jlumin.2021.118286
SrLaLiTe0.992O6Mn0.008	https://doi.org/10.1016/j.jlumin.2021.118286
SrLaLiTe0.988O6Mn0.012	https://doi.org/10.1016/j.jlumin.2021.118286
SrLaLiTe0.984O6Mn0.016	https://doi.org/10.1016/j.jlumin.2021.118286
SrLaLiTe0.98O6Mn0.02	https://doi.org/10.1016/j.jlumin.2021.118286
SrLaLiTe0.976O6Mn0.024	https://doi.org/10.1016/j.jlumin.2021.118286
GdAl0.999O3Mn0.001	https://doi.org/10.1016/j.physb.2019.411953
GdAl0.995O3Mn0.005	https://doi.org/10.1016/j.physb.2019.411953
GdAl0.99O3Mn0.01	https://doi.org/10.1016/j.physb.2019.411953
GdAl0.985O3Mn0.015	https://doi.org/10.1016/j.physb.2019.411953
GdAl0.98O3Mn0.02	https://doi.org/10.1016/j.physb.2019.411953
Li2MgSn1.997O6Mn0.003	https://doi.org/10.1016/j.molstruc.2024.139004
Li2MgSn1.999O6Mn0.001	https://doi.org/10.1016/j.molstruc.2024.139004
Li2MgSn1.995O6Mn0.005	https://doi.org/10.1016/j.molstruc.2024.139004
Li2MgSn1.993O6Mn0.007	https://doi.org/10.1016/j.molstruc.2024.139004
Li2MgSn1.99O6Mn0.01	https://doi.org/10.1016/j.molstruc.2024.139004
Li2MgSn1.985O6Mn0.015	https://doi.org/10.1016/j.molstruc.2024.139004
Ca2LaTa0.996O6Mn0.004	https://doi.org/10.1021/acs.inorgchem.3c03939?urlappend=%3Fref%3DPDF&jav=VoR&rel=cite-as
Ca2LaTa0.998O6Mn0.002	https://doi.org/10.1021/acs.inorgchem.3c03939?urlappend=%3Fref%3DPDF&jav=VoR&rel=cite-as
Ca2LaTa0.994O6Mn0.006	https://doi.org/10.1021/acs.inorgchem.3c03939?urlappend=%3Fref%3DPDF&jav=VoR&rel=cite-as
Mg2Ti0.996O4Mn0.004	https://doi.org/10.1016/j.mtchem.2024.102308
Mg2Ti0.992O4Mn0.008	https://doi.org/10.1016/j.mtchem.2024.102308
Ba6Y2Ti3.985O17Mn0.015	https://doi.org/10.1016/j.optmat.2025.116793
Ba6Y2Ti3.99O17Mn0.01	https://doi.org/10.1016/j.optmat.2025.116793
Ba6Y2Ti3.98O17Mn0.02	https://doi.org/10.1016/j.optmat.2025.116793
Ba6Y2Ti3.995O17Mn0.005	https://doi.org/10.1016/j.optmat.2025.116793
Ca3La2W1.9975O12Mn0.0025	https://doi.org/10.1016/j.ceramint.2024.02.183
Ca3La2W1.99O12Mn0.01	https://doi.org/10.1016/j.ceramint.2024.02.183
Ca3La2W1.98O12Mn0.02	https://doi.org/10.1016/j.ceramint.2024.02.183
Li2Mg4Ti0.9992O7Mn0.0008	https://doi.org/10.1016/j.optmat.2024.116030

Li2Mg4Ti0.999O7Mn0.001	https://doi.org/10.1016/j.optmat.2024.116030
Li2Mg4Ti0.995O7Mn0.005	https://doi.org/10.1016/j.optmat.2024.116030
NaYBa4W1.999O12Mn0.001	https://doi.org/10.1016/j.optmat.2024.116030
NaYBa4W1.9975O12Mn0.0025	https://doi.org/10.1016/j.optmat.2024.116030
NaYBa4W1.995O12Mn0.005	https://doi.org/10.1016/j.optmat.2024.116030
NaLiSi0.91F6Mn0.09	https://doi.org/10.1016/j.optmat.2024.116519
NaLiTi0.94F6Mn0.06	https://doi.org/10.1016/j.optmat.2024.116519
Rb2Ge0.875F6Mn0.125	https://doi.org/10.1021/acs.jpcc.4c04465?urlappend=%3Fref%3DPDF&jav=VoR&rel=cite-as
CaLaLiTe0.994O6Mn0.006	https://doi.org/10.1016/j.jlumin.2023.120394
Sr3LiSb0.998O6Mn0.002	https://doi.org/0.1007/s10854-024-13251-7
Sr3LiSb0.997O6Mn0.003	https://doi.org/0.1007/s10854-024-13251-7
Sr3LiSb0.996O6Mn0.004	https://doi.org/0.1007/s10854-024-13251-7
Cs2Si0.9F6Mn0.1	https://doi.org/rg/10.2139/ssrn.4809204
Cs2Si0.95F6Mn0.05	https://doi.org/rg/10.2139/ssrn.4809204
Cs2Si0.98F6Mn0.02	https://doi.org/rg/10.2139/ssrn.4809204
SrLaNaTe0.98O6Mn0.02	https://doi.org/10.1016/j.ceramint.2023.11.161
SrLaNaTe0.99O6Mn0.01	https://doi.org/10.1016/j.ceramint.2023.11.161
SrLaNaTe0.96O6Mn0.04	https://doi.org/10.1016/j.ceramint.2023.11.161
SrLaNaTe0.992O6Mn0.008	https://doi.org/10.1016/j.ceramint.2023.11.161
NaMgLaTe0.98O6Mn0.02	https://doi.org/10.1016/j.dyepig.2018.09.084
NaMgLaTe0.99O6Mn0.01	https://doi.org/10.1016/j.dyepig.2018.09.084
NaMgLaTe0.994O6Mn0.006	https://doi.org/10.1016/j.dyepig.2018.09.084
NaMgLaTe0.96O6Mn0.04	https://doi.org/10.1016/j.dyepig.2018.09.084
Na3Li3In1.97F12Mn0.03	https://doi.org/10.1016/j.ceramint.2022.12.059
Na3Li3In1.99F12Mn0.01	https://doi.org/10.1016/j.ceramint.2022.12.059
Na3Li3In1.93F12Mn0.07	https://doi.org/10.1016/j.ceramint.2022.12.059
Sr2MgGe1.999O7Mn0.001	https://doi.org/10.1016/j.jallcom.2018.05.264
CaLaLiTe0.99O6Mn0.01	https://doi.org/10.1016/j.jallcom.2023.169436
Ba2LuNb0.994O6Mn0.006	https://doi.org/10.1016/j.jallcom.2023.169436
Ba2LuNb0.992O6Mn0.008	https://doi.org/10.1016/j.jallcom.2023.169436
Ba2LuNb0.996O6Mn0.004	https://doi.org/10.1016/j.jallcom.2023.169436

Ba2LuNb0.98O6Mn0.02	https://doi.org/10.1016/j.jallcom.2023.169436
Ba2GdSb0.999O6Mn0.001	https://doi.org/10.1039/C8DT01575A
Ba2GdSb0.995O6Mn0.005	https://doi.org/10.1039/C8DT01575A
Ba2GdSb0.993O6Mn0.007	https://doi.org/10.1039/C8DT01575A
Ba2GdSb0.99O6Mn0.01	https://doi.org/10.1039/C8DT01575A
Ca3Al3.996ZnO10Mn0.004	https://doi.org/10.1016/j.jallcom.2019.01.177
SrAl3.992O7Mn0.008	https://doi.org/10.1016/j.jallcom.2019.01.177
SrAl3.998O7Mn0.002	https://doi.org/10.1016/j.jallcom.2019.01.177
SrAl3.996O7Mn0.004	https://doi.org/10.1016/j.jallcom.2019.01.177
Ba2LaTa0.9998O6Mn0.002	https://doi.org/10.1016/j.ceramint.2022.08.170
NaSr10Y5W3.992O30Mn0.008	https://doi.org/10.1016/j.materresbull.2024.112957
NaSr10Y5W3.999O30Mn0.001	https://doi.org/10.1016/j.materresbull.2024.112957
NaSr10Y5W3.996O30Mn0.004	https://doi.org/10.1016/j.materresbull.2024.112957
NaSr10Y5W3.988O30Mn0.012	https://doi.org/10.1016/j.materresbull.2024.112957
Li2Ge0.998TeO60Mn0.002	https://doi.org/10.1016/j.apmt.2025.102598
Li2Ge0.996TeO60Mn0.004	https://doi.org/10.1016/j.apmt.2025.102598
Li2Ge0.994TeO60Mn0.006	https://doi.org/10.1016/j.apmt.2025.102598
Sr9Y2W3.995O24Mn0.005	https://doi.org/10.1016/j.ceramint.2024.06.112
Sr9Y2W3.9925O24Mn0.0075	https://doi.org/10.1016/j.ceramint.2024.06.112
Sr9Y2W3.99O24Mn0.01	https://doi.org/10.1016/j.ceramint.2024.06.112
LiAl3.998O6FMn0.002	https://doi.org/10.1016/j.optmat.2024.115148
LiAl3.996O6FMn0.004	https://doi.org/10.1016/j.optmat.2024.115148
LiAl3.994O6FMn0.006	https://doi.org/10.1016/j.optmat.2024.115148
La2LiTa0.997O6Mn0.003	https://doi.org/10.1016/j.optmat.2024.115148
La2LiTa0.999O6Mn0.001	https://doi.org/10.1016/j.optmat.2024.115148
La2LiTa0.995O6Mn0.005	https://doi.org/10.1016/j.optmat.2024.115148
La2LiTa0.993O6Mn0.007	https://doi.org/10.1016/j.optmat.2024.115148
La2LiTa0.991O6Mn0.009	https://doi.org/10.1016/j.optmat.2024.115148
Ca14Al9.999Zn6O35Mn0.001	https://doi.org/10.1016/j.jlumin.2025.121242
Ca14Al9.998Zn6O35Mn0.002	https://doi.org/10.1016/j.jlumin.2025.121242
K5Sc2.99F14Mn0.01	https://doi.org/10.1016/j.saa.2024.125382

Ca2LuNb0.996O6Mn0.004	https://doi.org/10.1016/j.ceramint.2024.03.364
Ca2LuNb0.998O6Mn0.002	https://doi.org/10.1016/j.ceramint.2024.03.364
Ca2LuNb0.994O6Mn0.006	https://doi.org/10.1016/j.ceramint.2024.03.364
Ca2LuNb0.992O6Mn0.008	https://doi.org/10.1016/j.ceramint.2024.03.364
Ba2TiGe1.997O8Mn0.003	https://doi.org/10.1016/j.dyepig.2017.06.061
Ba2TiGe1.994O8Mn0.006	https://doi.org/10.1016/j.dyepig.2017.06.061
Ba2TiGe1.991O8Mn0.009	https://doi.org/10.1016/j.dyepig.2017.06.061
Ba2TiGe1.988O8Mn0.012	https://doi.org/10.1016/j.dyepig.2017.06.061
Ba2TiGe1.985O8Mn0.015	https://doi.org/10.1016/j.dyepig.2017.06.061
Mg7Ga1.990GeO12Mn0.005	https://doi.org/10.1016/j.jallcom.2015.06.166
CaLaMgSb0.999O6Mn0.001	https://doi.org/10.1039/C8RA06708B
CaLaMgSb0.997O6Mn0.003	https://doi.org/10.1039/C8RA06708B
CaLaMgSb0.995O6Mn0.005	https://doi.org/10.1039/C8RA06708B
CaLaMgSb0.993O6Mn0.007	https://doi.org/10.1039/C8RA06708B
CaLaMgSb0.99O6Mn0.01	https://doi.org/10.1039/C8RA06708B
CaLaMgSb0.988O6Mn0.012	https://doi.org/10.1039/C8RA06708B
CaLaMgSb0.985O6Mn0.015	https://doi.org/10.1039/C8RA06708B
KMgLaTeO6Mn0.006	https://doi.org/10.1039/C7DT04811D
KMgLaTeO6Mn0.01	https://doi.org/10.1039/C7DT04811D
KMgLaTeO6Mn0.02	https://doi.org/10.1039/C7DT04811D
KMgLaTeO6Mn0.04	https://doi.org/10.1039/C7DT04811D
Gd2ZnTi0.9995O6Mn0.0005	https://doi.org/10.1039/C6TC00313C
Gd2ZnTi0.999O6Mn0.001	https://doi.org/10.1039/C6TC00313C
Gd2ZnTi0.998O6Mn0.002	https://doi.org/10.1039/C6TC00313C
Gd2ZnTi0.996O6Mn0.004	https://doi.org/10.1039/C6TC00313C
Gd2ZnTi0.992O6Mn0.008	https://doi.org/10.1039/C6TC00313C
Li2MgTi0.9999O4Mn0.0001	https://doi.org/10.1016/j.cej.2015.12.027
Li2MgTi0.9996O4Mn0.0004	https://doi.org/10.1016/j.cej.2015.12.027
Li2MgTi0.9993O4Mn0.0007	https://doi.org/10.1016/j.cej.2015.12.027
Li2MgTi0.999O4Mn0.001	https://doi.org/10.1016/j.cej.2015.12.027
Li2MgTi0.997O4Mn0.003	https://doi.org/10.1016/j.cej.2015.12.027

Li2MgTi0.995O4Mn0.005	https://doi.org/10.1016/j.cej.2015.12.027
Li2MgTi0.99O4Mn0.01	https://doi.org/10.1016/j.cej.2015.12.027
Ba2GdNb0.999O6Mn0.001	https://doi.org/10.1016/j.ceramint.2017.02.044
Ba2GdNb0.995O6Mn0.005	https://doi.org/10.1016/j.ceramint.2017.02.044
Ba2GdNb0.99O6Mn0.01	https://doi.org/10.1016/j.ceramint.2017.02.044
Ba2GdNb0.985O6Mn0.015	https://doi.org/10.1016/j.ceramint.2017.02.044
Ba2GdNb0.98O6Mn0.02	https://doi.org/10.1016/j.ceramint.2017.02.044
Ba2GdNb0.975O6Mn0.025	https://doi.org/10.1016/j.ceramint.2017.02.044
CaMg2Al15.984O27Mn0.016	https://doi.org/10.1021/am507316b
CaMg2Al15.968O27Mn0.032	https://doi.org/10.1021/am507316b
CaMg2Al15.92O27Mn0.08	https://doi.org/10.1021/am507316b
CaMg2Al15.84O27Mn0.16	https://doi.org/10.1021/am507316b
CaMg2Al15.76O27Mn0.24	https://doi.org/10.1021/am507316b
CaMg2Al15.52O27Mn0.48	https://doi.org/10.1021/am507316b
Ba2YNbO6Mn0.001	https://doi.org/10.1016/j.optmat.2017.05.028
Ba2YNbO6Mn0.005	https://doi.org/10.1016/j.optmat.2017.05.028
Ba2YNbO6Mn0.01	https://doi.org/10.1016/j.optmat.2017.05.028
Ba2YNbO6Mn0.015	https://doi.org/10.1016/j.optmat.2017.05.028
Ba2YNbO6Mn0.02	https://doi.org/10.1016/j.optmat.2017.05.028
Ba2YNbO6Mn0.025	https://doi.org/10.1016/j.optmat.2017.05.028
Mg2Al4Si5O18Mn0.005	https://doi.org/10.1016/j.dyepig.2017.08.050
Mg2Al4Si5O18Mn0.01	https://doi.org/10.1016/j.dyepig.2017.08.050
Mg2Al4Si5O18Mn0.015	https://doi.org/10.1016/j.dyepig.2017.08.050
Mg2Al4Si5O18Mn0.02	https://doi.org/10.1016/j.dyepig.2017.08.050
Mg2Al4Si5O18Mn0.025	https://doi.org/10.1016/j.dyepig.2017.08.050
Mg2Al4Si5O18Mn0.03	https://doi.org/10.1016/j.dyepig.2017.08.050
SrGe3.999O9Mn0.001	https://doi.org/10.1016/j.powtec.2016.01.017
SrGe3.996O9Mn0.004	https://doi.org/10.1016/j.powtec.2016.01.017
SrGe3.993O9Mn0.007	https://doi.org/10.1016/j.powtec.2016.01.017
SrGe3.99O9Mn0.01	https://doi.org/10.1016/j.powtec.2016.01.017
SrGe3.985O9Mn0.015	https://doi.org/10.1016/j.powtec.2016.01.017

SrGe3.98O9Mn0.02	https://doi.org/10.1016/j.powtec.2016.01.017
SrGe3.97O9Mn0.03	https://doi.org/10.1016/j.powtec.2016.01.017
Ca14Zn6Ga9.95O35Mn0.05	https://doi.org/10.1016/j.jallcom.2016.10.162
Ca14Zn6Ga9.9O35Mn0.1	https://doi.org/10.1016/j.jallcom.2016.10.162
Ca14Zn6Ga9.85O35Mn0.15	https://doi.org/10.1016/j.jallcom.2016.10.162
Ca14Zn6Ga9.8O35Mn0.2	https://doi.org/10.1016/j.jallcom.2016.10.162
Ca14Zn6Ga9.75O35Mn0.25	https://doi.org/10.1016/j.jallcom.2016.10.162
Ca14Zn6Ga9.7O35Mn0.3	https://doi.org/10.1016/j.jallcom.2016.10.162
Sr2LaNbO6Mn0.001	https://doi.org/10.1016/j.materresbull.2016.12.045
Sr2LaNbO6Mn0.005	https://doi.org/10.1016/j.materresbull.2016.12.045
Sr2LaNbO6Mn0.01	https://doi.org/10.1016/j.materresbull.2016.12.045
Sr2LaNbO6Mn0.015	https://doi.org/10.1016/j.materresbull.2016.12.045
Sr2LaNbO6Mn0.02	https://doi.org/10.1016/j.materresbull.2016.12.045
Sr2LaNbO6Mn0.25	https://doi.org/10.1016/j.materresbull.2016.12.045
SrLaGaO4Mn0.01	https://doi.org/10.1111/jace.15981
SrLaGaO4Mn0.0015	https://doi.org/10.1111/jace.15981
SrLaGaO4Mn0.002	https://doi.org/10.1111/jace.15981
SrLaGaO4Mn0.003	https://doi.org/10.1111/jace.15981
SrLaGaO4Mn0.005	https://doi.org/10.1111/jace.15981
SrLaGaO4Mn0.007	https://doi.org/10.1111/jace.15981
Li5La3Ta2O12Mn0.002	https://doi.org/10.1111/jace.16447
Li5La3Ta2O12Mn0.004	https://doi.org/10.1111/jace.16447
Li5La3Ta2O12Mn0.006	https://doi.org/10.1111/jace.16447
Li5La3Ta2O12Mn0.008	https://doi.org/10.1111/jace.16447
Li5La3Ta2O12Mn0.01	https://doi.org/10.1111/jace.16447
Ba2GdSb0.997O6Li0.003Mn0.003	https://doi.org/10.1039/C8DT01575A
Ba2GdSb0.997O6Mg0.003Mn0.003	https://doi.org/10.1039/C8DT01575A
Ba2GdSb0.997O6Ca0.003Mn0.003	https://doi.org/10.1039/C8DT01575A
Ba2GdSb0.997O6Sr0.003Mn0.003	https://doi.org/10.1039/C8DT01575A
Ba2GdSb0.985O6Mn0.015	https://doi.org/10.1039/C8DT01575A
Ba2GdSb0.98O6Mn0.02	https://doi.org/10.1039/C8DT01575A

LaMg0.5Ti0.4975O3Mn0.005	https://doi.org/10.1021/acsami.6b15866
LaMg0.5Ti0.497O3Mn0.006	https://doi.org/10.1021/acsami.6b15866
LaMg0.5Ti0.496O3Mn0.008	https://doi.org/10.1021/acsami.6b15866
LaMg0.5Ti0.495O3Mn0.01	https://doi.org/10.1021/acsami.6b15866
LaMg0.5Ti0.49O3Mn0.02	https://doi.org/10.1021/acsami.6b15866
LaAl0.998Mg0.001O3Mn0.001	https://doi.org/10.1021/acs.inorgchem.9b00457
La0.99Lu0.01Al0.998Mg0.001O3Mn0.001	https://doi.org/10.1021/acs.inorgchem.9b00457
La0.98Lu0.02Al0.998Mg0.001O3Mn0.001	https://doi.org/10.1021/acs.inorgchem.9b00457
La0.97Lu0.03Al0.998Mg0.001O3Mn0.001	https://doi.org/10.1021/acs.inorgchem.9b00457
La0.96Lu0.04Al0.998Mg0.001O3Mn0.001	https://doi.org/10.1021/acs.inorgchem.9b00457
Sr2MgGe2O7Mn0.001	https://doi.org/10.1016/j.jallcom.2018.05.264
Sr2MgGe2O7Mn0.003	https://doi.org/10.1016/j.jallcom.2018.05.264
Sr2MgGe2O7Mn0.005	https://doi.org/10.1016/j.jallcom.2018.05.264
Sr2MgGe2O7Mn0.007	https://doi.org/10.1016/j.jallcom.2018.05.264
Sr2MgGe2O7Mn0.009	https://doi.org/10.1016/j.jallcom.2018.05.264
Sr2MgGe2O7Mn0.011	https://doi.org/10.1016/j.jallcom.2018.05.264
Sr2MgGe2O7Mn0.02	https://doi.org/10.1016/j.jallcom.2018.05.264
Lu3Al4.998O12Mg0.001Mn0.001	https://doi.org/10.1039/C7TC02514A
Lu3Al4.994O12Mg0.003Mn0.003	https://doi.org/10.1039/C7TC02514A
Lu3Al4.986O12Mg0.007Mn0.007	https://doi.org/10.1039/C7TC02514A
Lu3Al4.98O12Mg0.01Mn0.01	https://doi.org/10.1039/C7TC02514A
Lu3Al4.97O12Mg0.015Mn0.015	https://doi.org/10.1039/C7TC02514A
Lu3Al4.96O12Mg0.02Mn0.02	https://doi.org/10.1039/C7TC02514A
Ca14Zn6Ga9.8O35Mn0.2	https://doi.org/10.1039/C7RA02112G
CaMg2La2W2O12Mn0.008	https://doi.org/10.1016/j.mtchem.2021.100512
CaMg2La2W2O12Mn0.006	https://doi.org/10.1016/j.mtchem.2021.100512
CaMg2La2W2O12Mn0.004	https://doi.org/10.1016/j.mtchem.2021.100512
CaMg2La2W2O12Mn0.01	https://doi.org/10.1016/j.mtchem.2021.100512
Ba2GdTaO6Mn0.002	https://doi.org/10.1021/acsomega.9b01787
Ba2GdTaO6Mn0.004	https://doi.org/10.1021/acsomega.9b01787
Ba2GdTaO6Mn0.006	https://doi.org/10.1021/acsomega.9b01787

Ba2GdTaO6Mn0.008	https://doi.org/10.1021/acsomega.9b01787
Ba2GdTaO6Mn0.01	https://doi.org/10.1021/acsomega.9b01787
Ba2GdTaO6Mn0.012	https://doi.org/10.1021/acsomega.9b01787
Ca2LaTa0.999O6Mn0.001	https://doi.org/10.1016/j.jallcom.2018.12.027
Ca2LaTa0.998O6Mn0.002	https://doi.org/10.1016/j.jallcom.2018.12.027
Ca2LaTa0.996O6Mn0.004	https://doi.org/10.1016/j.jallcom.2018.12.027
Ca2LaTa0.994O6Mn0.006	https://doi.org/10.1016/j.jallcom.2018.12.027
Ca2LaTa0.992O6Mn0.008	https://doi.org/10.1016/j.jallcom.2018.12.027
Ca2LaTa0.99O6Mn0.01	https://doi.org/10.1016/j.jallcom.2018.12.027
Ca2GdTaO6Mn0.002	https://doi.org/10.1016/j.jlumin.2019.116525
Ca2GdTaO6Mn0.004	https://doi.org/10.1016/j.jlumin.2019.116525
Ca2GdTaO6Mg0.004Mn0.004	https://doi.org/10.1016/j.jlumin.2019.116525
Ca2GdTaO6Li0.004Mn0.004	https://doi.org/10.1016/j.jlumin.2019.116525
Ca2GdTaO6Na0.004Mn0.004	https://doi.org/10.1016/j.jlumin.2019.116525
Ca2GdTaO6K0.004Mn0.004	https://doi.org/10.1016/j.jlumin.2019.116525
Ca2GdTaO6Mn0.006	https://doi.org/10.1016/j.jlumin.2019.116525
Ca2GdTaO6Mn0.008	https://doi.org/10.1016/j.jlumin.2019.116525
Ca2GdTaO6Mn0.01	https://doi.org/10.1016/j.jlumin.2019.116525
Ca2GdTaO6Mn0.012	https://doi.org/10.1016/j.jlumin.2019.116525
SrLaScO4Mn0.008	https://doi.org/10.1016/j.dyepig.2018.01.052
Ba2LaSbO6Mn0.0003	https://doi.org/10.1016/j.jlumin.2018.09.059
Ba2LaSbO6Mn0.0007	https://doi.org/10.1016/j.jlumin.2018.09.059
Ba2LaSbO6Mn0.001	https://doi.org/10.1016/j.jlumin.2018.09.059
Ba2LaSbO6Mn0.0013	https://doi.org/10.1016/j.jlumin.2018.09.059
Ba2LaSbO6Mn0.0015	https://doi.org/10.1016/j.jlumin.2018.09.059
Ba2LaSbO6Mn0.002	https://doi.org/10.1016/j.jlumin.2018.09.059
CaGdAlO4Mn0.0005	https://doi.org/10.1016/j.jlumin.2018.06.072
CaGdAlO4Mn0.001	https://doi.org/10.1016/j.jlumin.2018.06.072
CaGdAlO4Mn0.002	https://doi.org/10.1016/j.jlumin.2018.06.072
CaGdAlO4Mn0.004	https://doi.org/10.1016/j.jlumin.2018.06.072
CaGdAlO4Mn0.006	https://doi.org/10.1016/j.jlumin.2018.06.072

CaGdAlO4Mn0.008	https://doi.org/10.1016/j.jlumin.2018.06.072
CaGdAlO4Mn0.01	https://doi.org/10.1016/j.jlumin.2018.06.072
Ca14Al9.995Zn6O35Mn0.005	https://doi.org/10.1039/C7RA01285C
Sr4Al14O25Mn0.001	https://doi.org/10.1021/acs.chemmater.5b00226
Sr4Al14O25Mn0.002	https://doi.org/10.1021/acs.chemmater.5b00226
CaAl4O70Mn0.001	https://doi.org/10.1364/OE.21.018943
Mg3Ga2GeO8Mn0.001	https://doi.org/10.1021/acs.inorgchem.5b02048
Mg3Ga2GeO8Mn0.005	https://doi.org/10.1021/acs.inorgchem.5b02048
Mg3Ga2GeO8Mn0.01	https://doi.org/10.1021/acs.inorgchem.5b02048
Mg3Ga2GeO8Mn0.03	https://doi.org/10.1021/acs.inorgchem.5b02048
Mg3Ga2GeO8Mn0.05	https://doi.org/10.1021/acs.inorgchem.5b02048
Mg3Ga2GeO8Mn0.07	https://doi.org/10.1021/acs.inorgchem.5b02048
Sr6.2La3P2.5Si3B1.5O26Mn0.08	https://doi.org/10.1039/C4TC00217B
Ca2YSb0.999O6Mn0.001	https://doi.org/10.1039/C8DT00992A
Ca2YSb0.997O6Mn0.003	https://doi.org/10.1039/C8DT00992A
Ca2YSb0.995O6Mn0.005	https://doi.org/10.1039/C8DT00992A
Ca2YSb0.99O6Mn0.01	https://doi.org/10.1039/C8DT00992A
Ca2YSb0.985O6Mn0.015	https://doi.org/10.1039/C8DT00992A
Ca2YSb0.98O6Mn0.02	https://doi.org/10.1039/C8DT00992A
Ca2YSb0.997O6Li0.003Mn0.003	https://doi.org/10.1039/C8DT00992A
Ca2YSb0.997O6Mg0.003Mn0.003	https://doi.org/10.1039/C8DT00992A
Ca2YSb0.997O6Na0.003Mn0.003	https://doi.org/10.1039/C8DT00992A
Ca2YSb0.997O6K0.003Mn0.003	https://doi.org/10.1039/C8DT00992A
Sr2ZnW0.998O6Mn0.002	https://doi.org/10.1016/j.ceramint.2016.07.173
Sr2ZnW0.996O6Mn0.004	https://doi.org/10.1016/j.ceramint.2016.07.173
Sr2ZnW0.994O6Mn0.006	https://doi.org/10.1016/j.ceramint.2016.07.173
Sr2ZnW0.992O6Mn0.008	https://doi.org/10.1016/j.ceramint.2016.07.173
Sr2ZnW0.999O6Mn0.01	https://doi.org/10.1016/j.ceramint.2016.07.173
Sr2LaNb0.995O6Mn0.005	https://doi.org/10.1016/j.materresbull.2016.12.045
Sr2LaNb0.99O6Mn0.01	https://doi.org/10.1016/j.materresbull.2016.12.045
Sr2LaNb0.985O6Mn0.015	https://doi.org/10.1016/j.materresbull.2016.12.045

Sr2LaNb0.98O6Mn0.02	https://doi.org/10.1016/j.materresbull.2016.12.045
Sr2LaNb0.85O6Mn0.025	https://doi.org/10.1016/j.materresbull.2016.12.045
K2BaGe7.998O18Mn0.002	https://doi.org/10.1016/j.dyepig.2017.03.007
K2BaGe7.995O18Mn0.005	https://doi.org/10.1016/j.dyepig.2017.03.007
K2BaGe7.99O18Mn0.01	https://doi.org/10.1016/j.dyepig.2017.03.007
K2BaGe7.96O18Mn0.04	https://doi.org/10.1016/j.dyepig.2017.03.007
K2BaGe7.92O18Mn0.08	https://doi.org/10.1016/j.dyepig.2017.03.007
Li2Mg3Sn0.998O6Mn0.002	https://doi.org/10.1016/j.materresbull.2016.11.031
Li2Mg3Sn0.996O6Mn0.004	https://doi.org/10.1016/j.materresbull.2016.11.031
Li2Mg3Sn0.994O6Mn0.006	https://doi.org/10.1016/j.materresbull.2016.11.031
Li2Mg3Sn0.992O6Mn0.008	https://doi.org/10.1016/j.materresbull.2016.11.031
Li2Mg3Sn0.99O6Mn0.01	https://doi.org/10.1016/j.materresbull.2016.11.031
BaMgAl9.99O17Mg0.005Mn0.005	https://doi.org/10.1021/acs.chemmater.6b01303
BaMgAl9.98O17Mg0.01Mn0.01	https://doi.org/10.1021/acs.chemmater.6b01303
BaMgAl9.97O17Mg0.015Mn0.015	https://doi.org/10.1021/acs.chemmater.6b01303
BaMgAl9.96O17Mg0.02Mn0.02	https://doi.org/10.1021/acs.chemmater.6b01303
BaMgAl9.94O17Mg0.03Mn0.03	https://doi.org/10.1021/acs.chemmater.6b01303
BaMgAl9.92O17Mg0.04Mn0.04	https://doi.org/10.1021/acs.chemmater.6b01303
BaMgAl9.9O17Mg0.05Mn0.05	https://doi.org/10.1021/acs.chemmater.6b01303
LiAlGe1.998O6Mn0.002	https://doi.org/10.1016/j.jallcom.2015.07.080
LiAlGe1.996O6Mn0.004	https://doi.org/10.1016/j.jallcom.2015.07.080
LiAlGe1.996O6Mn0.006	https://doi.org/10.1016/j.jallcom.2015.07.080
LiAlGe1.992O6Mn0.008	https://doi.org/10.1016/j.jallcom.2015.07.080
LiAlGe1.99O6Mn0.01	https://doi.org/10.1016/j.jallcom.2015.07.080
LiGaGe1.998O6Mn0.002	https://doi.org/10.1016/j.jallcom.2015.07.080
LiGaGe1.996O6Mn0.004	https://doi.org/10.1016/j.jallcom.2015.07.080
LiGaGe1.994O6Mn0.006	https://doi.org/10.1016/j.jallcom.2015.07.080
LiGaGe1.992O6Mn0.008	https://doi.org/10.1016/j.jallcom.2015.07.080
LiGaGe1.99O6Mn0.01	https://doi.org/10.1016/j.jallcom.2015.07.080
K2Ge3.999O9Mn0.001	https://doi.org/10.1039/C6CP00168H
K2Ge3.995O9Mn0.005	https://doi.org/10.1039/C6CP00168H

K2Ge3.99O9Mn0.01	https://doi.org/10.1039/C6CP00168H
K2G3.974O9Mn0.03	https://doi.org/10.1039/C6CP00168H
K2Ge3.95O9Mn0.05	https://doi.org/10.1039/C6CP00168H
LiNaGe3.99995O9Mn0.00005	https://doi.org/10.1111/jace.14168
LiNaGe3.9999O9Mn0.0001	https://doi.org/10.1111/jace.14168
LiNaGe3.9995O9Mn0.0005	https://doi.org/10.1111/jace.14168
LiNaGe3.999O9Mn0.001	https://doi.org/10.1111/jace.14168
LiNaGe3.95O9Mn0.005	https://doi.org/10.1111/jace.14168
LiNaGe3.98O9Mn0.02	https://doi.org/10.1111/jace.14168
SrGe3.99O9Mn0.01	https://doi.org/10.1039/C6TC01813K
SrGe3.98O9Mn0.02	https://doi.org/10.1039/C6TC01813K
SrGe3.96O9Mn0.04	https://doi.org/10.1039/C6TC01813K
SrGe3.92O9Mn0.08	https://doi.org/10.1039/C6TC01813K
SrGe3.88O9Mn0.12	https://doi.org/10.1039/C6TC01813K
SrGe3.84O9Mn0.16	https://doi.org/10.1039/C6TC01813K
BaGe3.99O9Mn0.01	https://doi.org/10.1039/C6TC01813K
BaGe3.98O9Mn0.02	https://doi.org/10.1039/C6TC01813K
BaGe3.96O9Mn0.04	https://doi.org/10.1039/C6TC01813K
BaGe3.94O9Mn0.06	https://doi.org/10.1039/C6TC01813K
BaGe3.92O9Mn0.08	https://doi.org/10.1039/C6TC01813K
BaGe3.88O9Mn0.12	https://doi.org/10.1039/C6TC01813K
Li2Mg0.996ZrO4Mn0.004	https://doi.org/10.1016/j.jlumin.2017.05.002
Li2MgZr0.996O4Mn0.004	https://doi.org/10.1016/j.jlumin.2017.05.002
Cs2Si0.9925F6Mn0.01075	https://doi.org/10.1021/acsphotonics.7b00852
Cs2Si0.9887F6Mn0.0113	https://doi.org/10.1021/acsphotonics.7b00852
Cs2Si0.9659F6Mn0.0341	https://doi.org/10.1021/acsphotonics.7b00852
Cs2Si0.939F6Mn0.061	https://doi.org/10.1021/acsphotonics.7b00852
Cs2Si0.9075F6Mn0.0925	https://doi.org/10.1021/acsphotonics.7b00852
Cs3Al0.9975F6Mn0.1025	https://doi.org/10.1021/acsphotonics.7b00852
Cs3AlF6	https://doi.org/10.1149/2.0271809jss
