# Supplementary material for: Composition-based machine learning for predicting and designing Mn4+-doped phosphors
Source: RSC Adv. 2026 Feb 27;16(13):11415–25. doi: 10.1039/d6ra00029k (PMC12947634; doi:10.1039/d6ra00029k)
Supplement: RA-016-D6RA00029K-s002 [file RA-016-D6RA00029K-s002.pdf]

Supplementary Information (SI) for RSC Advances.
This journal is © The Royal Society of Chemistry 2026

**Actual formula**	**Predicted formula**
O60.00Mg10.00K5.00Mn0.01Sr10.00La5.00W9.99	O60.00Mg10.00K5.00Mn0.18Sr10.00La5.00W9.83
O60.00Mn0.04Y10.00Ba20.00Ta9.96	O60.00Mn0.08Y10.00Ba20.00Ta9.92
O60.00Mg10.00K10.00Mn0.15La10.00W9.85	O60.00Mg10.00K10.00Mn0.04La10.00W9.96
O59.94Mn0.24Ba19.98La9.99Ta9.85	O60.00Mn0.08Ba20.00La10.00Ta9.93
Li25.00O50.00Mg16.67Mn0.03Nb8.31	Li25.00O50.00Mg16.67Mn0.02Nb8.31
O22.22F44.44Na22.22Mn0.22W10.89	O22.22F44.44Na22.22Mn0.17W10.94
F70.00K20.00Mn0.05Nb9.95	F70.00K20.00Mn0.11Nb9.89
O52.63Ca17.54Mn0.05Y8.77In8.77Sb8.72Ta3.51	O52.63Ca17.54Mn0.06Y8.77In8.77Sb8.71Ta3.51
Li15.00F60.00Na15.00Al9.99Mn0.01	Li15.20F60.79Na15.20Al8.64Mn0.18
O1.82F32.73K9.09Mn0.00Nb56.36	O0.29F41.18Na3.16K9.17Mn0.14Ge29.82Rb4.27Nb8.90In3.07
O59.99Ca20.00Mn0.02Gd10.00Ta10.00	O59.97Ca19.99Mn0.06Gd10.00Ta9.98
Li18.18O54.55Mn0.09Zn9.09Sn18.09	Li18.18O54.55Mn0.05Zn9.09Sn18.14
Li33.33O50.00Mn0.07Sn16.60	Li33.33O50.00Mn0.11Sn16.56
F60.00K30.00Sc9.90Mn0.10	O2.50F57.50K25.00Sc7.43Ti2.49Mn0.08Rb5.00
Li10.00O60.00Mg10.00Mn0.20La10.00W9.80	Li10.00O60.00Mg10.00Mn0.08La10.00W9.92
O60.00Ti9.80Mn0.20Zn10.00Gd20.00	O60.79Ca3.95Ti7.46Mn0.06Zn7.50La2.63Gd15.00W2.62
O25.00Mg12.50Ti62.48Mn0.02	O55.81Mg2.53Al21.09Ca0.07Ti7.52Mn0.03Ge0.09Y12.87
O61.54Al15.35Mn0.04Ge15.38Ba7.69	O61.54Al15.33Mn0.05Ge15.38Ba7.69

O57.14Al14.26Mn0.03Sr14.29La14.29	O57.14Al14.22Mn0.06Sr14.29La14.29
O60.00Mn0.30Sr20.00Nb9.70Lu10.00	O60.00Mn0.30Sr20.00Nb9.70Lu10.00
O60.00Mn0.02Sb9.98Ba20.00La10.00	O60.00Mn0.03Sb9.97Ba20.00La10.00
Li26.09O52.17Mn0.06Sr4.35Sb8.63La8.70	Li26.09O52.17Mn0.04Sr4.35Sb8.66La8.70
O62.07K6.90Mn0.01Ge27.58Ba3.45	O62.07K6.90Mn0.16Ge27.43Ba3.45
O60.00Mn0.15Nb9.85Ba20.00Gd10.00	O60.00Mn0.12Nb9.88Ba20.00Gd10.00
O60.00Ti9.99Mn0.01Zn10.00La20.00	O60.00Ti9.97Mn0.03Zn10.00La20.00
O60.00Mn0.01Sb9.99Ba20.00La10.00	O60.00Mn0.03Sb9.97Ba20.00La10.00
O58.62Mg0.03Al34.41Mn0.03Ba3.45	O57.71Mg0.07Al26.85P3.30Mn0.20Sn3.16Ba6.00
O60.00Al24.70Mn0.30Lu15.00	O60.00Al24.77Mn0.23Lu15.00
F60.00Na30.00Mn0.05Ga9.95	F60.00Na30.00Mn0.37Ga9.63
O60.00Mg10.00Ca10.00Mn0.09Sb9.91La10.00	O60.00Mg10.00Ca10.00Mn0.05Sb9.95La10.00
O58.12Al32.53Mn0.02Sr9.30Pb0.03	O58.10Al32.51Cl0.06Mn0.03Sr9.30Pb0.00
F66.67Si10.73Mn0.38Cs22.22	F66.66Si10.55Mn0.57Cs22.22
O62.07Mg6.90Al13.74Si17.24Mn0.05	O62.07Mg6.90Al13.77Si17.24Mn0.03
O60.00Mn0.20Sb9.80Ba20.00Gd10.00	Li0.00O60.00Mg0.00Ca0.00Mn0.05Sr8.24Nb4.10Sb5.85Ba11.76Gd10.00
F70.00K20.00Mn0.41Nb9.59	F70.00K20.00Mn0.11Nb9.89
O60.00Ca20.00Mn0.10Gd10.00Ta9.90	O59.97Ca19.99Mn0.06Gd10.00Ta9.98
O14.29F57.14Ti14.14Mn0.14Ba14.29	H33.23O16.62F35.68Na1.53Si0.89K0.35Ti2.58Mn0.25Zn2.77Ge2.02Rb0.31Cs3.78

O58.33Mg8.33Mn0.06Ge16.61Sr16.67	O57.97Mg11.81Mn0.10Ga3.80Ge14.10Sr12.22
F33.33Mn0.11In58.22Ba8.33	F60.63Si4.66K18.19Ti3.86Mn0.56In10.59Ba1.52
O59.88Mn0.20Sr19.96Nb9.98La9.98	O59.67Mn0.55Sr19.89Nb9.95La9.95
O1.82F32.73K9.09Mn0.02Nb56.34	O0.29F41.18Na3.16K9.17Mn0.14Ge29.82Rb4.27Nb8.90In3.07
O20.00F40.00K30.00Mn0.69Ta9.32	O16.00F45.33Si1.98K24.00Mn0.80Cs4.44Ta7.44
O57.14Mg28.57Ti14.24Mn0.04	O57.14Mg28.57Ti14.18Mn0.11
F33.33Mn0.07In58.26Ba8.33	F60.63Si4.66K18.19Ti3.86Mn0.56In10.59Ba1.52
O60.00Sc10.00Mn0.06Sr20.00Sb9.94	O60.00Sc10.00Mn0.05Sr20.00Sb9.95
O59.38Al37.47Ca3.12Mn0.03	O59.38Al37.42Ca3.12Mn0.03Ge0.05
O57.13Mg28.57Si10.00Mn0.01Ge4.29	O57.13Mg28.57Si6.78Mn0.01Ge7.50
F60.00K10.00Mn0.14Rb20.00In9.86	F60.00K10.00Mn0.39Rb20.00In9.61
O60.00Mn0.04Sb9.96Ba20.00La10.00	O60.00Mn0.03Sb9.97Ba20.00La10.00
O59.02Mg1.64Al36.03Mn0.03Sr3.28	O59.02Mg1.64Al36.05Mn0.02Sr3.28
O60.00Ca10.00Mn0.03Ba20.00W9.97	O60.00Ca10.00Mn0.06Ba20.00W9.94
H14.29O7.14F35.71K21.43Mn0.57Mo6.57	H14.29O7.14F35.71K21.43Mn0.11Mo7.04
F63.64K27.27Mn0.00Zr9.09	F62.43K21.52Sc3.25Mn0.14Rb6.67Zr5.99
O60.00Ca20.00Mn0.05Nb9.95In10.00	O60.00Ca14.29Mn0.04Sr5.71Nb9.96In10.00
F66.67K22.22Ti10.56Mn0.56	F66.67K22.22Ti10.28Mn0.83
F60.00K10.00Mn0.09Rb20.00In9.91	F60.00K10.00Mn0.39Rb20.00In9.61

O57.10Mn0.07Ga14.28Sr14.28La14.28	O57.10Mn0.07Ga14.28Sr14.28La14.28
F66.67Si10.89K22.22Mn0.22	F60.63Si4.66K18.19Ti3.86Mn0.56In10.59Ba1.52
O58.33Mg8.33Mn0.09Ge16.57Sr16.67	O57.97Mg11.81Mn0.10Ga3.80Ge14.10Sr12.22
O59.95Mg0.04Ca19.98Mn0.04Gd9.99Ta9.99	O57.95Na0.01Ca3.46Mn0.05Ga9.83Sr9.97Y0.09Nb0.60In0.29Sb0.88Ba2.60La10.76Gd1.50Lu0.40Ta1.63
O60.00Mg0.02Al19.96Mn0.02La19.20Lu0.80	O60.00Mg0.02Al19.96Mn0.02La19.70Lu0.30
O60.00Mn0.20Nb9.80Ba20.00Gd10.00	O60.00Mn0.12Nb9.88Ba20.00Gd10.00
O60.00Mn0.07Sb9.93Ba20.00La10.00	O60.00Mn0.06Sb9.95Ba20.00La10.00
O63.16Mg10.53Ca5.26Mn0.08La10.53W10.45	O63.15Mg10.53Ca5.26Mn0.05La10.53W10.49
Li6.67O60.00Na6.67Mn0.01Ge26.66	Li6.67O60.04Na6.67Mn0.03Ge26.59
Li15.00F60.00Na15.00Al9.95Mn0.05	Li15.20F60.79Na15.20Al8.64Mn0.18
O22.58Al67.73Mn0.01Sr3.23La6.45	O22.58Al67.73Mn0.02Sr3.23La6.45
O60.00Mn0.04Nb9.96Ba20.00Lu10.00	O60.00Mn0.07Nb9.93Ba20.00Lu10.00
O59.88Mn0.20Y9.98Nb9.98Ba19.96	O59.94Mn0.10Y9.99Nb9.98Ba19.98
Li14.29O57.14Ti14.29Mn0.03Ga14.26	Li14.29O57.14Ti14.29Mn0.09Ga14.20
O60.00Mn0.15Sr20.00Nb9.85La10.00	O60.25Mn0.17Sr20.08Nb9.45La10.04
O59.95Ca19.98Mn0.08Gd9.99Ta9.99	O59.97Ca19.99Mn0.06Gd10.00Ta9.98
F70.00K20.00Mn0.10Ta9.90	F70.00K20.00Mn0.29Ta9.71
O57.14P14.29Mn1.14Sn13.14Ba14.29	O57.71Mg0.07Al26.85P3.30Mn0.20Sn3.16Ba6.00
O59.89Ca19.96Mn0.20Nb9.96Gd9.98	O59.97Ca19.99Mn0.08Nb9.95Gd10.00

O66.67Ti11.06Mn0.06Sb11.11La11.11	O66.67Ti11.03Mn0.08Sb11.11La11.11
O60.00Mn0.07Sr10.00Ba20.00W9.93	O60.00Mn0.03Sr10.00Ba20.00W9.97
O60.00Ca20.00Mn0.05Y10.00Sb9.95	Li0.00O59.99Na0.00Mg0.00K0.00Ca20.00Mn0.08Y10.00Sb6.31Ta3.61
O66.67Ti11.10Mn0.01Sb11.11La11.11	O66.67Ti11.03Mn0.08Sb11.11La11.11
Li2.50O17.50Na2.50Ti77.48Mn0.01	Li2.50O17.50Na2.50Ti77.49Mn0.01
O22.22F44.44Na22.22Mn0.06W11.06	O22.22F44.44Na22.22Mn0.17W10.94
F63.16Na10.53Ti5.26Mn1.68Ge8.84Cs10.53	H24.77Li1.15N6.19F49.24Na1.81Si1.56K5.75Ti4.16Mn0.38Ge2.10Sn1.06Cs1.81
O60.00Ca20.00Mn0.12Gd10.00Ta9.88	O59.97Ca19.99Mn0.06Gd10.00Ta9.98
O60.00Ca20.00Mn0.01Y10.00Sb9.99	Li0.00O59.99Na0.00Mg0.00K0.00Ca20.00Mn0.08Y10.00Sb6.31Ta3.61
O60.00Mn0.05Ba20.00La10.00Ta9.95	O60.00Mn0.05Ba20.00La10.00Ta9.95
O57.14Al28.34Ca14.29Mn0.23	O51.69Al25.85Ca12.74Mn9.72
O58.70Mg4.35Al34.75Ca2.17Mn0.03	O58.71Mg4.35Al34.74Ca2.17Mn0.03
Li19.05O57.14Ti23.71Mn0.10	Li19.05O57.14Ti23.63Mn0.17
F66.67K22.22Ti10.00Mn1.11	F66.67K22.22Ti10.28Mn0.83
O57.12Mg21.42Mn0.04Ga14.28Ge7.14	O57.97Mg11.81Mn0.10Ga3.80Ge14.10Sr12.22
O58.10Na0.12Al32.52Mn0.02Sr9.24	O58.10Na0.10Al32.52Mn0.02Sr9.25
O59.94Mg9.99Ti9.89Mn0.20La19.98	O59.98Mg9.96Ti9.92Mn0.15La19.99
O59.38Mg2.50Al37.48Ca0.62Mn0.02	O59.53Mg1.15Al37.58Ca1.72Mn0.02
O54.55Na9.09Mn0.09Sr27.27Sb9.00	O54.55Na9.09Mn0.04Sr27.27Sb9.05

O6.06F21.21V63.63Mn0.01Cs9.09	H11.11N2.78O4.55F24.24Na1.39V47.72Mn0.07In1.32Cs6.82
O59.94Ca19.98Mn0.10Gd9.99Ta9.99	O59.97Ca19.99Mn0.06Gd10.00Ta9.98
Li11.11F66.67K11.11Mn1.11Sn10.00	H24.77Li1.15N6.19F49.24Na1.81Si1.56K5.75Ti4.16Mn0.38Ge2.10Sn1.06Cs1.81
O60.00Mg2.00Ti9.90Mn0.10Zn8.00Gd20.00	O60.00Mg2.00Ti9.86Mn0.14Zn8.00Gd20.00
O60.00Ca20.00Mn0.09Nb9.91In10.00	O60.00Ca14.29Mn0.04Sr5.71Nb9.96In10.00
F69.97K19.99Mn0.05Nb9.00Mo1.00	F65.30K18.66Mn0.05Nb14.06Mo1.93
O59.93Mn0.12Ba19.98Gd9.99Ta9.99	O59.99Mn0.06Ba20.00Gd10.00Ta9.96
F66.67Na22.22Ti10.11Mn1.00	F66.55Na22.18Ti10.40Mn0.87
Li25.00O50.00Mg16.67Mn0.04Sb8.29	Li25.01O50.01Mg16.67Mn0.19Sb8.12
F66.67Na11.11Mn0.33Ge10.78Cs11.11	H3.85O3.85F41.03Na5.56K11.54Mn0.43Ge5.13Nb3.84Cs5.56
O60.00Ca20.00Mn0.06Sb9.94Gd10.00	O60.00Ca20.00Mn0.07Sb9.93Gd10.00
O57.14Mg0.02Al35.68Mn0.02Ba3.57	O57.71Mg0.07Al26.85P3.30Mn0.20Sn3.16Ba6.00
Li33.33O50.00Al8.25Mn0.08Sb8.33	Li33.33O50.00Al8.28Mn0.05Sb8.33
O30.77Al7.69Ca5.13Mn0.02Zr53.83Gd2.56	O30.77Al7.69Ca5.13Mn0.01Zr53.84Gd2.56
Li0.01O14.29Mg14.26Ti71.42Mn0.01Bi0.01	Li0.02O14.29Mg14.24Ti71.43Mn0.01Bi0.02
O53.85Al15.38Ca21.54Mn0.01Zn9.23	Li1.60O57.17Na0.40Mg0.40Al3.58Ca16.71Mn0.11Zn4.25Ga5.02Y2.60Sb2.59Te1.97La2.00Gd1.60
O57.14Mg28.57Ti14.27Mn0.01	O57.14Mg28.57Ti14.18Mn0.11
Li22.73O54.55Mn0.01Nb9.08La13.64	Li22.73O54.55Mn0.01Nb9.08La13.64
O60.00Ca20.00Mn0.06Gd10.00Ta9.94	O59.97Ca19.99Mn0.06Gd10.00Ta9.98

Li11.11F66.67K11.11Mn0.67Sn10.44	H24.77Li1.15N6.19F49.24Na1.81Si1.56K5.75Ti4.16Mn0.38Ge2.10Sn1.06Cs1.81
O60.00Mg0.01Al24.97Mn0.01Lu15.00	O60.00Mg0.06Al24.87Mn0.06Lu15.00
O59.38Al37.45Ca3.12Mn0.05	O59.38Al37.47Ca3.12Mn0.03
O62.06Mg6.90Al13.79Si17.24Mn0.02	O62.03Mg6.89Al13.78Si17.23Mn0.06
O64.29Mn0.86Ge27.71Sr7.14	O64.29Mn0.44Ge28.13Sr7.14
O60.00Al19.98Mn0.02Gd20.00	O60.00Al19.85Mn0.15Gd20.00
Li10.00O60.00Mn0.08Sr10.00Te9.92La10.00	Li10.00O60.00Mn0.15Sr10.00Te9.85La10.00
O66.67Ti11.11Mn0.01Sb11.10La11.11	O66.67Ti11.11Mn0.13Sb10.98La11.11
F66.67Si10.22K22.22Mn0.89	F60.63Si4.66K18.19Ti3.86Mn0.56In10.59Ba1.52
O58.14Al32.56Mn0.00Sr9.30	O58.13Al32.55Mn0.02Sr9.30
O50.00Mg12.44Si12.50K25.00Mn0.06	O50.00Mg12.21Si12.50K25.00Mn0.29
O60.00Ca20.00Mn0.04Sb9.96Gd10.00	O60.00Ca20.00Mn0.07Sb9.93Gd10.00
O60.00Al25.00Mn0.01Y15.00	O55.81Mg2.53Al21.09Ca0.07Ti7.52Mn0.03Ge0.09Y12.87
O61.54Ti7.69Mn0.02Ge15.36Ba15.38	O61.54Ti7.69Mn0.07Ge15.32Ba15.38
Li10.00O59.99Al10.00Mn0.06Ge19.96	Li10.00O60.00Al5.00Mn0.06Ga5.00Ge19.94
O60.00Al19.72Mn0.28La20.00	O60.00Na0.02Al19.84Mn0.17La19.98
O62.07K6.90Mn0.02Ge27.57Ba3.45	O62.07K6.90Mn0.16Ge27.43Ba3.45
F70.00K20.00Mn0.25Nb9.75	F70.00K20.00Mn0.11Nb9.89
O61.54Ti7.69Mn0.12Ge15.27Ba15.38	O61.54Ti7.69Mn0.07Ge15.32Ba15.38

O58.28Mg8.33Mn0.09Ge16.65Sr16.65	O57.97Mg11.81Mn0.10Ga3.80Ge14.10Sr12.22
O58.62Mg0.14Al34.21Mn0.14Ba3.45	O57.71Mg0.07Al26.85P3.30Mn0.20Sn3.16Ba6.00
Li10.00O60.00Mn0.04Ga10.00Ge19.96	Li10.00O60.00Al5.00Mn0.06Ga5.00Ge19.94
O60.00Ca20.00Mn0.03Y10.00Sb9.97	Li0.00O59.99Na0.00Mg0.00K0.00Ca20.00Mn0.08Y10.00Sb6.31Ta3.61
F66.67Na22.22Mn0.11Ge11.00	F66.68Si10.04K18.89Mn0.07Ge0.99Cs3.34
Li18.18O54.55Mn0.02Zn9.09Sn18.16	Li18.18O54.55Mn0.05Zn9.09Sn18.14
O60.00K13.33Mn0.03Ge26.63	O60.00K13.33Mn0.15Ge19.90
Li14.99O59.96Mn0.07La14.99W9.99	Li14.99O59.96Mn0.06La14.99W9.99
O57.96Al32.44Cl0.30Mn0.02Sr9.27	O58.10Al32.51Cl0.06Mn0.03Sr9.30Pb0.00
O60.00Ca20.00Mn0.08Y10.00Ta9.92	O60.00Ca20.00Mn0.05Y10.00Ta9.95
O60.00Mn0.01Sb9.99Ba20.00La10.00	O60.00Mn0.06Sb9.94Ba20.00La10.00
Li25.00O50.00Mg16.67Mn0.04Nb8.29	Li25.00O50.00Mg16.67Mn0.02Nb8.31
O6.06F21.21V63.63Mn0.01Cs9.09	H11.11N2.78O4.55F24.24Na1.39V47.72Mn0.07In1.32Cs6.82
Li25.00O50.00Mg16.67Mn0.02Nb8.32	Li25.00O50.00Mg16.67Mn0.02Nb8.31
O52.63Ca17.54Mn0.13Y8.77In8.77Sb8.64Ta3.51	O52.63Ca17.54Mn0.06Y8.77In8.77Sb8.71Ta3.51
O58.08Mg12.88Mn0.06La19.36Ta9.62	O58.08Mg12.88Mn0.05La19.36Ta9.63
O60.00Mn0.05Ba20.00La10.00Ta9.95	O58.41Sc7.85Mn0.11Sr7.93Ba8.89La12.38Ta4.43
O57.14Sc14.21Mn0.07Sr14.29La14.29	O58.41Sc7.85Mn0.11Sr7.93Ba8.89La12.38Ta4.43
O64.29Mn0.21Ge28.36Sr7.14	O64.29Mn0.07Ge28.50Sr7.14

O60.00Sc10.00Mn0.01Sr20.00Ta9.99	O59.95Mg4.44K4.44Sc5.56Mn0.12Sr11.11Te4.44La4.44Ta5.52
O53.85Ca21.54Mn0.38Zn9.23Ga15.00	O53.85Ca21.54Mn0.25Zn9.23Ga15.14
F60.00K30.00Sc9.93Mn0.07	O2.50F57.50K25.00Sc7.43Ti2.49Mn0.08Rb5.00
O60.00Ca10.00Mn0.02Ba20.00W9.98	O60.00Ca10.00Mn0.06Ba20.00W9.94
Li10.00O60.00Mn0.20Sr10.00Te9.80La10.00	Li10.00O60.00Mn0.43Sr10.00Te9.57La10.00
H44.44N11.11F33.33Na5.56Al5.39Mn0.17	H44.44N11.11F33.33Na5.56Al5.35Mn0.20
O40.00Na40.00Mg9.95Mn0.05W10.00	O40.00Na40.00Mg9.66Mn0.34W10.00
O58.13Al32.54Mn0.02Sr9.30Pb0.02	O58.10Al32.51Cl0.06Mn0.03Sr9.30Pb0.00
H46.15O23.08F23.08Ti3.60Mn0.25Zn3.85	H46.15O23.08F23.08Ti3.58Mn0.27Zn3.85
Li18.18O54.55Mn0.07Zn9.09Sn18.11	Li18.18O54.55Mn0.05Zn9.09Sn18.14
Li2.50O17.50Na2.50Ti77.49Mn0.01	Li2.50O17.50Na2.50Ti77.49Mn0.01
F60.00Na30.00Mn1.00Ga9.00	F60.00Na30.00Mn0.21Ga9.79
Li3.12O93.75Mn0.01Ge1.55Te1.56	Li3.12O93.75Mn0.01Ge1.56Te1.56
Li15.00F60.00Na15.00Al9.90Mn0.10	Li15.20F60.79Na15.20Al8.64Mn0.18
F66.66K22.22Ti11.09Mn0.03	H24.77Li1.15N6.19F49.24Na1.81Si1.56K5.75Ti4.16Mn0.38Ge2.10Sn1.06Cs1.81
O64.29Mn0.07Ge28.50Ba7.14	O64.29Mn0.46Ge28.11Ba7.14
O60.00Mg9.93Ti10.00Mn0.07La20.00	O59.65Mg9.80Ti9.94Mn0.72La19.88
O57.14Al14.23Ca14.29Mn0.06Gd14.29	O57.12Al14.25Ca14.28Mn0.06Gd14.28
Li6.67O60.00Na6.67Mn0.01Ge26.65	Li3.50O57.06Mg15.11Al13.44K0.48Ti5.67Mn0.07Ge1.92Sr1.34Ba0.24Ta1.16

O54.55Mg9.09Ca9.08Mn0.01Sb9.09Ba9.09La9.09	O54.55Mg9.09Ca9.04Mn0.05Sb9.09Ba9.09La9.09
O60.00Al25.00Mn0.00Y15.00	O55.81Mg2.53Al21.09Ca0.07Ti7.52Mn0.03Ge0.09Y12.87
H46.15O23.08F23.08Ti3.71Mn0.13Zn3.85	H46.15O23.08F23.08Ti3.58Mn0.27Zn3.85
O63.64P18.18K9.09Mn0.45Ga8.64	O63.64P18.18K9.09Mn0.55Ga8.55
Li25.00O50.00Mg12.50Ti12.44Mn0.06	Li25.00O50.00Mg12.50Ti12.49Mn0.01
O60.00Ca20.00Mn0.05Nb9.95In10.00	O60.00Ca20.00Mn0.03Nb9.97In10.00
F66.67Na22.22Si10.94Mn0.17	F70.26Na13.89Si8.12K3.17Mn0.39Ge1.39Rb2.78
Li0.04O59.95Ca19.98Mn0.04Gd9.99Ta9.99	O58.92Na0.02Ca10.39Ti0.40Mn0.04Zn0.40Ga5.27Sr5.29Nb0.30Sb0.40Ba1.40La6.49Gd5.20Lu0.30Ta5.20
O59.98Ti10.00Mn0.04Zn10.00Gd19.99	O59.99Ti9.99Mn0.03Zn10.00Gd20.00
O58.70Mg4.35Al34.77Ca2.17Mn0.01	O58.71Mg4.35Al34.74Ca2.17Mn0.03
O62.07K6.90Mn0.14Ge27.45Ba3.45	O62.07K6.90Mn0.16Ge27.43Ba3.45
Li25.00O50.00Mg16.67Mn0.02Nb8.32	Li25.00O50.00Mg16.67Mn0.06Nb8.27
O60.05K10.01Mn0.12Sr10.01Y10.01Te9.81	O60.00K10.00Mn0.06Sr10.00Y10.00Te9.94
Li18.18O54.55Mg9.09Mn0.03Sn18.15	Li18.18O54.55Mg9.09Mn0.07Sn18.11
F60.00Al9.10K30.00Mn0.90	F63.12Al6.95K21.82Mn0.46Ga3.76Ba3.90
F60.00K10.00Sc9.66Mn0.34Rb20.00	F62.43K21.52Sc3.25Mn0.14Rb6.67Zr5.99
O60.00Mn0.02Zn10.00Sr20.00W9.98	O59.99Mn0.07Zn10.00Sr20.00W9.95
H14.29O7.14F35.71K21.43Mn0.29Mo6.86	H14.29O7.14F35.71K21.43Mn0.11Mo7.04
O66.67Mn0.11Ge11.00Sb11.11La11.11	O66.71Mn0.09Ge10.97Sb11.12La11.12

O57.14Mg28.57Ti14.27Mn0.01	Li1.64O52.09Mg0.85Al23.73Ca11.34Mn8.66Sr0.07Sn1.63
F60.00K10.00Mn0.15Rb20.00In9.85	F60.00K10.00Mn0.39Rb20.00In9.61
O63.64P18.18K9.09Mn0.64Ga8.45	O63.64P18.18K9.09Mn0.55Ga8.55
O50.00Mn0.05Sr12.50La12.50	O54.46Mn0.06Ga8.89Sr13.62La13.62
Li7.69F30.77Na7.69Mn0.23In53.62	Li15.74F38.51Na15.74Mn0.14In29.86
O60.00Mn0.06Sr10.00Ba20.00W9.94	O60.00Mn0.03Sr10.00Ba20.00W9.97
O60.00Mg0.01Al24.99Mn0.01Lu15.00	O60.00Mg0.06Al24.87Mn0.06Lu15.00
Li10.00F60.00K20.00Mn0.25Ga9.75	Li10.00F60.00K20.00Mn0.90Ga9.10
O60.00Ca20.00Mn0.10Y10.00Sb9.90	O60.00Ca20.00Mn0.05Y10.00Sb9.95
O60.00Ca20.00Mn0.02Nb9.98Lu10.00	O60.00Ca20.00Mn0.06Nb9.94Lu10.00
O57.14Mg28.57Ti14.28Mn0.01	O57.14Mg28.57Ti14.21Mn0.08
F70.00K20.00Mn0.10Nb9.90	F70.00K20.00Mn0.50Nb9.50
H25.00O25.00F16.50Mn0.17Nb16.67Ba16.67	H25.00O25.00F15.67Mn1.00Nb16.67Ba16.67
O66.67Ca11.11Mn0.09Zr11.02La11.11	O65.56Ca10.93Mn0.19Zr10.74La12.59
Li25.00O50.00Mg16.67Mn0.29Sb8.04	Li25.01O50.01Mg16.67Mn0.19Sb8.12
O63.13Mg10.52Ca5.26Mn0.04La10.52W10.52	O63.15Mg10.53Ca5.26Mn0.05La10.53W10.49
O60.00Mn0.09Y8.00Nb9.91Ba20.00Eu2.00	O60.00Mn0.04Y8.00Nb9.96Ba20.00Eu2.00
Li19.05O57.14Ti23.57Mn0.24	Li19.05O57.14Ti23.63Mn0.17
O60.00Ca20.00Mn0.02La10.00Ta9.98	O60.00Ca20.00Mn0.06La10.00Ta9.94

O57.14Al14.20Mn0.09Sr14.29La14.29	O57.14Al14.22Mn0.06Sr14.29La14.29
Li10.00F60.00Al9.70K20.00Mn0.30	Li10.00O37.50F22.50Al3.54K7.50Mn0.25Nb6.22La12.50
O63.14Mg10.52Ca5.26Mn0.02La10.52W10.52	O63.15Mg10.53Ca5.26Mn0.05La10.53W10.49
O60.00Ca10.00Mn0.07Ba20.00W9.93	O60.00Ca10.00Mn0.06Ba20.00W9.94
O62.07Mg6.90Al13.71Si17.24Mn0.09	O62.07Mg6.90Al13.77Si17.24Mn0.03
O59.38Al37.49Mn0.01Sr3.12	Li0.02O59.33Na0.02Mg0.02Al37.46K0.02Mn0.01Sr3.12
O57.14Al14.14Mn0.14Sr14.29La14.29	O57.14Al14.22Mn0.06Sr14.29La14.29
O60.00Mg10.00Mn0.10Ba10.00La10.00Ta9.90	O60.00Mg10.00Mn0.06Ba10.00La10.00Ta9.94
F70.00K20.00Mn0.30Ta9.70	F70.00K20.00Mn0.29Ta9.71
O20.00F40.00K30.00Mn0.74Ta9.25	O16.00F45.33Si1.98K24.00Mn0.80Cs4.44Ta7.44
O60.00Mn0.08Y10.00Ba20.00Ta9.92	O60.00Mn0.05Y10.00Ba20.00Ta9.95
O60.00Mn0.06Zn10.00Sb9.94Ba10.00La10.00	O60.00Mn0.07Zn10.00Sb9.93Ba10.00La10.00
O58.62Mg3.45Al34.45Mn0.03Sr3.45	O58.62Mg3.45Al34.45Mn0.03Sr3.45
O60.00Ca20.00Mn0.04Y10.00Ta9.96	Li0.00O59.99Na0.00Mg0.00K0.00Ca20.00Mn0.08Y10.00Sb6.31Ta3.61
O60.00Ti9.96Mn0.04Zn10.00Gd20.00	O59.99Ti9.99Mn0.03Zn10.00Gd20.00
O60.00Mg20.00Mn0.05In10.00Sb9.95	O60.00Mg20.00Mn0.05In10.00Sb9.95
O60.00Mg10.00Ca10.00Mn0.05Sb9.95La10.00	O60.00Mg10.00Ca10.00Mn0.08Sb9.92La10.00
O58.42Mg9.44Ti9.74Mn2.92La19.47	O59.65Mg9.80Ti9.94Mn0.72La19.88
O22.58Al67.73Mn0.01Sr3.23La6.45	O22.58Al67.73Mn0.01Sr3.23La6.45

O60.00Mg10.00Ca10.00Mn0.03Sb9.97La10.00	O60.00Mg10.00Ca10.00Mn0.08Sb9.92La10.00
O63.16Ca15.79Mn0.05La10.53W10.47	O60.79Ca3.95Ti7.46Mn0.06Zn7.50La2.63Gd15.00W2.62
F66.59K22.20Ti6.66Mn0.11Ge4.44	F65.76K21.92Ti5.44Mn0.11Ge6.77
O59.38Mg1.88Al37.48Ca1.25Mn0.02	O59.38Mg1.56Al37.48Ca1.56Mn0.02
O60.00Mg10.00Mn0.12Sb9.88Ba10.00La10.00	O60.00Mg10.00Mn0.06Sb9.94Ba10.00La10.00
O58.34Mg8.33Ge16.66Sr16.67	O57.97Mg11.81Mn0.10Ga3.80Ge14.10Sr12.22
O60.00Ca10.00Mn0.03Ba20.00W9.97	O60.00Ca10.00Mn0.07Ba20.00W9.93
O60.00Mn0.02Sr10.00Ba20.00W9.98	O60.00Mn0.03Sr10.00Ba20.00W9.97
O60.00Mg10.00Ca10.00Mn0.07Nb9.93Gd10.00	O60.00Mg10.00Ca10.00Mn0.04Nb9.96Gd10.00
F75.00Ti12.25Mn0.25Ba12.50	F75.00Ti10.94Mn1.56Ba12.50
Li14.29O57.14Ti20.71Mn0.71Zn7.14	Li14.29O57.14Ti20.32Mn1.11Zn7.14
O60.00Mn0.05Sr20.00La10.00Ta9.95	O60.00Mn0.03Sr20.00La10.00Ta9.97
O53.85Ca21.54Mn0.38Zn9.23Ga15.00	O53.85Ca21.54Mn0.22Zn9.23Ga15.17
Li10.00O60.00Ca10.00Mn0.02Te9.98La10.00	Li10.00O60.00Ca10.00Mn0.09Te9.91La10.00
O60.00Mg10.00Ca10.00Mn0.15Sb9.85La10.00	O60.00Mg10.00Ca10.00Mn0.08Sb9.92La10.00
O60.00Mn0.01Sr20.00Gd10.00Ta9.99	O60.00Mn0.07Sr20.00Gd10.00Ta9.93
O60.00Mn0.01Sr20.00In10.00Sb9.99	O60.00Mn0.15Sr20.00In10.00Sb9.85
O62.07Mg6.90Al13.69Si17.24Mn0.10	O62.07Mg6.90Al13.77Si17.24Mn0.03
O60.00Mn0.05Sr20.00In10.00Sb9.95	O60.00Mn0.15Sr20.00In10.00Sb9.85

O59.94Mn0.10Ba19.98Gd9.99Ta9.99	O59.99Mn0.06Ba20.00Gd10.00Ta9.96
F60.00Na10.00Al9.95Mn0.05Rb20.00	F60.00Na10.00Al9.58Mn0.42Rb20.00
O59.02Mg1.64Al36.05Mn0.01Sr3.28	O59.02Mg1.64Al36.05Mn0.02Sr3.28
O63.16Ca15.79Mn0.02La10.53W10.51	O63.16Ca15.79Mn0.06La10.53W10.47
H47.06N11.76F35.29Ti5.76Mn0.12	H24.77Li1.15N6.19F49.24Na1.81Si1.56K5.75Ti4.16Mn0.38Ge2.10Sn1.06Cs1.81
F70.00K20.00Mn0.53Nb9.47	F70.00K20.00Mn0.11Nb9.89
O59.38Al37.38Ca3.12Mn0.03Ge0.09	O59.38Al37.42Ca3.12Mn0.03Ge0.05
F60.00Na30.00Mn0.50Ga9.50	F60.00Na30.00Mn0.37Ga9.63
O60.14Mn0.03Nb9.77Ba20.05La10.02	O60.10Mn0.08Nb9.76Ba20.03La10.02
O58.08Mg12.88Mn0.10La19.36Ta9.58	O58.08Mg12.88Mn0.05La19.36Ta9.63
O60.00Al19.60Mn0.40Gd20.00	O60.00Al19.85Mn0.15Gd20.00
O30.77Al7.69Ca5.13Mn0.00Zr53.84Gd2.56	O30.77Al7.69Ca5.13Mn0.01Zr53.84Gd2.56
Li10.00O60.00Mg10.00Mn0.01La10.00W9.99	Li10.00O60.00Mg10.00Mn0.08La10.00W9.92
F60.00Na10.00Al9.90Mn0.10Rb20.00	F60.00Na10.00Al9.58Mn0.42Rb20.00
Li25.00O50.00Mg16.67Mn0.05Nb8.28	Li25.00O50.00Mg16.67Mn0.02Nb8.31
O62.03Mg6.89Al13.78Si17.23Mn0.07	O62.03Mg6.89Al13.78Si17.23Mn0.06
O22.58Al67.74Mn0.01Sr3.23La6.45	O22.58Al67.73Mn0.01Sr3.23La6.45
O60.00Mn0.10Sr20.00Nb9.90La10.00	O60.25Mn0.17Sr20.08Nb9.45La10.04
O60.00Mg10.00K10.00Mn0.03La10.00W9.97	O60.00Mg10.00K10.00Mn0.04La10.00W9.96

O58.70Mg4.35Al34.26Ca2.17Mn0.52	O58.65Mg4.34Al34.48Ca2.17Mn0.35
O60.00Ca20.00Mn0.04La10.00Ta9.96	O60.00Ca20.00Mn0.04La10.00Ta9.96
O60.00Mn0.03Sr20.00La10.00Ta9.97	O60.00Mn0.03Sr20.00La10.00Ta9.97
F71.43Mn0.63Ga13.66Ba14.29	F63.12Al6.95K21.82Mn0.46Ga3.76Ba3.90
O60.00K10.00Mn0.06Sr10.00Y10.00Te9.94	O60.00K10.00Mn0.06Sr10.00Y10.00Te9.94
O58.14Al32.43Mn0.13Sr9.30	O58.10Al32.51Cl0.06Mn0.03Sr9.30Pb0.00
O66.67Ti11.11Mn0.03Sb11.08La11.11	O66.67Ti11.11Mn0.13Sb10.98La11.11
O60.00Mn0.20Sb9.80Ba20.00La10.00	O60.00Mn0.06Sb9.95Ba20.00La10.00
O60.00Mg10.00K10.00Mn0.10La10.00W9.90	O60.00Mg10.00K10.00Mn0.04La10.00W9.96
O60.00Na10.00Mg10.00Mn0.12La10.00W9.88	O60.00Na10.00Mg10.00Mn0.07La10.00W9.93
H14.29O7.14F35.71K21.43Mn0.07W7.07	H9.53O4.77F50.62K14.30Ti4.46Mn0.22Ba4.46W2.10
O57.14P14.29Mn1.43Sn12.86Ba14.29	O57.71Mg0.07Al26.85P3.30Mn0.20Sn3.16Ba6.00
O60.00Mn0.80Y10.00Sb9.20Ba20.00	O60.00Mn0.57Y10.00Sb9.43Ba20.00
Li7.69F30.77Na7.69Mn0.18In53.67	Li15.74F38.51Na15.74Mn0.14In29.86
O57.14Mg28.57Ti14.28Mn0.00	O57.14Mg28.57Ti14.21Mn0.08
Li25.00O50.00Mg16.67Mn0.08Sb8.25	Li25.01O50.01Mg16.67Mn0.19Sb8.12
O58.62Mg3.45Al34.36Mn0.12Sr3.45	O58.62Mg3.45Al34.44Mn0.04Sr3.45
F60.00K30.00Sc9.48Mn0.52	O2.50F57.50K25.00Sc7.43Ti2.49Mn0.08Rb5.00
F66.67Mn0.84Rb22.22Zr10.27	F66.67Mn0.25Rb22.22Zr10.86

O62.02Mg6.89Al13.78Si17.23Mn0.09	O62.03Mg6.89Al13.78Si17.23Mn0.06
O57.13Al14.28Ca14.28Mn0.03Gd14.28	O57.12Al14.25Ca14.28Mn0.06Gd14.28
O59.38Mg1.25Al37.48Ca1.88Mn0.02	O59.38Mg1.56Al37.48Ca1.56Mn0.02
O60.06Al24.42Ca0.40Mn0.10Y15.02	O55.81Mg2.53Al21.09Ca0.07Ti7.52Mn0.03Ge0.09Y12.87
O22.22F44.44Na22.22Mn0.11W11.00	O22.22F44.44Na22.22Mn0.17W10.94
O60.00Ca20.00Mn0.04Gd10.00Ta9.96	O59.97Ca19.99Mn0.06Gd10.00Ta9.98
O60.00Ca20.00Mn0.08Nb9.92Gd10.00	O59.97Ca19.99Mn0.08Nb9.95Gd10.00
O59.38Al37.50Ca3.12Mn0.00	O59.38Al37.47Ca3.12Mn0.03
F66.67K22.22Ti9.37Mn1.74	F66.67K22.22Ti11.10Mn0.01
O59.95Mg9.99Ti9.91Mn0.16La19.98	O59.98Mg9.96Ti9.92Mn0.15La19.99
F28.00K10.00Mn0.04In61.96	Li1.11F31.87Na1.11K9.00Ti1.04Mn0.09In55.77
F32.43Mn0.14Ge56.62Rb2.70Cs8.11	F43.84Mn0.29Ge41.25Rb5.51Cs9.11
O60.00Mn0.08Zn10.00Sr10.00Nb9.92La10.00	O60.00Mn0.04Zn10.00Sr10.00Nb9.96La10.00
O53.85Ca21.54Mn0.14Zn9.23Ga15.25	O53.85Ca21.54Mn0.22Zn9.23Ga15.17
F66.67Si10.00Mn1.11Cs22.22	F66.67Si10.72Mn0.39Cs22.22
O60.00Ca10.00Mn0.12Ba20.00W9.88	O60.00Ca10.00Mn0.07Ba20.00W9.93
F66.67Mn1.52Rb22.22Zr9.59	F66.67Mn0.25Rb22.22Zr10.86
O60.00Mn0.08Y10.00Ba20.00Ta9.92	O60.00Mn0.08Y10.00Ba20.00Ta9.92
O25.00Mg12.50Ti62.50Mn0.00	O47.96Mg23.98Ti28.05Mn0.01

O22.58Al67.73Mn0.01Sr3.23La6.45	O22.58Al67.73Mn0.02Sr3.23La6.45
O57.14Al14.17Ca14.29Mn0.11Gd14.29	O57.12Al14.25Ca14.28Mn0.06Gd14.28
Li25.00O50.00Mg16.67Mn0.08Nb8.25	Li25.00O50.00Mg16.67Mn0.06Nb8.27
Li15.00F60.00Na15.00Mn0.15In9.85	Li15.74F38.51Na15.74Mn0.14In29.86
F63.64K27.27Mn0.06Zr9.03	F62.43K21.52Sc3.25Mn0.14Rb6.67Zr5.99
F60.00K30.00Sc9.65Mn0.35	O2.50F57.50K25.00Sc7.43Ti2.49Mn0.08Rb5.00
F66.67Si10.22K22.22Mn0.89	F70.26Na13.89Si8.12K3.17Mn0.39Ge1.39Rb2.78
O36.40Al18.11Mn36.40Sr9.10	O51.69Al25.85Ca12.74Mn9.72
Li14.29O57.14Ti14.29Mn0.17Ga14.11	Li14.29O57.14Ti14.29Mn0.09Ga14.20
H46.15O23.08F23.08Ti3.02Mn0.83Zn3.85	H46.15O23.08F23.08Ti3.58Mn0.27Zn3.85
F63.16Na10.53Ti5.26Mn1.26Ge9.26Cs10.53	H24.77Li1.15N6.19F49.24Na1.81Si1.56K5.75Ti4.16Mn0.38Ge2.10Sn1.06Cs1.81
O60.00Mn0.05Nb9.95Ba20.00Gd10.00	O60.00Mn0.12Nb9.88Ba20.00Gd10.00
Li25.00O50.00Mg12.50Ti12.38Mn0.12	Li25.00O50.00Mg12.50Ti12.49Mn0.01
O56.94Mg21.35Mn0.36Ga14.23Ge7.12	O57.97Mg11.81Mn0.10Ga3.80Ge14.10Sr12.22
O58.33Mg8.33Ge16.67Sr16.67	O57.97Mg11.81Mn0.10Ga3.80Ge14.10Sr12.22
Li14.99O59.97Mn0.05La14.99W10.00	Li14.99O59.96Mn0.06La14.99W9.99
O50.01Mn0.03Sr12.50La12.50	O54.46Mn0.06Ga8.89Sr13.62La13.62
O57.14Mg28.57Ti14.07Mn0.21	O57.14Mg28.57Ti14.21Mn0.08
O60.01Mg9.96Ti9.96Mn0.06La20.00	O59.98Mg9.96Ti9.92Mn0.15La19.99

O62.07Mg6.90Al13.72Si17.24Mn0.07	O62.07Mg6.90Al13.77Si17.24Mn0.03
O60.00Mg0.40Al24.59Mn0.01Y15.00	O55.81Mg2.53Al21.09Ca0.07Ti7.52Mn0.03Ge0.09Y12.87
F66.67Na22.22Si10.56Mn0.56	F66.67Na22.22Si10.56Mn0.56
O60.00Ca20.00Sc10.00Mn0.10Ta9.90	O60.00Ca20.00Sc10.00Mn0.04Ta9.96
O60.00Ca20.00Mn0.08Y10.00Sb9.92	O60.00Ca20.00Mn0.05Y10.00Sb9.95
O60.00Mn0.08Sb9.92Ba20.00La10.00	O60.00Mn0.06Sb9.94Ba20.00La10.00
O60.00Mg10.00Ca10.00Mn0.10Sr10.00W9.90	O60.00Mg10.00Ca10.00Mn0.13Sr10.00W9.87
F60.00K10.00Sc9.85Mn0.15Rb20.00	F62.43K21.52Sc3.25Mn0.14Rb6.67Zr5.99
Li22.73O54.55Mn0.03La13.64Ta9.06	Li22.72O54.54Mn0.03La13.63Ta9.08
O40.00Na40.00Mg9.30Mn0.70W10.00	O40.00Na40.00Mg9.66Mn0.34W10.00
O60.00Mn0.08Nb9.92Ba20.00Lu10.00	O60.00Mn0.07Nb9.93Ba20.00Lu10.00
O60.00Ca20.00Mn0.02Y10.00Sb9.98	O60.00Ca20.00Mn0.05Y10.00Sb9.95
Li10.00O60.00Mn0.05La20.00Ta9.95	Li10.00O60.00Mn0.05La20.00Ta9.95
O52.63Ca17.54Mn0.02Y8.77In8.77Sb8.75Ta3.51	O52.63Ca17.54Mn0.06Y8.77In8.77Sb8.71Ta3.51
Li22.72O54.53Mn0.03La13.63Ta9.09	Li22.72O54.54Mn0.03La13.63Ta9.08
O40.00Na40.00Mg9.60Mn0.40W10.00	O40.00Na40.00Mg9.66Mn0.34W10.00
H14.29O7.14F35.71K21.43Mn0.43Mo6.71	H14.29O7.14F35.71K21.43Mn0.11Mo7.04
O60.00Ca10.00Mn0.09Sr10.00Nb9.91In10.00	O60.00Ca14.29Mn0.04Sr5.71Nb9.96In10.00
O60.00Al19.70Mn0.30Gd20.00	O60.00Al19.85Mn0.15Gd20.00

O60.00Sc10.00Mn0.02Sr20.00Nb9.98	O60.00Sc10.00Mn0.12Sr20.00Nb9.88
O60.00Ca10.00Mn0.06Ba20.00W9.94	O60.00Ca10.00Mn0.07Ba20.00W9.93
Li10.00O60.00Mn0.05Ba10.00La10.00W9.95	Li10.00O60.00Mn0.15Ba10.00La10.00W9.85
O60.00Mn0.10Nb9.90Ba20.00La10.00	O60.10Mn0.08Nb9.76Ba20.03La10.02
Li10.00F60.00Al9.50K20.00Mn0.50	Li10.00O37.50F22.50Al3.54K7.50Mn0.25Nb6.22La12.50
O60.00Ca20.00Mn0.02Zr19.98	Li17.02O53.25Mg11.30Al7.61Ca0.20Mn0.06Ge0.53Sr0.34Nb5.64In0.10La0.10Lu3.75Ta0.10
