# Supplementary material for: Composition-based machine learning for predicting and designing Mn4+-doped phosphors
Source: RSC Adv. 2026 Feb 27;16(13):11415–25. doi: 10.1039/d6ra00029k (PMC12947634; doi:10.1039/d6ra00029k)
Supplement: RA-016-D6RA00029K-s003 [file RA-016-D6RA00029K-s003.pdf]

Supplementary Information (SI) for RSC Advances.
This journal is © The Royal Society of Chemistry 2026

Composition-Based Machine Learning for Predicting and Designing Mn^{4+}-doped Phosphors

Ngo T. Que,[1] Vu D. Huan,[2] Le T. Duy,[2] Vu N. Bao,[2] Vu L. Minh,[3] Mai X. Trang,[4] Anh D. Phan,[2,1,*] and Pham T. Huy[2]

[1]*Phenikaa Institute for Advanced Study, Phenikaa University, Hanoi 12116, Vietnam*
[2]*Faculty of Materials Science and Engineering, Phenikaa University, Hanoi 12116, Vietnam*
[3]*Applied Artificial Intelligence Institute (A2I2), Deakin University, Australia*
[4]*School of Computing, Phenikaa University, Hanoi 12116, Vietnam*

(Dated: February 5, 2026)

[*]Electronic address: anh.phanduc@phenikaa-uni.edu.vn

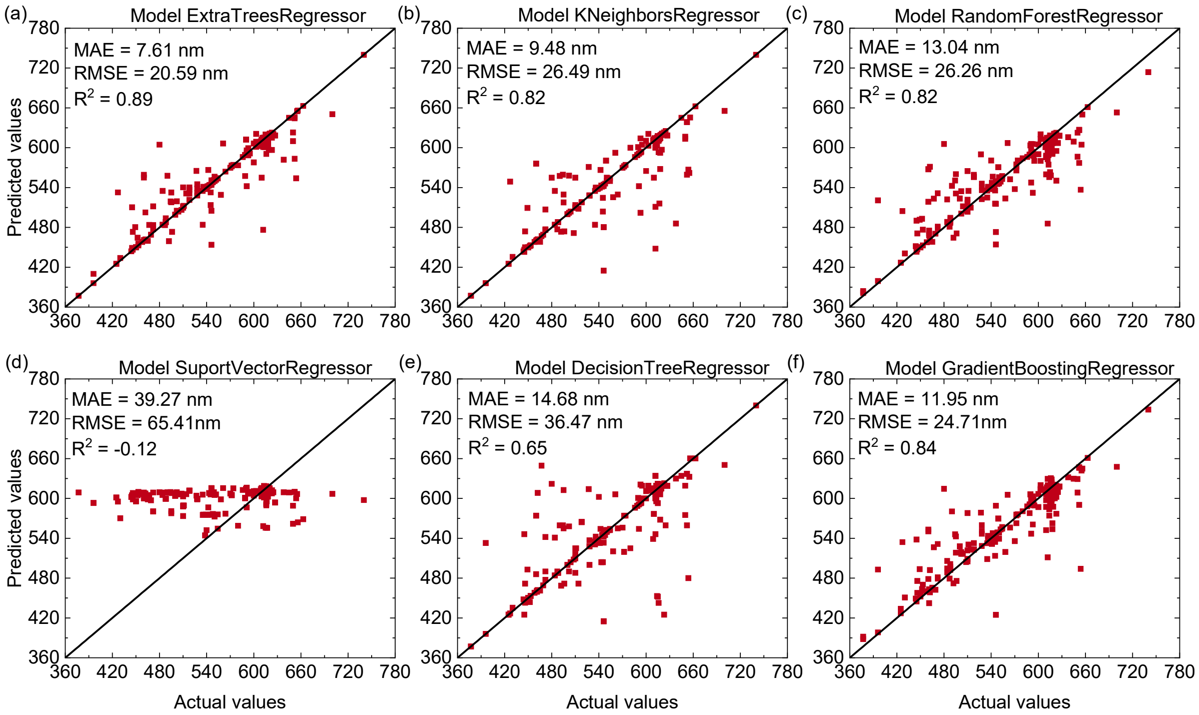

Figure S 1: (Color online) Predictive performance of six regression models on the testing dataset for emission wavelength prediction of phosphor Eu^{3+} including (a) Extra Trees Regressor, (b) K-Nearest Neighbors Regressor , (c)Random Forest Regressor , (d) Support Vector Regressor, (e) Decision Tree Regressor, (f) Gradient Boosting Regressor.

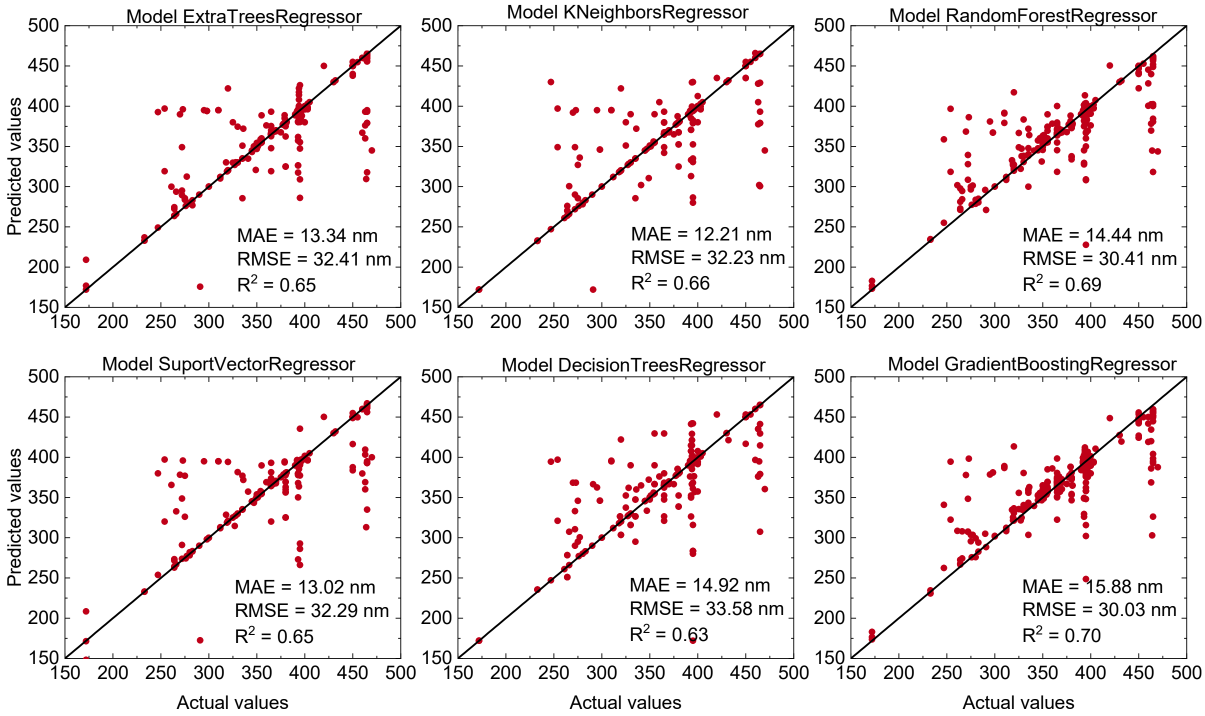

Figure S 2: (Color online) Predictive performance of six regression models on the testing dataset for excitation wavelength prediction of phosphor Eu^{3+} including (a) Extra Trees Regressor, (b) K-Nearest Neighbors Regressor , (c)Random Forest Regressor , (d) Support Vector Regressor, (e) Decision Tree Regressor, (f) Gradient Boosting Regressor.

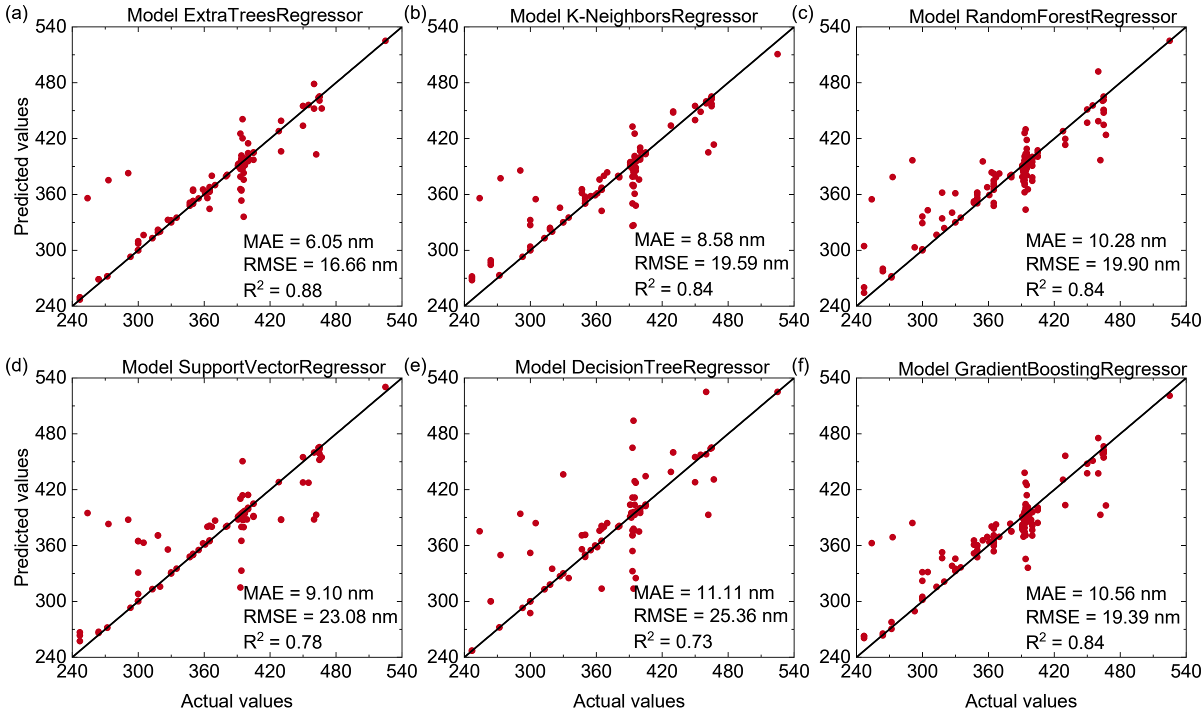

Figure S 3: (Color online) Predictive performance of six regression models on the testing dataset for excitation 1st peak wavelength prediction of phosphor Eu^{3+} including (a) Extra Trees Regressor, (b) K-Nearest Neighbors Regressor , (c)Random Forest Regressor , (d) Support Vector Regressor, (e) Decision Tree Regressor, (f) Gradient Boosting Regressor.

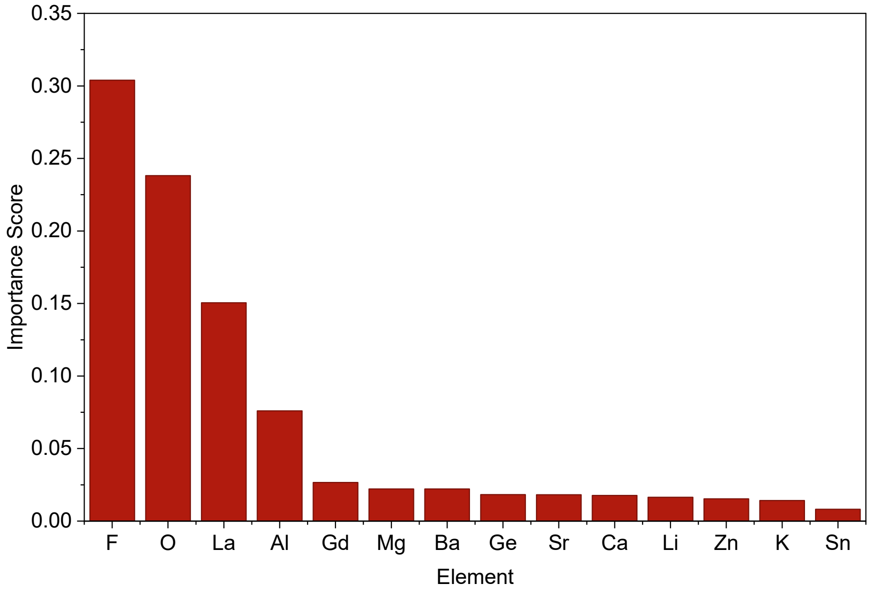

Figure S 4: (Color online) The feature importance of the Extra Trees model
